# Production of Ellagitannin Hexahydroxydiphenoyl Ester by Spontaneous Reduction of Dehydrohexa-hydroxydiphenoyl Ester

**DOI:** 10.3390/molecules25051051

**Published:** 2020-02-26

**Authors:** Manami Era, Yosuke Matsuo, Yoshinori Saito, Takashi Tanaka

**Affiliations:** Department of Natural Product Chemistry, Graduate School of Biomedical Sciences, Nagasaki University, 1-14 Bunkyo-machi, Nagasaki 852-8521, Japan; manamiso912@gmail.com (M.E.); y-matsuo@nagasaki-u.ac.jp (Y.M.); saiyoshi@nagasaki-u.ac.jp (Y.S.)

**Keywords:** dehydrohexahydroxydiphenic acid, hexahydroxydiphenic acid, amariin, geraniin, ellagitannin

## Abstract

Amariin is an ellagitannin with two dehydrohexahydroxydiphenoyl (DHHDP) moieties connecting glucose 2,4- and 3,6-hydroxy groups. This tannin is predominant in the young leaves of *Triadica sebifera* and *Carpinus japonica*. However, as the leaves grow, the 3,6-DHHDP is converted to its reduced form, the hexahydroxydiphenoyl (HHDP) group, to generate geraniin, a predominant ellagitannin of the matured leaves. The purified amariin is unstable in aqueous solution, and the 3,6-(*R*)-DHHDP is spontaneously degraded to give HHDP, whereas 2,4-(*R*)-DHHDP is stable. The driving force of the selective reduction of the 3,6-DHHDP of amariin is shown to be the conformational change of glucose from ^O,3^B to ^1^C_4_. Heating geraniin with pyridine affords 2,4-(*R*)-DHHDP reduction products. Furthermore, the acid hydrolysis of geraniin yields two equivalents of ellagic acid. Although the reaction mechanism is still ambiguous, these results propose an alternative biosynthetic route of the ellagitannin HHDP groups.

## 1. Introduction

Ellagitannin is a group of polyphenols having hexahydroxydiphenoyl (HHDP) ester or its metabolites connected to mostly glucose [1,2]. Due to its high structural diversity and wide distribution in the plant kingdom, ellagitannins continue to attract intense interest within the natural product and organic chemistry communities [3,4]. The accumulation of ellagitannins in some plants suggests that these tannins play a significant role in plant defense systems [5,6,7], while the antiproliferative and antioxidative activities of ellagitannins in fruits and medicinal plants are well studied [8,9,10,11,12,13,14]. However, the biosynthesis of ellagitannins including the production mechanism of the HHDP group remains obscure. The HHDP group was supposed to be formed by oxidative coupling between two galloyl esters on glucopyranose, and this mechanism has been evidenced by the enzymatic preparation of ellagitannin from a gallotannin [15,16,17]. In addition, many successful total ellagitannin syntheses are also based on similar biomimetic strategies [18,19]. However, in 1993, Foo reported an important finding [20]. More specifically, that amariin, 1-*O*-galloyl-2,4;3,6-bis-(*R*)-dehydrohexahydroxydiphenoyl (DHHDP)-β-d-glucose (**1**), degraded spontaneously in aqueous ethanol to give the reduction product geraniin, 1-*O*-galloyl-2,4-(*R*)-DHHDP-3,6-(*R*)-HHDP-β-d-glucopyranose (**2**) as a major product [21,22]. Based on the implicit assumption that polyphenols are susceptible to oxidation, most natural product chemists supposed that DHHDP is generated by the oxidation of HHDP. However, Foo’s finding indicated that HHDP is possibly generated by the reduction of DHHDP. In this study, we investigated the reductive conversion of the DHHDP esters to HHDP esters.

## 2. Results and Discussion

### 2.1. Composition of Ellagitannins in Young and Matured Leaves.

**Figure 1:**
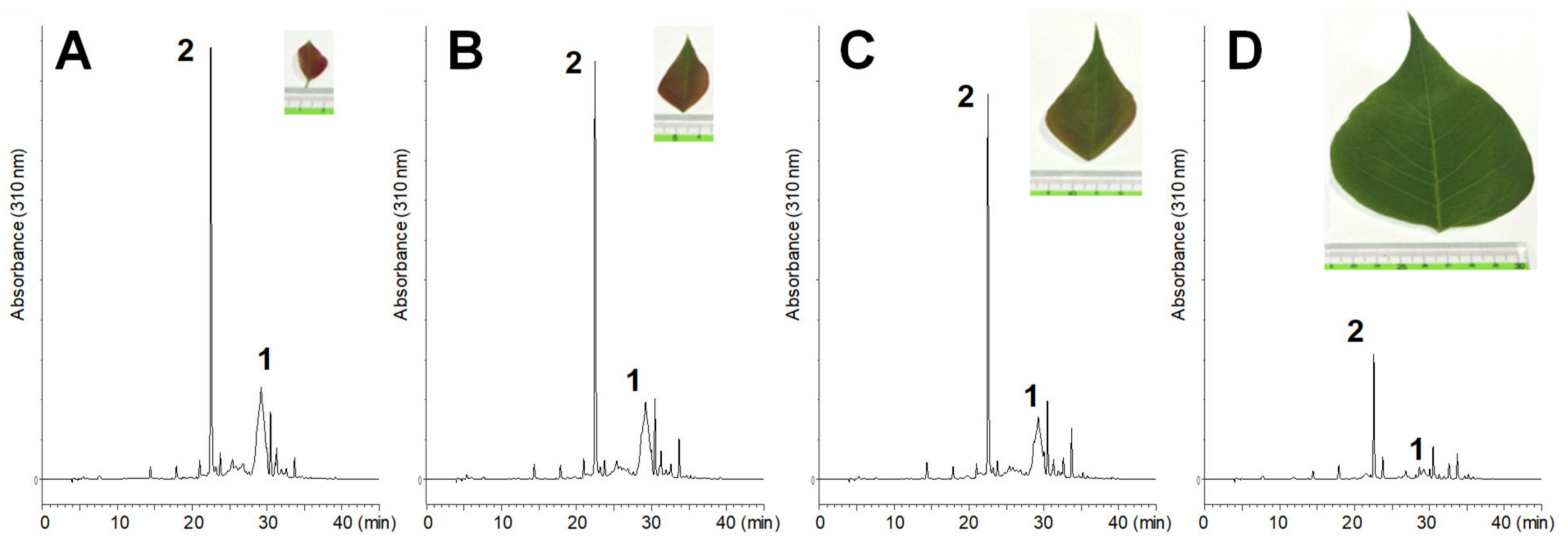
HPLC profiles (310 nm) of extracts of the leaves of *Triadica sebifera*.

**Figure 2:**
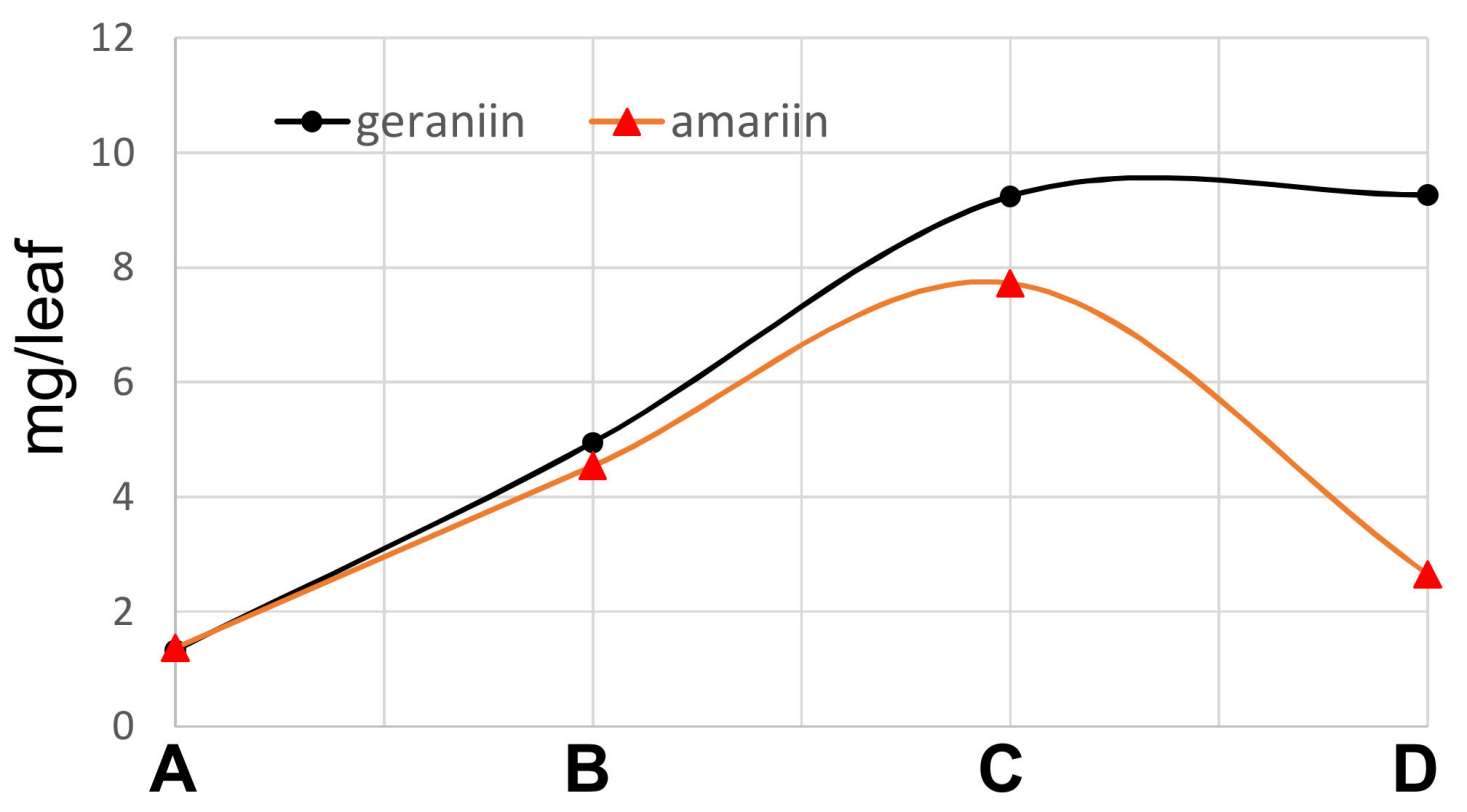
Concentration of amariin (**1**) and geraniin (**2**) per leaf at different growing stages. A–D: see Figure 1.

**Figure 3:**
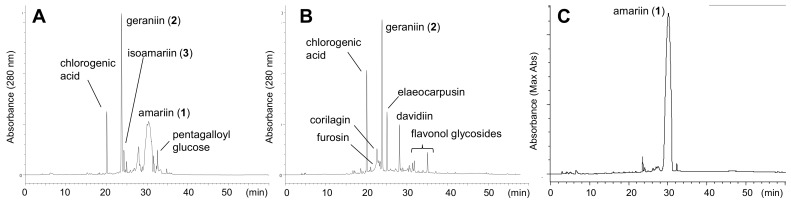
HPLC profiles of extracts of *Carpinus laxiflora* fresh leaves collected in April (**A**) and collected in October (**B**), and amariin (**1**) isolated from the April leaves (**C**).

In our continuous chemical study on ellagitannins, we found that similar reductive production of HHDP from DHHDP occurs in the fresh leaves of *Triadica sebifera* (syn. *Sapium sebiferum*). Amariin (1) was predominant in the small young leaves at the top of the twig collected in June; however, 1 was not detected in the larger and harder leaves of the same twig (Figure 1 and Supplementary Figure S1). Leaves of different sizes, ranging between the smallest leaf at the top and the large matured leaf were lyophilized, and the 60% acetonitrile extracts were analyzed by HPLC. The concentration of geraniin (2) per leaf increased proportionally with the leaf size and reached a plateau (Figure 2). In contrast, the concentration of **1** per leaf first increased as the leaves grew and then decreased as the leaves matured. These observations suggest that **2** is biosynthesized via **1**. Similarly, **1** was predominant in the young leaves of *Carpinus laxiflora* (collected in April) (Figure 3) and decreased as the leaves grew; however, here **2** become predominant instead of **1** in the matured leaves in October [23]. A significant amount of elaeocarpsin, an ascorbic acid adduct of **2**, was also detected in the matured leaves [24]. Furthermore, the young soft leaves of *Elaeocarpus sylvestris* var. *ellipticus* contain **1** as the major ellagitannin accompanied by less **2** content, and the matured, hard leaves contain **2** (Figure S2). These findings also suggest that **1** is a biosynthetic precursor of **2**.

A: young leaf at the top of a twig (leaf size about 1 cm in diameter), D: matured leaf of the same twig (about 8.5 cm in diameter), B (about 2 cm) and C (about 3.5 cm) are the leaves between A and D (50 mg of lyophilized leaf was extracted with 1.5 mL of 60% CH_3_CN, 0.1% TFA).

### 2.2. Structure of Isoamariin

**Figure 4:**
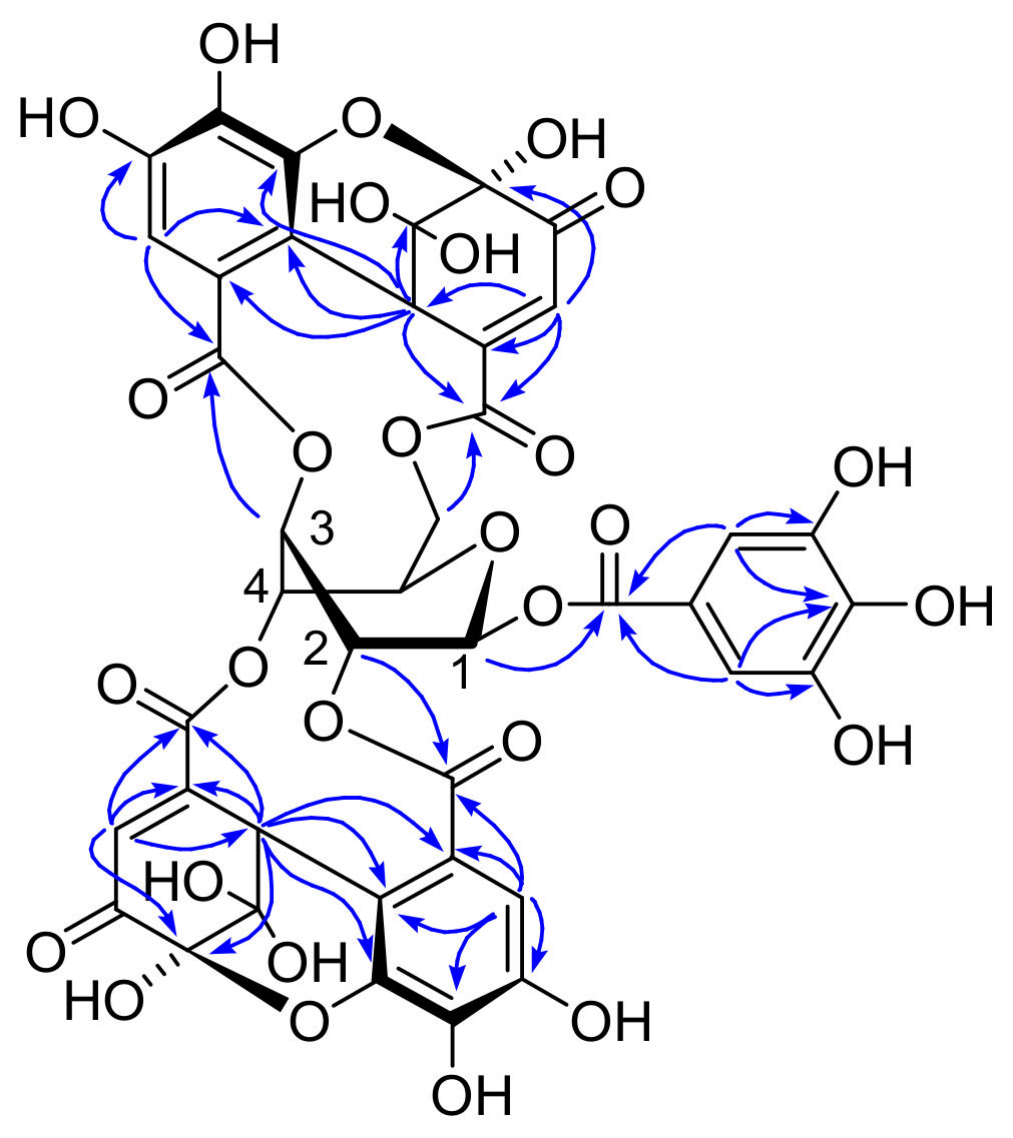
Selected HMBC correlations of major component of **3**.

In the young leaves of *C. laxiflora*, **1** was accompanied by a minor isomer named isoamariin (**3**). The molecular formula C_41_H_28_O_28_ was confirmed by high resolution fast atom bombardment mass spectrometry (HRFABMS) (*m*/*z*: 991.0656 [M + Na]^+^, calcd for C_41_H_28_O_28_Na, 991.0665). The ^1^H and ^13^C NMR spectra are closely related to **1**. They indicated that the molecular parts of **3** are the same as those of **1**. The NMR spectra exhibited duplicated signals caused by an equilibrium between the 6- and 5-membered ring hemiacetal structures of DHHDP group connected to the glucose 2,4-positions. In contrast to the 3,6-DHHDP of **1**, the 3,6-DHHDP of **3** was almost fixed in the 6-membered ring form. In addition, the HMBC correlations (Figure 4) revealed that **3** is an isomer of **1**, differing in the orientation of the 3,6-DHHDP on glucopyranose. The configuration of the DHHDP group was concluded to be *R* based on large negative and positive Cotton effects at 236 and 209 nm, respectively, in the electronic circular dichroism (ECD) spectrum. The structure of ***3*** is the same as that previously reported for didehydrogeraniin [25]; however, the ^1^H NMR chemical shifts of the didehydrogeraniin phenazine derivative in the literature coincided with those of the phenazine derivative of **1** and did not match the data for the phenazine derivative of **3** (Supplementary Table S1).

**Scheme 1:**
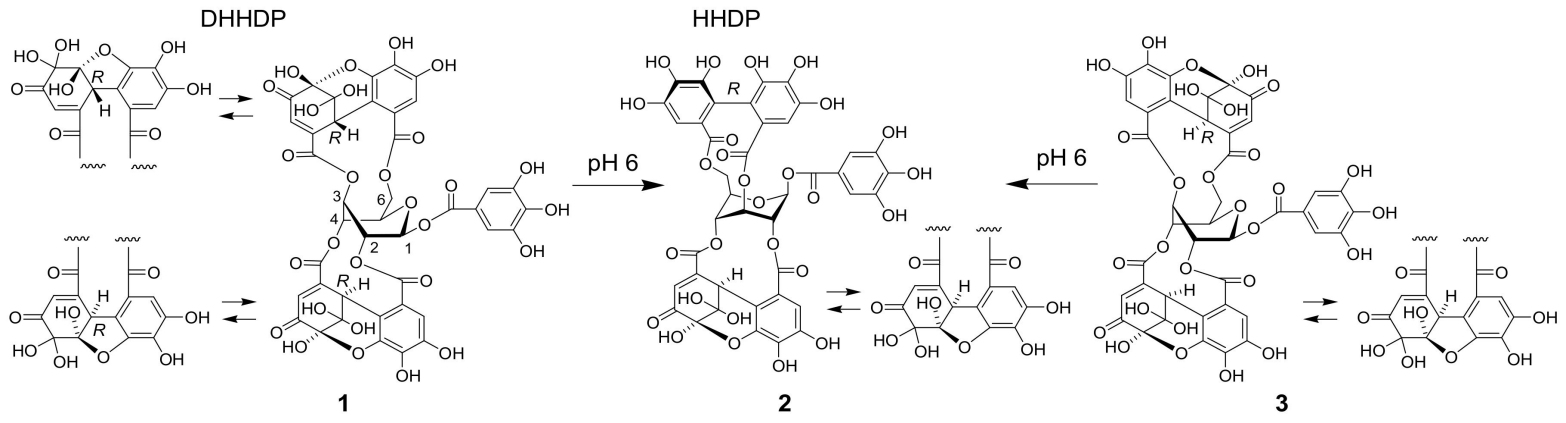
Production of geraniin (**2**) from amariin (**1**) and isoamariin (**3**) in citrate-phosphate buffer (pH 6) at room temperature.

As reported by Foo [20], in aqueous solution **1** decomposed to give reduction product **2** as a major product (Scheme 1, Supplementary Figure S3). Similarly, isoamariin (**3**) was also decomposed under the same conditions to yield **2** (Supplementary Figure S4). Considering that reduction of **1** to **2** proceeded without any reductant, the reaction was presumed to be reduction-oxidation disproportionation. The isolation yield of **2** from **1** in citrate-phosphate buffer at pH 6 was 47%. Other products were identified to be 1-*O*-galloyl-2,4-(*R*)-DHHDP-β-d-glucopyranose (furosin) [25], 1-*O*-galloyl-3,6-(*R*)-HHDP-β-d-glucopyranose (corilagin) [21], gallic acid, ellagic acid, and a new compound, 1-*O*-galloyl-3,6-(*S*)-HHDP-d-glucose (Supplementary Figure S5). The structure of the new compound was determined based on electrospray ionization (ESI)MS, HRFABMS, ^1^H, ^13^C NMR, ^1^H-^1^H correlation spectroscopy (COSY), heteronuclear single quantum coherence (HSQC), heteronuclear multiple bond correlation (HMBC) and ECD spectra as shown in the Supplementary Materials (Figure S6). The 3,6-(*S*)-HHDP glucose is rare in nature; however, 1,2,4-tri-*O*-galloyl-3,6-(*S*)-HHDP-α-d-glucose is one of the major ellagitannin of *Nuphar japonicum* [26]. At the present time, we have not succeeded in identifying the oxidation products that are expected to be generated together with the reduction products.

**Figure 5:**
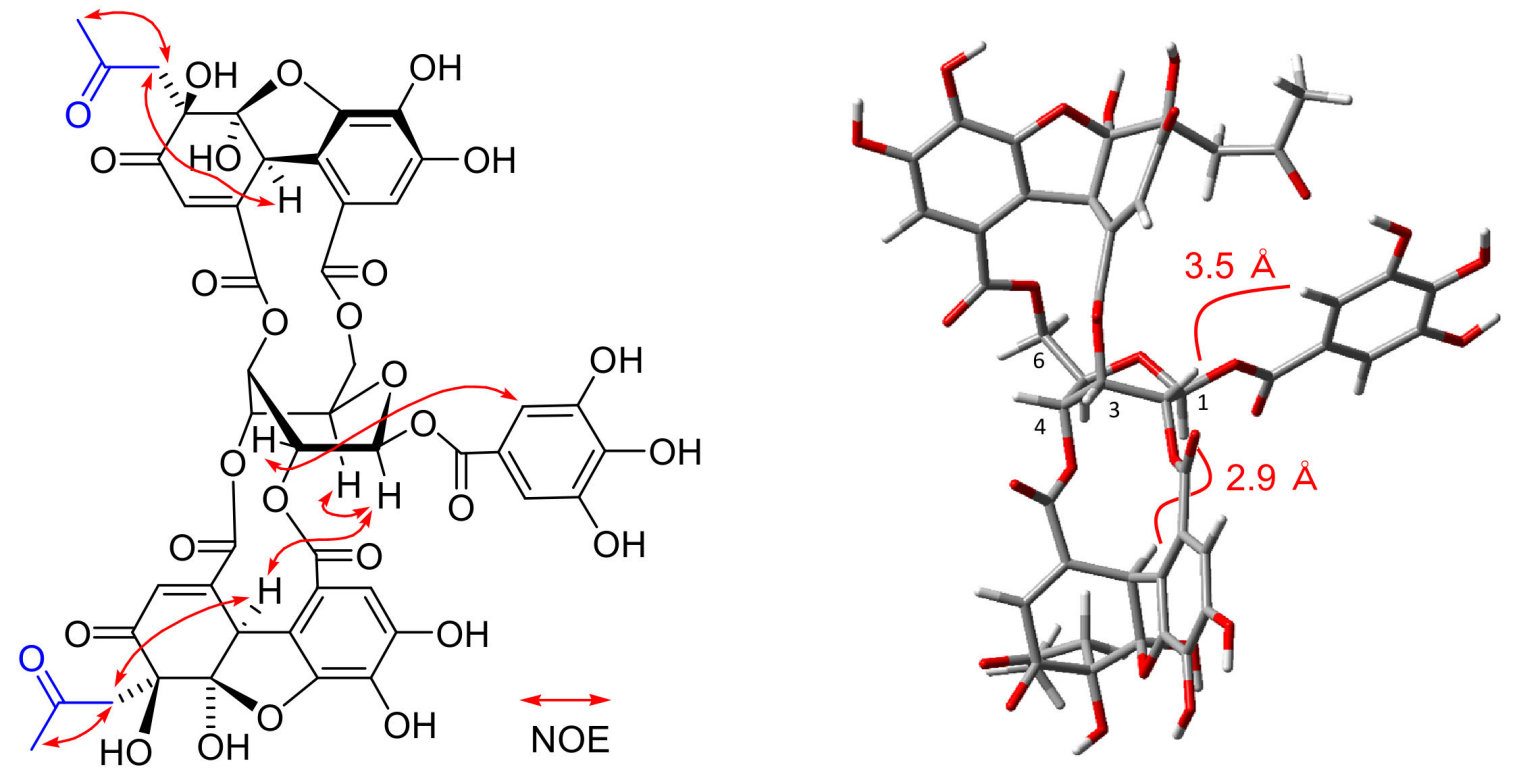
Selected NOE correlations and stereostructure obtained by DFT calculation of **1a**.

Only the 3,6-DHHDP of the two DHHDP groups of **1** selectively undergo the reduction; however, computational calculation indicated that the structures of the 2,4- and 3,6-DHHDP moieties are both ridged and essentially the same. The difference of the reactivity is thought to be caused by a difference in the strain from glucose to the DHHDP groups. The coupling constants of glucopyranose (*J*_1,2_ = 5.1 Hz, *J*_2,3_ = 1.3 Hz, *J*_3,4_ = 3.7 Hz, and *J*_4,5_ = 1.5 Hz) suggest that conformation of the glucopyranose of **1** is of the ^O,3^B form. In contrast, X-ray crystallography confirmed that the glucopyranose core of **2** adopts the ^1^C_4_ conformation [27]. Due to the difficulty in analyzing the NMR signals caused by the equilibrium of the DHHDP groups, a bisacetonyl derivative **1a** was prepared to fix the DHHDP groups in the 5-membered ring hemiacetal form [28], and DFT calculation was applied to **1a**. The coupling constants observed for glucopyranose of **1a** were essentially the same as those of **1**, and they were also in agreement with those of **1a** obtained by DFT calculation. The experimental NOEs observed for **1a** agreed with the calculation results (Figure 5). Calculation also indicated that a conformer with ^1^C_4_-glucopyranose is less stable than in the ^O,3^B form (ΔG = + 4.5 kcal/mol). In contrast, **2** prefers the ^1^C_4_ conformation, and 1-*O*-galloyl-2,4-(*R*)-DHHDP-β-d-glucose is also shown to be adapted to ^1^C_4_ conformation based on the coupling constants and DFT calculation. These observations suggested that the difference in the reactivity of 3,6- and 2,4-DHHDP of **1** was due to the difference of strain present from the glucose to ester carbonyl groups.

**Scheme 2:**
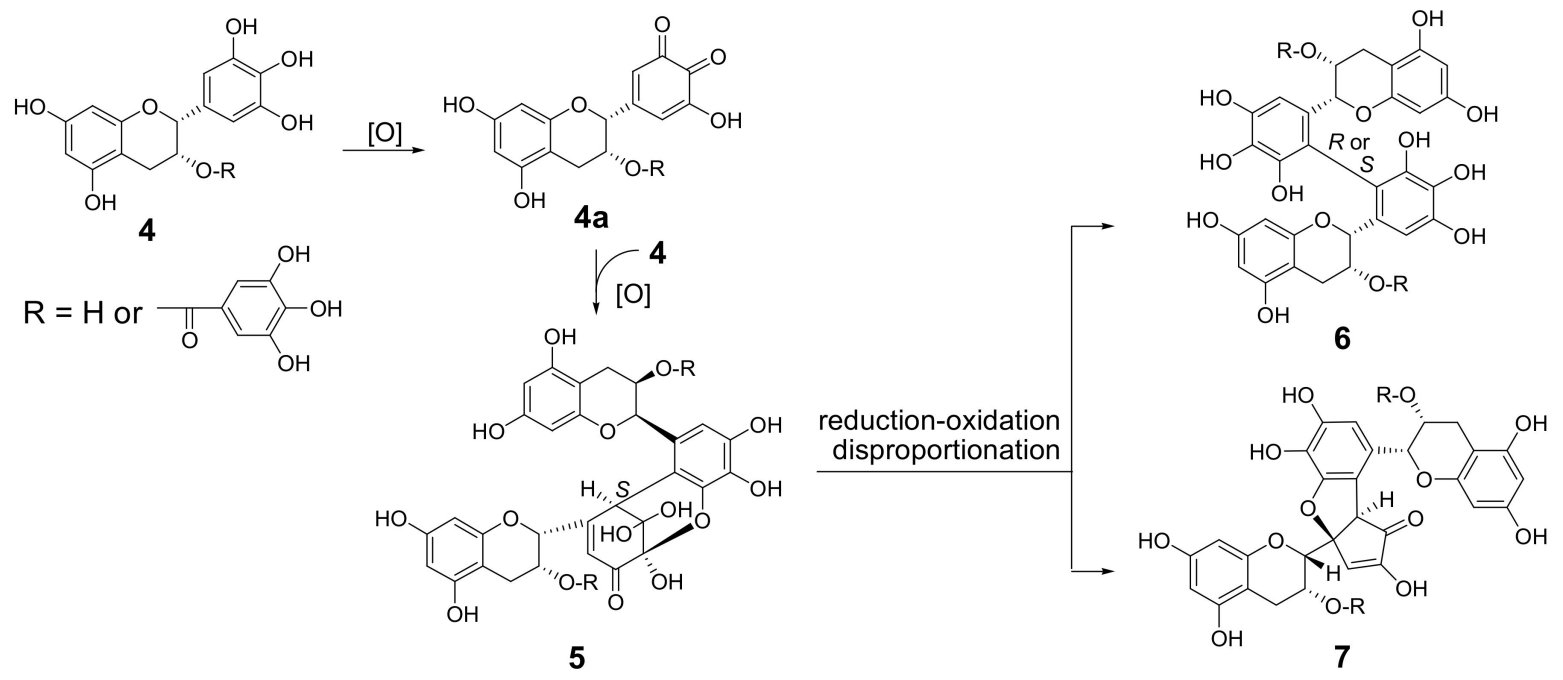
Reduction-oxidation disproportionation of catechin quinone dimer during black tea production.

DHHDP is regarded as a hydrated quinone dimer of galloyl groups. Similar pyrogallol-quinone dimers of epigallocatechin (**4**), that is, dehydrotheasinensins (**5**) are produced during the tea fermentation process in black tea manufacturing (Scheme 2) [29,30]. The catechin dimers are unstable and decompose during the drying process of black tea production to generate reduction products, theasinensins (**6**), as the major products. Its hexahydroxybiphenyl structure is structurally related to the HHDP group of ellagitannins. Experiments using pure dehydrotheasinensins showed that the reaction is reduction-oxidation disproportionation. However, only limited oxidation products, such as oolongtheanins (**7**) [31], were identified due to the complexity of the oxidation products. Theasinensins are catechin oxidation products characteristic of black tea, and the dimerization process seems to be related to the 3,6-HHDP formation of **2**. The aforementioned production of the 3,6-(*S*)- and 3,6-(*R*)-HHDP glucoses from the (*R*)-DHHDP of **1** was also related to the production of theasinensins with *R* and *S* biphenyl bond, respectively, from (*S*)-dehydrotheasinensins.

**Figure 6:**
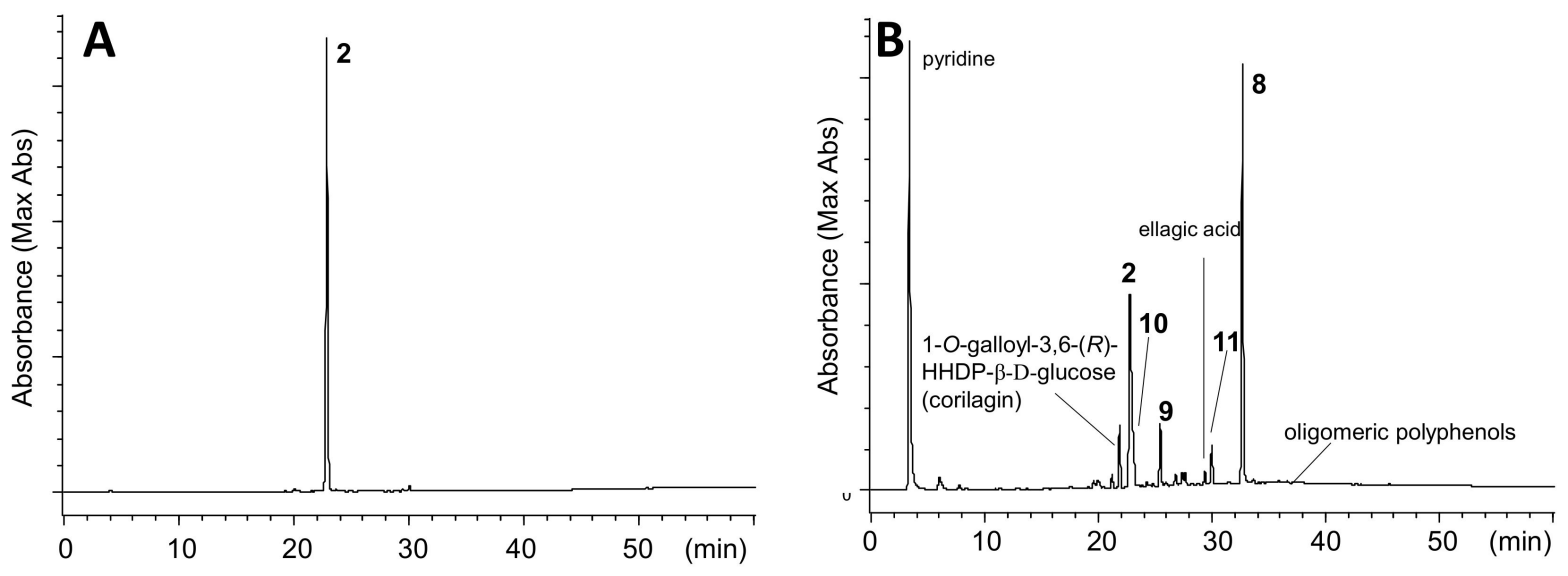
HPLC profiles of geraniin (**2**) (**A**) and reaction mixture after treatment with 4% pyridine in CH_3_CN (80 ˚C, 90 min) (**B**).

**Figure 7:**
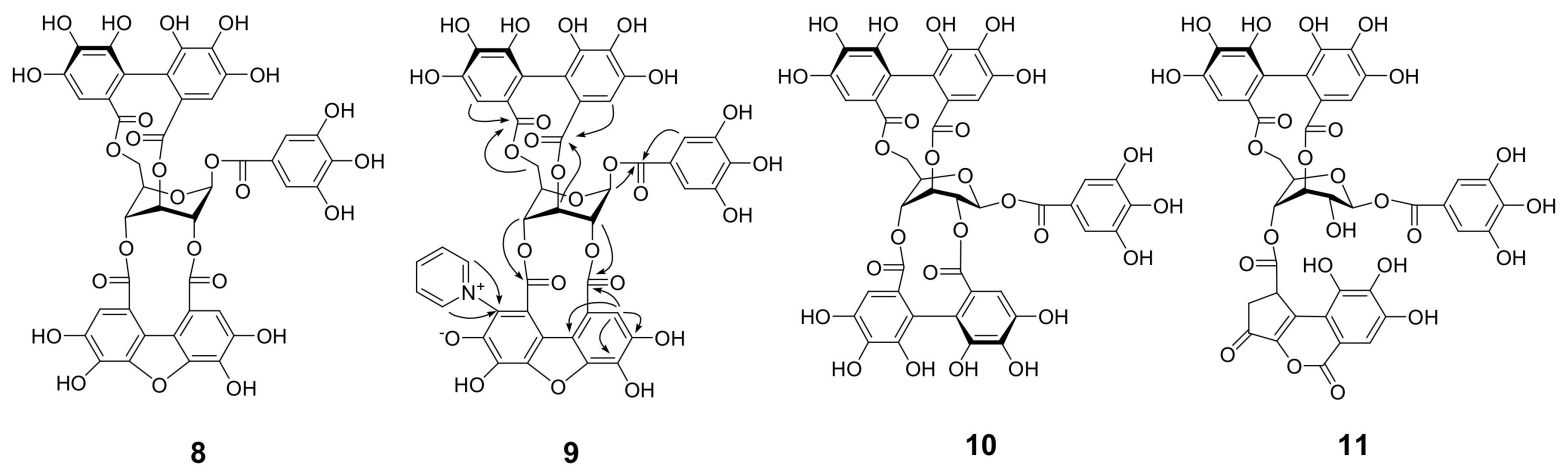
Products obtained by treatment of **2** with pyridine in CH_3_CN (80 °C, 90 min) and HMBC correlations of **9**.

**Scheme 3:**
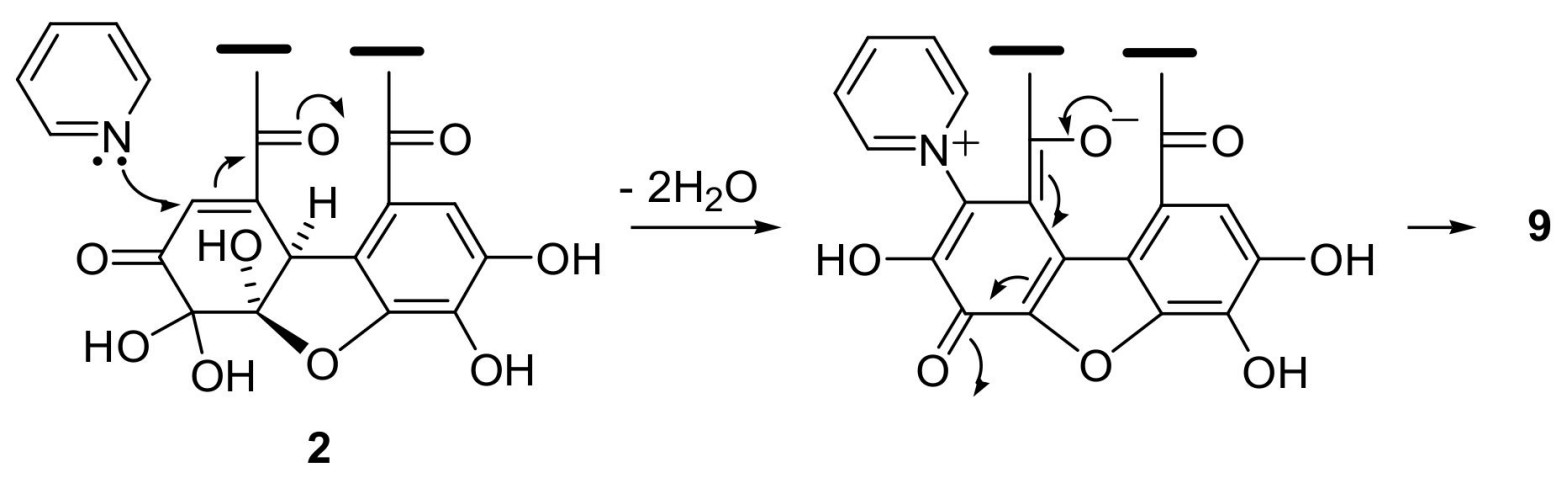
Plausible production mechanism of **9** from **2**.

Previously, we reported that heating of **2** with pyridine in CH_3_CN yielded reduction products mallotusinin (**8**) with a characteristic dibenzofuran structure, 1-*O*-galloyl-2,4;3,6-bis-(*R*)-HHDP-β-d-glucose (**10**), and a decarboxylation product (**11**) [32]. The major product was **8** (30%, recovery of **2** was 15%)(Figure 6). In this study, an additional product **9** was isolated. The ^1^H and ^13^C NMR spectra indicated that **9** is a pyridine adduct of **8**. The binding site of the pyridine was confirmed to be at the glucose C-4 side of the dibenzofuran moiety by HMBC correlations of pyridinium H-2,6 with the benzofuran 3’ carbon (Figure 7). This was supported by a large up field shift of the glucose H-4 (Δδ 0.67) as compared to that of **8**. The production mechanism of **9** from **2** is proposed as shown in Scheme 3, and this may be related to the reduction of **2** to **8**. The attack of hydride instead of pyridine produces **8**.

In this experiment, we could not identify the expected oxidation products; however, oligomeric products (isolation yield 9.4%) may be candidates. The mixture of oligomers was detected at the origin during thin layer chromatography (TLC) and as a broad hump on the baseline of the HPLC (Figure 6 and Figure S7). The ^13^C NMR spectrum showed signals attributable to galloyl and HHDP esters together with glucose (Figure S8). Methylation and alkaline degradation of the oligomers confirmed the presence of galloyl, HHDP, and tetrahydroxy dibenzofuran moieties; however, we could not obtain evidence that the oligomers are oxidation products of **2**.

Similar but contradictory results were obtained during acid hydrolysis. Foo reported that acid hydrolysis of amariin (**1**) gave ellagic acid [20], and Okuda et al. also indicated the production of excess ellagic acid upon acid hydrolysis of **2** [22]. These results suggested that reduction of the DHHDP esters also occurs during hydrolysis. Reexamined the acid hydrolysis of **2** with 2% H_2_SO_4_ at 100 ˚C afforded ellagic acid in the isolation yield of 180 mol%. Furthermore, the acid hydrolysis of 1-*O*-galloyl-2,4-(*R*)-DHHDP-β-d-glucopyranose with 2% H_2_SO_4_ also yielded gallic acid and ellagic acid in the molar ratio of 1:0.73 (Supplementary Figure S9). These results suggested that the reduction of DHHDP to HHDP during the hydrolysis is not reduction-oxidation disproportionation. In this reaction, a possible electron donor candidate was glucose simultaneously generated by the hydrolysis; however, the ^13^C NMR spectrum and TLC of the sugar fraction obtained by hydrolysis confirmed the recovery of glucose.

## 3. Materials and Methods

### 3.1. General

Ultraviolet (UV) spectra were obtained on a JASCO V-560 UV/VIS spectrophotometer (JASCO, Tokyo, Japan). Optical rotations were measured with a JASCO DIP-370 digital polarimeter. The ECD spectra were measured with a JASCO J-725N spectrophotometer. ^1^H and ^13^C NMR spectra were recorded on a Varian Unity Plus 500 spectrometer (Agilent Technologies, Santa Clara, CA, USA) operating at 500 and 125 MHz for the ^1^H and ^13^C nuclei, respectively. NMR spectra were also recorded on a JEOL JNM-AL 400 spectrometer (JEOL Ltd., Tokyo, Japan) operating at 400 and 100 MHz for the ^1^H and ^13^C nuclei, respectively. ESIMS were obtained using a JEOL JMS-T100TD spectrometer. FABMS were recorded on a JMS700N spectrometer (JEOL Ltd.) using *m*-nitrobenzyl alcohol or glycerol as the matrix. Column chromatography was performed using Sephadex LH-20 (25–100 mm, GE Healthcare UK Ltd., Little Chalfont, UK), MCI-gel CHP20P (75–150 mm, Mitsubishi Chemical Co., Tokyo, Japan), Diaion HP20SS (Mitsubishi Chemical Co.), Toyopearl Butyl-650C (Tosoh Bioscience Japan, Tokyo, Japan), Cosmosil 75C18OPN (Nacalai Tesque Inc., Kyoto, Japan), and Chromatorex ODS (Fuji Silysia Chemical Ltd., Kasugai, Japan) columns. TLC was performed on precoated Kieselgel 60 F_254_ plates (0.2-mm thickness, Merck, Darmstadt, Germany), using toluene–ethyl formate–formic acid (1:7:1, *v*/*v*) and CHCl_3_–MeOH–H_2_O (7:3:0.5, *v*/*v*) mixtures as the eluents. The spots were detected using ultraviolet illumination and by spraying with 2% ethanolic FeCl_3_ solution (for phenolic compounds), 5% anisaldehyde in ethanolic 5% H_2_SO_4_ solution (for proanthocyanidins), or 5% H_2_SO_4_ solution followed by heating. Analytical HPLC was performed on a Cosmosil 5C18-ARII (Nacalai Tesque Inc., Kyoto, Japan) column (250 × 4.6 mm, i.d.) with a gradient elution of 4–30% (39 min) and 30–75% (15 min) CH_3_CN in 50 mM H_3_PO_4_ at 35 °C (flow rate, 0.8 mL/min; detection, JASCO photodiode array detector MD-2018 plus). Geraniin was obtained as yellow crystalline powder from previous our studies [33].

### 3.2. Plant Material

Fresh leaves of *Triadica sebifera* and *Elaeocarpus sylvestris* var. *ellipticus* were collected in Bunkyo campus of Nagasaki University. Fresh leaves of *Carpinus laxiflora* were collected Mt. Gokahara, Isahaya, Nagasaki prefecture.

### 3.3. Quantification of Ellagitannins in the Leaves of T. Sebifera

Fresh leaves of *T. sebifera* were lyophilized and powdered by grinding in a mortar. Aliquots (50 mg) of the powder (each 4 samples) were extracted with 60% CH_3_CN containing 0.1% trifluoroacetic acid (1.5 mL) at room temperature for 7 h. The extracts were analyzed by HPLC. Calibration curves were built using geraniin (320 nm) isolated in our previous study [24] and amariin (275 nm) isolated in this study. Average dried weights of the leaves in the Figure 1 were as follows: A, 0.007 g/leaf (fresh: 0.022 g/leaf); B, 0.029 g/leaf (fresh: 0.089 g/leaf); C, 0.065g/leaf (fresh: 0.20 g/leaf), and D, 0.195 g/leaf (fresh: 0.63 g/leaf).

### 3.4. Isolation of Amariin (**1**) and Isoamariin (**3**)

Fresh young leaves of *Carpinus laxiflora* (750 g) collected in April 30, 2014 were homogenized with 80% acetone containing 0.005% trifluoroacetic acid (TFA) (3 L) at room temperature 2 times. The extract was filtered, and the filtrate was concentrated using rotary evaporator. Resulting precipitates formed in the aqueous solution were removed by filtration. The filtrate (about 800 mL) was applied to Diaion HP20SS (8 cm i.d. × 37 cm) column with 0.005% TFA containing increasing proportions of MeOH [0% (500 mL), 20% (1 L), 30% (1 mL), 40% (1 L), 50% (1 L), 60% (1 L), 80% (1 L), and 100% (2 L)] to give 5 fractions. Fraction (Fr.) 3 (16.8 g) mainly containing **1** was subjected to Sephadex LH-20 column chromatography (5 cm i.d. × 22 cm) with 0%–100% MeOH in 0.005% TFA (20% stepwise, each 300 mL) to yield a crude sample of **1** (10.9 g). A portion (7.5 g) of the crude sample was purified by Diaion HP20SS column chromatography (4 cm i.d. × 27 cm, 0%–60% MeOH in 0.005% TFA, 5% stepwise, each 200 mL). Tubes containing **1** were combined and concentrated by rotary evaporator (40 ˚C) to give yellow precipitates of **1**, which were collected by filtration (3.7 g). The filtrate (0.76 g) was separated by Chromatorex ODS (3 cm i.d. × 25 cm, 0%–50% MeOH in 0.005% TFA, 5% stepwise, each 100 mL) to yield **3** (121 mg).

*Amariin* (**1**): Yellow powder, ^1^H NMR (500 MHz, acetone-*d*_6_) δ: galloyl: 7.23, 7.22 (each s, galloyl-2,6); DHHDP (5- and 6-membered-ring hemiacetal structure): 7.26, 7.238, 7.236, 7.235, 7.233 (each s, H-3**’**), 6.71, 6.67, 6.60, 6.58 [each s, H(6)-3], 6.31, 6.26 [each d, *J* = 1.5 Hz, H(5)-3], 5.31. 5.29, 5.27, 5.26 [each s, H(6)-1], 4.90, 4.88, 4.86, 4.85 [each d, *J* = 1,5 Hz, H(5)-1]; glucose (two major isomers among 4 isomers), isomer A: 6.40 (d, *J* = 5.4 Hz, glc-H-1), 5.79 (dd, *J* = 1.5, 3.2 Hz, glc-3), 5.50 (br d, *J* = 3.2 Hz, glc-4), 5.47 (dt, *J* = 1.5, 5.4 Hz, glc-2), 5.23 (dd, *J* = 2.7, 13.3 Hz, glc-6), 4.77 (br s, glc-5), 4.39 (br d, *J* = 13.3 Hz, glc -6); isomer B: 6.35 (d, *J* = 5.1 Hz, glc-1), 5.65 (dt, *J* = 1.3, 3.7 Hz, glc-4), 5.62 (dt, *J* = 1.5, 5.1 Hz, glc-2), 5.53 (dd, *J* = 1.5, 3.7 Hz, glc-3), 5.33 (dd, *J* = 2.6, 13.3 Hz, glc-6), 4.83 (br s, glc-5), 4.39 (br d, *J* = 13.3 Hz, glc -6); ^13^C NMR (125 MHz, acetone-*d*_6_) δ: only signals of major isomers COO: 168.4, 168.1, 166.2, 165.1, 165.0, 164.8, 164.7, 164.6, 164.4; galloyl: 146.21, 146.18 (C-3,5), 139.9, 139.2 (C-4), 110.40, 110.39 (C-2,6); DHHDP: 194.2 [C(5)-4], 191.6, 191.2 [C(6)-4], 153.6, 152.5, 152.1 [C(6)-2], 148.6, 147.8. 147.3 [C(5)-6**’**], 143.5, 143.0, 142.9 [C(6)-6**’**], 129.2, 128.6 [C(6)-3], 125.7 [C(5)-3], 96.6, 96.2 [C(6)-5], 92.31, 92.27, 92.0 [C(5)-5, C(6)-6], 52.2, 51.1 [C(5)-1], 46.4, 45.0, 44.8 [C(6)-1]; other aromatic and sp^2^ carbons: 145.93, 145.86, 145.83, 139.2, 138.5, 138.4, 137.6, 120.3, 120.0, 119.9, 119.8, 119.7, 118.7, 117.0, 116.0, 115.0, 114.9, 113.9, 113.6, 113.1; glucose (two major isomers among 4 isomers): 95.7, 94.4 (glc-1), 78.1, 77.7 (glc-5), 73.7, 72.7 (glc-2), 70.4, 69.7 (glc-4), 67.6,66.1 (glc-3), 66.0, 65.8 (glc-6).

*Isoamariin* (**3**): Yellow powder, [α${]}_{D}^{17}$ −136.8 (*c* 0.1, MeOH); FAB-MS *m*/*z*: 991 [M + Na]^+^; HR-FAB-MS *m*/*z*: 991.0656 [M + Na]^+^ (calcd. for C_41_H_28_O_28_Na, 991.0665); IR ν_max_ cm^−1^: 3413, 1705, 1613, 1528, 1447, 1325, 1213; UV (MeOH) λ_max_ (log ε): 279 (4.43), 221 (4.89); ECD (MeOH) λ_max_ (Δε): 369 (−3.9), 326 (−1.4), 293 (−8.6), 270 (−2.1), 262 (−2.4), 257 (−2.1), 236 (−14.7), 228 (0), 209 (+36.4). ^1^H NMR (500 MHz, acetone-*d*_6_) δ: galloyl: 7.21, 7.20 (each s, galloyl-2,6); 2,4-DHHDP 5-membered-ring hemiacetal structure: 7.29 (s, H-3**’**), 6.26 (d, *J* = 1.4 Hz, H-3), 4.95 (d, *J* = 1.4 Hz, H-1); 2,4-DHHDP 6-membered-ring hemiacetal structure: 7.27 (s, H-3**’**), 6.60 (s, H-3), 5.35 (s, H-1); glucose with 5-membered-ring hemiacetal 2,4-DHHDP: 6.42 (d, *J* = 5.2 Hz, glc-1), 5.67 (dt, *J* = 1.3, 5.2 Hz, glc-2), 5.51 (dd, *J* = 1.3, 3.7 Hz, glc-3), 6.17 (dt, *J* = 1.3, 3.7 Hz, glc-4), 4.72 (br s, glc-5), 4.60 (dd, *J* = 3.1, 13.3 Hz, glc-6), 4.90 (dd, *J* = 1.7, 13.3 Hz, glc -6); glucose with 6-membered-ring hemiacetal 2,4-DHHDP: 6.36 (d, *J* = 4.7 Hz, glc-1), 5.78 (dt, *J* = 1.4, 4.7 Hz, glc-2), 5.36 (dd, *J* = 1.4, 3.7 Hz, glc-3), 6.28 (dt, *J* = 1.3, 3.5 Hz, glc-4), 4.76 (br s, glc-5), 4.62 (dd, *J* = 3.1, 12.3 Hz, glc-6), 4.93 (dd, *J* = 1.8, 12.3 Hz, glc-6); ^13^C NMR (125 MHz, acetone-*d*_6_) δ: galloyl: 119.7, 119.9 (galloyl-1), 110.3 (galloyl-2,6), 146.2 (galloyl-3,5), 139.8, 139.9 (galloyl-4), 164.8, 164.9 (galloyl-7); 2,4-DHHDP 5-membered-ring hemiacetal structure: 52.1 (C-1), 148.9 (C-2), 125.8 (C-3), 194.3 (C-4), 92.4 (C-5), 109.3 (C-6), 117.1 (C-1**’**), 120.3 (C-2**’**), 113.1 (C-3**’**), 147.8 (C-4**’**), 137.6 (C-5**’**), 147.3 (C-6**’**); 2,4-DHHDP 6-membered-ring hemiacetal structure: 46.4 (C-1), 154.0 (C-2), 129.3 (C-3), 191.7 (C-4), 96.2 (C-5), 92.4 (C-6); 116.1 (C-1**’**), 119.0 (C-2**’**), 113.6 (C-3**’**), 145.9 (C-4**’**), 139.2 (C-5**’**), 144.0 (C-6**’**); glucose with 5-membered-ring hemiacetal 2,4-DHHDP: 94.3 (glc-1), 74.2 (glc-2), 67.5 (glc-3), 69.6 (glc-4), 77.1 (glc-5), 68.0 (glc-6); glucose with 6-membered-ring hemiacetal 2,4-DHHDP: 95.4 (glc-1), 73.6 (glc-2), 69.0 (glc-3), 68.7 (glc-4), 76.5 (glc-5), 67.9 (glc-6).

### 3.5. Preparation of Acetonyl Derivative

Amariin (**1**) (130 mg) was dissolved in 80% acetone (20 mL) containing HCO_2_NH_4_ (130 mg) and stirred at r.t. for 13h. After removal of acetone by evaporation, the aqueous solution was subjected to Diaion HP20SS column chromatography (2 cm i.d. × 15 cm, 0%–60% MeOH in H_2_O, 5% stepwise, each 50 mL) to yield **1a** (57.2 mg).

*Bisacetonyl derivative* (**1a**): off-white amorphous powder, [α${]}_{D}^{17}$ −55.2 (c 0.1, MeOH); IRν_max_ cm^−1^: 3406, 1715, 1620, 1531, 1441; UV (MeOH) λ_max_ (log ε): 282 (4.56), 221 (4.91). ^1^H NMR (500 MHz, acetone-*d*_6_) δ: ^1^H-NMR (acetone-*d*_6_, 500 MHz) δ: 2.19 (3H, s, 2,4-acyl-CH_3_), 2.23 (3H, s, 3,6-acyl-CH_3_), 2.96 (1H, d, *J* = 15.3 Hz, 3,6-acyl-CH_2_), 3.06 (1H, d, *J* = 15.6 Hz, 2,4-acyl-CH_2_), 3.46 (1H, d, *J* = 15.6 Hz, 2,4-acyl-CH_2_), 3.48 (1H, d, *J* = 15.3 Hz, 3,6-acyl-CH_2_), 4.21 (1H, dd, *J* = 1.2, 13.1 Hz, glc-6a), 4.83 (1H, d, *J* = 1.5 Hz, 3,6-acyl-1), 4.88 (1H, br. s, glc-5), 4.98 (1H, d, *J* = 1.5 Hz, 2,4-acyl-1**’**), 5.39 (1H, dt, *J* = 1.4, 3.8 Hz, glc-4), 5.47 (1H, dt, *J* = 1.5, 4.9 Hz, glc-2), 5.57 (1H, dd, *J* = 3.2, 13.1 Hz, glc-6b), 5.70 (1H, dd, *J* = 1.4, 3.8 Hz, glc-3), 6.35 (1H, d, *J* = 1.5 Hz, 2,4-acyl-3**’**), 6.44 (1H, d, *J* = 4.9 Hz, glc-1), 6.46 (1H, d, *J* = 1.5 Hz, 3,6-acyl-3), 7.20 (1H, s, 3,6-acyl-3**’**), 7.22 (1H, s, 2,4-acyl-3), 7.27 (2H, s, galloyl-H); ^13^C NMR (acetone-*d*_6_, 125 MHz) δ: 32.0 (2,4-acyl-CH_3_), 32.2 (3,6-acyl-CH_3_), 49.2 (3,6-acyl-CH_2_), 49.9 (2,4-acyl-CH_2_), 50.8 (3,6-acyl-1), 51.9 (2,4-acyl-1**’**), 64.5 (glc-6), 66.0 (glc-3), 69.7 (glc-4), 73.8 (glc-2), 78.1 (glc-5), 80.2 (3,6-acyl-5), 80.6 (2,4-acyl-5**’**), 94.3 (glc-1), 110.0 (2,4-acyl-6**’**), 110.4 (galloyl-2, 6), 110.6 (3,6-acyl-6), 113.0 (2,4-acyl-3, 3,6-acyl-3**’**), 116.9 (2,4-acyl-1), 117.9 (3,6-acyl-1**’**), 119.9 (3,6-acyl-2**’**), 120.1 (galloyl-1), 120.5 (2,4-acyl-2), 127.1 (3,6-acyl-3), 127.5 (2,4-acyl-3**’**), 136.5 (3,6-acyl-5**’**), 137.3 (2,4-acyl-5), 139.6 (galloyl-4), 144.6 (3,6-acyl-2), 144.9 (2,4-acyl-2**’**), 146.1 (galloyl-3, 5), 146.7 (3,6-acyl-6**’**), 147.0 (2,4-acyl-6), 147.4 (2,4-acyl-4, 3,6-acyl-4**’**), 164.3 (2,4-acyl-7), 164.9 (galloyl-7, 3,6-acyl-7), 166.1 (2,4-acyl-7**’**), 166.9 (3,6-acyl-7**’**), 196.9 (3,6-acyl-4), 197.2 (2,4-acyl-4**’**), 205.7 (2,4-acyl-CO), 205.9 (3,6-acyl-CO).

### 3.6. Preparation of Phenazine Derivative

Amariin (**1**) (50 mg) and *o*-phenylenediamine (10 mg) was dissolved in 10% AcOH in EtOH (2 mL) and heated at 50 ˚C for 2 h. The mixture was applied to Sephadex LH-20 (2 cm i.d. × 15 cm, 0-20% H_2_O in EtOH, 10% stepwise, each 100 mL) to yield **1b** (30.2 mg). Phenazine derivative **3a** was prepared from **3** in the same way.

*Phenazinde derivative* (**1b**): UV (MeOH) λmax (log ε) 445 (3.56), 378 (4.13), 281 (4.98), 205 (4.87); ECD (MeOH) Δε (nm) 377 (−1.48), 325 (+3.86), 284 (−67.5), 250 (+72.1), 221 (−42.0). ^1^H NMR (acetone-*d*_6_, 500 MHz) δ: 4.11 (1H, dd, J = 3.3, 12.0 Hz, glc-6a), 4.98 (1H, dd, J = 8.1, 12.0, glc-6b), 5.04 (1H, dd, J = 3.3, 8.1, glc-5), 5.40 (1H, d, J = 4.1, glc-4), 5.80 (1H, d, J = 4.1, glc-3), 5.86 (1H, d, J = 5.5, glc-2), 6.23 (1H, d, J = 5.5, glc-1), 7.01 (2H, s, galloyl-H), 7.03 (1H, s, 3,6-acyl-3**’**), 7.52 (1H, s, 2,4-acyl-3), 8.24 (1H, s, 3,6-acyl-3), 8.31 (1H, s, 2,4-acyl-3**’**); ^13^C-NMR (acetone-*d*_6_, 125 MHz) δ: 64.7 (glc-6), 67.7 (glc-3), 68.3 (glc-4), 76.2 (glc-2), 77.0 (glc-5), 91.7 (glc-1), 109.7 (2,4-acyl-3), 110.1 (galloyl-2, 6), 113.2 (3,6-acyl-3**’**), 115.2 (3,6-acyl-1**’**), 116.4 (2,4-acyl-1), 116.7 (3,6-acyl-1), 116.8 (2,4-acyl-1**’**), 119.8 (galloyl-1), 120.1 (2,4-acyl-3**’**), 120.6 (2,4-acyl-2), 120.7 (3,6-acyl-3), 123.1 (3,6-acyl-2**’**), 130.0, 130.4, 130.5 (2,4-acyl-4**”**, 5**”**, 3,6-acyl-4**”**, 5**”**), 132.2, 132.3 (2,4-acyl-3**”**, 6**”**, 3,6-acyl-3**”**, 6**”**), 136.0 (2,4-acyl-2**’**), 136.3 (3,6-acyl-2), 137.4 (3.6-acyl-5**’**), 138.0, 139.0 (2,4-acyl-5**’**, 3,6-acyl-5), 139.3 (2,4-acyl-5), 139.6 (galloyl-4), 142.8, 143.0, 143.1, 145.1, 145.3, 145.4, 145.6, 145.9 (2,4-acyl-6, 4**’**, 1**”**, 2**”**, 3,6-acyl-4, 4**’**, 6**’**, 1**”**, 2**”**), 145.3 (2,4-acyl-4), 146.0 (galloyl-3, 5), 152.1 (3,6-acyl-6), 152.3 (2,4-acyl-6**’**), 164.7 (galloyl-7), 165.9 (3,6-acyl-7), 166.6 (2,4-acyl-7**’**), 167.8 (3,6-acyl-7**’**), 167.9 (2,4-acyl-7).

*Phenazine derivative* (**3a**): Brown amorphous powder, [α${]}_{D}^{17}$ −323.9 (*c* 0.06, MeOH); FAB-MS *m*/*z*: 1077 [M + H]^+^, 1099 [M + Na]^+^; HR-FAB-MS *m*/*z*: 1099.1403 [M + Na]^+^, (calcd. for C_53_H_32_O_22_N_4_Na, 1099.1406); IR *v*_max_ cm^−1^: 3336, 1732, 1613, 1509, 1463, 1355, 1335, 1308, 1189; UV (MeOH) λ_max_ (log ε): 446 sh (3.51), 375 (4.15), 279 (5.00), 223 sh (4.86), 207 (4.89); ECD (MeOH) λ_max_ (Δε): 381 (+3.8), 359 (+1.3), 304 (+16.9), 292 (0), 274 (−79.0), 257 (0), 247 (+29.4), 230 (0), 220 (−24.8), 212 (0); ^1^H NMR (acetone-*d*_6_, 500 MHz) δ: 4.12 (1H, t, *J* = 5.1, 11.6 Hz, glc-6a), 4.84 (1H, dd, *J* = 8.5, 11.6, glc-6b), 5.11 (1H, dd, *J* = 5.1, 8.5, glc-5), 5.48 (1H, d, *J* = 4.1, glc-3), 5.63 (1H, dd, *J* = 0.7, 4.1, glc-4), 5.71 (1H, d, *J* = 5.7, glc-2), 6.22 (1H, d, *J* = 5.7, glc-1), 6.99 (2H, s, galloyl-H), 7.16, 7.46, 7.86, 8.27 (each 1H, each s, aromatic) 7.93–8.01, 8.15–8.36 (each m, phenazine-H); ^13^C NMR (acetone-*d*_6_, 125 MHz) δ: 66.7, 68.2, 68.7, 76.2, 76.8 (glc-2~6), 91.7 (glc-1), 110.2 (galloyl-2,6), 113.5 (acyl-3), 115.7, 116.0, 116.6, 117.0, 118.5 (galloyl-1, acyl-1, 1**’**), 120.1, 120.2, 120.5, 122.7 (acyl-2, 3**’**), 130.2, 130.6, 130.7 (acyl-4**”**, 5**”**), 132.2, 132.4, 132.5 (acyl-3**”**, 6**”**), 136.1, 136.2 (acyl-2**’**), 138.2, 139.2, 139.5, 139.8, 139.9, 142.8, 142.9, 143.3, 143.5, 145.2, 145.3, 145.5, 145.7, 145.9 (galloyl-4, acyl-4, 5, 6, 4**’**, 5**’**, 1**”**, 2**”**), 146.2 (galloyl-3, 5), 152.3, 152.4 (acyl-6**’**), 164.9, 166.2, 166.6, 167.5, 167.9 (esters).

### 3.7. Treatment of **1** at pH 6

Amariin (**1**) (1.0 g) was dissolved in citrate-phosphate buffer (0.05 M, pH 6.0) and stirred at r.t. for 20 h. The mixture was acidified by addition of 5% TFA to pH 2 and applied to Sephadex LH-20 column chromatography (3 cm i.d. × 30 cm, 0%–100% MeOH in H_2_O, 20% stepwise, each 200 mL, followed by MeOH-acetone-H_2_O, 90:6:4, 80:12:8, 70:18:12, each 200 mL) to yield 11 fractions. Fr. 3 (23.5 mg) was identified as gallic acid. Fr. 4 (60.6 mg) was subjected to Chromatorex ODS column chromatography (2 cm i.d. × 17 cm, 0–70% MeOH in H_2_O, 5% stepwise, each 100 mL) to give 1-*O*-galloyl-3,6-(*S*)-HHDP-β-d-glucose (17.6 mg). Fr. 5 (43.7 mg) was purified by MCI gel CHP 20P column chromatography (2 cm i.d. × 17 cm, 0-50% MeOH in H_2_O, 5% stepwise, each 100 mL) to afford 1-*O*-galloyl-3,6-(*R*)-HHDP-β-d-glucose (corilagin, 24.0 mg). Similar separation of Fr. 7 (63.9 mg) yielded 1-*O*-galloyl-2,3-(*R*)-DHHDP-β-d-glucose (furosin, 34.4 mg). Fractions 9 and 10 were identified to be geraniin (**2**)(463.9 mg) and ellagic acid (48.0 mg), respectively.

*1-O-Galloyl-3,6-(S)-HHDP-β-d-glucose*: Brown amorphous powder; [α${]}_{D}^{29}$ +20.7° (*c* 0.10, MeOH); UV (MeOH) λ_max_ (log ε): 293 (4.36), 273 (4.49), 227 (4.80), 219 (4.88); IR ν_max_ cm**^−^**^1^: 3378, 1709, 16213; ESI-MS (negative) *m*/*z*: 633 [M − H]^−^; HR-FAB-MS *m*/*z*: 657.0705 (calcd for C_27_H_22_O_18_Na, 657.0703); ECD (MeOH) Δε (nm): +3.38 (229), −2.16 (267), +1.45 (293); ^1^H NMR (acetone-*d*_6_, 500 MHz) δ: 7.21 (2H, s, galloyl-H-2,6), 7.23 (1H, s, HHDP-H-3), 7.01 (1H, s, HHDP-H-3’), 6.29 (1H, d, *J* = 9.0 Hz, glc-1), 5.10 (1H, dt, *J* = 1.1, 5.7 Hz, glc-3), 4.88 (1H, d, *J* = 12.3 Hz, glc-6), 4.25 (1H, br d, *J* = 2.4 Hz, glc-5), 3.96 (1H, dt, *J* = 5.7, 9.0 Hz, glc-2), 3.84 (1H, dd, *J* = 3.4, 12.3 Hz, glc-6), 3.59 (1H, br s, glc-4); ^13^C NMR (acetone-*d*_6_, 125 MHz) δ: 168.0 (HHDP-7), 167.7 (HHDP-7’), 166.1 (galloyl-7), 145.8 (galloyl-3,5), 144.6, 144.5 (HHDP-4,4’,6,6’), 139.1 (galloyl-4), 138.1 (HHDP-5), 136.4 (HHDP-5’), 126.4 (HHDP-2, 2’), 121.2 (galloyl-1), 117.5 (HHDP-1), 116.7 (HHDP-1’), 112.4 (HHDP-3’), 110.3 (galloyl-2,6), 108.1 (HHDP-3’), 92.3 (glc-1), 81.2 (glc-3), 80.1 (glc-5), 72.7 (glc-4), 70.2 (glc-2), 63.3 (glc-6).

### 3.8. Heating of **2** with Pyridine in CH_3_CN

A solution of **2** (2.0 g) in 4% pyridine in CH_3_CN (50 mL) was heated at 80 ˚C for 90 min. After cooling, the solution was mixed with 2% aqueous TFA (25 mL) and concentrated. Resulting aqueous solution was applied to Sephadex LH-20 column chromatography (3 cm i.d. × 25 cm, 60%–100% MeOH in H_2_O, 10% stepwise, each 100 mL, followed by MeOH-acetone-H_2_O, 80:10:10, 200 mL) to give 7 fractions. Fr. 3 (123.8 mg) was mainly composed of 1-*O*-galloyl-3,6-(*R*)-HHDP-β-d-glucose (corilagin) and product **9**. Fr. 4 (78.7 mg) contained corilagin, **9**, and ellagic acid. Fr. 5 (473.5 mg) was separated by Chromatorex ODS (4 cm i.d. × 25 cm, 0%–55% MeOH in H_2_O, 5% stepwise, each 100 mL) to give **2** (290.6 mg) and a mixture of **9** and **11**, which were separated by Diaion HP20SS (3 cm i.d. × 17 cm, 10–60% MeOH in H_2_O, 5% stepwise, each 50 mL) followed by purification using Chromatorex ODS chromatography (3 cm i.d. × 28 cm) to yield **9** (23.2 mg) and **11** (39.3 mg). Fr. 6 was separated H_2_O soluble and insoluble parts, and the soluble fraction was purified by Avicel cellulose column chromatography (2 cm i.d. × 15 cm) with 2% AcOH to give 1-*O*-galoyl-2,3;4,6-bis-(*R*)-HHDP-β-d-glucose (**10**) (30.9 mg). The H_2_O insoluble precipitates were combined with Fr. 7 and subjected to size-exclusion column chromatography using Sephadex LH-20 (2 cm i.d. × 55 cm) with 7M urea:acetone (2:3, *v*/*v*, containing conc. HCl at 5 mL/L)[34] to give fractions containing oligomeric polyphenols and **8**. The removal of urea by Diaion HP20SS column chromatography afforded **8** (595.8 mg) and oligomeric polyphenols (188.3 mg).

*Pyridine adduct* (**9**): A tan amorphous powder, [α${]}_{D}^{17}$ −47.5 (c 0.1, MeOH); IR ν_max_ cm^−1^: 3383, 1730, 1616, 1344; UV (MeOH) λ_max_ (log ε): 349 (3.88), 280 (4.48), 217 (4.78). ESI-MS *m*/*z*: 996 [M + H]^+^, *m*/*z*: 995 [M − H]^−^; HRFABMS: *m*/*z* 996.1108 [M + H]^+^ (calcd for C_46_H_30_NO_25_, 996.1107); ^1^H NMR (acetone-*d*_6_, 500 MHz) δ: 9.53, 9.08 [each 1H, br d, *J* = 6.4 Hz, pyridine (py)-2,6], 8.62, 8.45 (each 1H, br t, J = 6.4 Hz, py-3,5), 7.37 [1H, s, benzofuran (BF)-3], 7.09 (2H, s, galloyl-2,6), 7.02 (1H, s, HHDP-3), 6.67 (1H, s, HHDP-3**’**), 6.39 (1H, br s, glc-3), 6.20 (1H, d, *J* = 3.1 Hz, glc-1), 5.39 (1H, br s, glc-2), 4.70 (1H, d, *J* = 3.8 Hz, glc-4), 4.41 (2H, m, glc-5, 6), 4.18 (1H, dd, *J* = 5.1, 10.6 Hz, glc-6); ^13^C NMR (acetone-*d*_6_, 125 MHz) δ: 168.3 (HHDP-7**’**), 166.7 (BF-7), 166.6 (HHDP-7), 165.2 (BF-7**’**), 165.1 (galloyl-7), 149.7, 149.2 (py-2,6), 147.9, 147.7, 147.5 (py-4, BF-4**’**,6**’**), 146.0 (galloyl-3,5), 145.9 (BF-6), 145.13, 145.06, 145.0, 144.6 (HHDP-4,4**’**,6,6**’**), 140.8 (BF-5**’**), 139.9 (galloyl-4), 137.3 (HHDP-5), 136.7 (HHDP-5**’**), 135.8 (BF-5), 134.6 (BF-2**’**), 129.8, 129.0 (py-3,5), 125.9 (BF-3**’**), 124.4, 123.8 (HHDP-2,2**’**), 119.6 (galloyl-1), 117.0 (BF-3), 116.7 (HHDP-1), 115.8 (BF-2), 115.7 (HHDP-1**’**), 115.1 (BF-1**’**), 114.3 (BF-1), 110.3 (galloyl-2,6), 109.5 (HHDP-3), 108.8 (HHDP-3**’**), 91.8 (glc-1), 74.7 (glc-5), 73.6 (glc-2), 69.3 (glc-4), 64.2 (glc-6), 62.1 (glc-3).

### 3.9. Methylation and Alkaline Degradation of Oligomeric Product

Oligomeric products (100 mg) in methanol (2 mL) was treated with etherial diazomethane for 2 days. The mixture was heated with 5% NaOH at 80 °C for 12 h, acidified with diluted HCl, and then partitioned with diethyl ether. The ether layer was concentrated and treated with diaomethane, and the products were separated by silica gel column chromatography (30%–100% acetone in hexane, 10% stepwise, each 50 mL) to yield methyl trimethoxybenzoate (5 mg), dimethyl (*R*)-hexamethoxydiphenate (2.0 mg) and dimethoxycarbonyl tetramethoxydibenzofuran (1.7 mg).

### 3.10. Acid Hydrolysis of DHHDP Esters

A portion (0.50 mL) of an acetone solution of **2** (10.0 mg/mL), 1-*O*-galloyl-2,4-(*R*)-DHHDP-β-d-glucopyranose (6.0 mg/mL), and 1-*O*-galloyl-3,6-(*R*)-HHDP-β-d-glucopyranose (6.0 mg/mL) was dispensed into 5 mL screw-capped vials, and acetone was removed in vacuo. To each vial, 0.50 mL of 2% H_2_SO_4_ was added and heated at 100 °C for 7 h. The reaction mixture was mixed with DMSO (4.5 mL) to dissolve ellagic acid and analyzed by HPLC (Figure S9). Yield of the products was estimated based on the peak area and calibration curve obtained by using standard compounds: 4.76 μmol of gallic acid and 10.09 μmol of ellagic acid was produced from 5.25 μmol of **2**. Similarly, 4.16 μmol of gallic acid and 3.05 μmol of ellagic acid was produced from 4.61 μmol of 1-*O*-galloyl-2,4-(*R*)-DHHDP-β-d-glucopyranose, and 4.84 μmol of gallic acid and 5.59 μmol of ellagic acid was produced from 4.72 μmol of 1-*O*-galloyl-3,6-(*R*)-HHDP-β-d-glucopyranose.

### 3.11. Acid Hydrolysis of **2**

Geraniin (**2**) (1.00 g, 1.05 mmol, dried over P_4_O_10_) was hydrolyzed with 2% H_2_SO_4_ (50 mL) under reflux for 2 days. Insoluble precipitates were collected by filtration and washed with MeOH to give ellagic acid (504.5 mg, 1.67 mmol). The filtrate was directly subjected to Diaion HP20SS column chromatography (3 cm i.d. × 25 cm, 0%–80% MeOH in H_2_O, 10% stepwise, each 100 mL) to yield gallic acid (206.2 mg, 1.21 mmol), flavogallonic acid (9.8 mg, 0.02 mmol), and ellagic acid (66.0 mg, 0.22 mol). Total isolation yield of ellagic acid was 1.89 mmol.

### 3.12. Computational Methods

A conformational search was performed using the Monte Carlo method and the MMFF94 force field with Spartan ′14 (Wavefunction, Irvine, CA, USA). The obtained low-energy conformers within 6 kcal/mol were optimized at the B3LYP/6-31G(d,p) level in acetone (PCM). The vibrational frequencies were also calculated at the same level to confirm their stability, and no imaginary frequencies were found. ^1^H NMR coupling constants of the low-energy conformers with Boltzmann populations greater than 1% were calculated at the B3LYP/6-31G(d,p)u + 1s (using only the Fermi contact term) level in acetone (PCM) and scaled by using the slope parameter 0.94 [35]. The calculated data for each conformer were averaged according to the Boltzmann distribution theory at 298 K based on their relative Gibbs free energies. All DFT calculations were performed using Gaussian 09 [36]. GaussView was used to draw the three-dimensional molecular structures [37].

## 4. Conclusions

The results presented and described in this paper strongly suggest that HHDP is produced by the reduction of DHHDP, at least in the case of 3,6-HHDP of geraniin (**2**) from amariin (**1**) and isoamariin (**3**). This may be the key mechanism in the ellagitannin biosynthesis, which otherwise remains unclear. The similarity to the reaction mechanism for the epigallocatechin B-B’ ring biphenyl bond formation during black tea production also supports our hypothesis. The difference in reactivity between 3,6- and 2,4-DHHDP is probably explained by tensile forces from glucose, which depend on the conformation of the pyranose ring. In our investigation on the production mechanism of HHDP from DHHDP, understanding the missing electron donor is the most critical issue. However, presently, we can only introduce the experimental results as described herein.

## Supplementary Materials

The following are available online. Figure S1. The leaves of Triadica sebifera used for the HPLC analysis, Figure S2. HPLC profiles of 60% CH_3_CN extracts of *Elaeocarpus sylvestris* var. *ellipticus* fresh leaves, Table S1 ^1^H NMR data for phenazine derivatives. Figure S3. HPLC profiles of aqueous solution of amariin (**1**) in pH 6 citrate-phosphate buffer, Figure S4. HPLC profiles of isoamariin (**3**) in pH 6 citrate-phosphate buffer, Figure S5. Structures of minor products produced by degradation of **1**, Figure S6. HMBC correlations and ECD spectra of 1-*O*-galloyl-3,6-(*S*)-HHDP-β-d-glucose. Figure S7. HPLC profile (A) and UV spectrum (B) of oligomeric polyphenol fraction obtained from **2**, Figure S8. ^13^C NMR spectrum of oligomeric polyphenol fraction measured in acetone-*d*_6_. Figure S9. HPLC profiles of acid hydrolysis of **2**, 1-*O*-galloyl-2,4-(*R*)-DHHDP-β-d-glucopyranose, and 1-*O*-galloyl-3,6-(*R*)-HHDP-β-d-glucopyranose. Figures S10–S31, 1D, 2D NMR spectra of compounds **1**, **1a**, **3**, **3a**, and **9**, and computational methods.
