# Supplementary material for: Production of Ellagitannin Hexahydroxydiphenoyl Ester by Spontaneous Reduction of Dehydrohexa-hydroxydiphenoyl Ester
Source: Molecules. 2020 Feb 26;25(5):1051. doi: 10.3390/molecules25051051 (PMC7179100; doi:10.3390/molecules25051051)
Supplement: Supplementary file 1 [file molecules-25-01051-s001.pdf]

Supplementary Materials

Production of Ellagitannin Hexahydroxydiphenoyl Ester by Spontaneous Reduction of Dehydrohexahydroxydiphenoyl Ester

Manami Era, Yosuke Matsuo, Yoshinori Saito, and Takashi Tanaka*

Table of Contents

Figure S1.	The leaves of *Triadica sebifera* (syn. *Sapium sebiferum*) used for the HPLC analysis.	S2
Figure S2.	HPLC profiles of 60% CH_3CN extracts of *Elaeocarpus sylvestris var. ellipticus* fresh leaves	S2
Table S1	^{1}H NMR data for phenazine derivatives.	S3
Figure S3.	HPLC profiles of aqueous solution of amariin (**1**) in pH 6 citrate-phosphate buffer at r.t.	S3
Figure S4.	HPLC profiles of isoamariin (**3**) in pH 6 citrate-phosphate buffer at r.t.	S4
Figure S5.	Structures of minor products produced by degradation of **1**.	S4
Figure S6.	HMBC correlations and ECD spectra of 1-*O*-galloyl-3,6-(*S*)-HHDP-β-D-glucose.	S4
Figure S7.	HPLC profile (A) and UV spectrum (B) of oligomeric polyphenol fraction obtained from **2**.	S5
Figure S8.	^{13}C NMR spectrum of oligomeric polyphenol fraction measured in acetone-d_6.	S5
Figure S9.	HPLC profiles of acid hydrolysis of 2, 1-*O*-galloyl-2,4-(*R*)-DHHDP-β-D-glucopyranose, and 1-*O*-galloyl-3,6-(*R*)-HHDP-β-D-glucopyranose.	S6
Figure S10.	^{1}H NMR spectrum of amariin (**1**) (500 MHz, in acetone-d_6)	S7
Figure S11.	^{13}C NMR spectrum of amariin (**1**) (125 MHz, in acetone-d_6)	S7
Figure S12.	^{1}H NMR spectrum of bisacetonyl amariin (**1a**) (500 MHz, in acetone-d_6)	S8
Figure S13.	^{13}C NMR spectrum of bisacetonyl amariin (**1a**) (125 MHz, in acetone-d_6)	S8
Figure S14.	^{1}H-^{1}H COSY spectrum of bisacetonyl amariin (**1a**) (in acetone-d_6)	S9
Figure S15.	NOESY spectrum of bisacetonyl amariin (**1a**) (in acetone-d_6)	S9
Figure S16.	HSQC spectrum of bisacetonyl amariin (**1a**) (in acetone-d_6)	S10
Figure S17.	HMBC spectrum of bisacetonyl amariin (**1a**) (in acetone-d_6)	S10
Figure S18.	^{1}H NMR spectrum and selected HMBC correlations for phenazine derivative of amariin.	S11
Figure S19.	^{1}H NMR spectrum of isoamariin (**3**) (500 MHz, in acetone-d_6).	S11
Figure S20.	^{13}C NMR spectrum of isoamariin (**3**) (125 MHz, in acetone-d_6).	S12
Figure S21.	^{1}H-^{1}H COSY spectrum of isoamariin (**3**) (500 MHz, in acetone-d_6).	S12
Figure S22.	NOESY spectrum of isoamariin (**3**) (500 MHz, in acetone-d_6).	S13
Figure S23.	HSQC spectrum of isoamariin (**3**) (in acetone-d_6).	S13
Figure S24.	HMBC spectrum of isoamariin (**3**) (in acetone-d_6).	S14
Figure S25.	^{1}H NMR spectrum of isoamariin phenazine derivative (**3a**) (400 MHz, in acetone-d_6).	S14
Figure S26.	^{13}C NMR spectrum of isoamariin phenazine derivative (**3a**) (100 MHz, in acetone-d_6).	S15
Figure S27.	^{1}H NMR spectrum of **9** in acetone-d_6 (500 MHz)	S15
Figure S28.	^{13}C NMR spectrum of **9** in acetone-d_6 (125 MHz)	S16
Figure S29.	^{1}H-^{1}H COSY spectrum of **9** in acetone-d_6 (500 MHz)	S16
Figure S30.	HSQC spectrum of **9** in acetone-d_6	S17
Figure S31.	HMBC spectrum of **9** in acetone-d_6	S17
Computational Methods.		S18

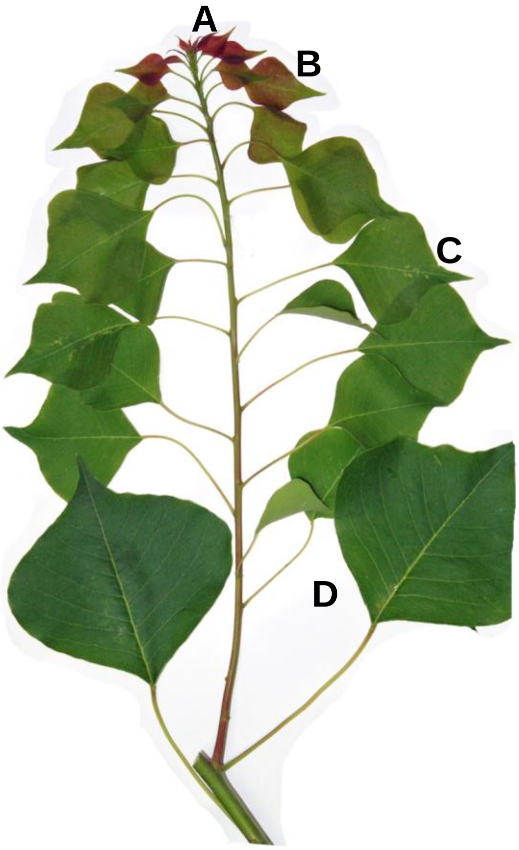

Figure S1. The leaves of *Triadica sebifera* (syn. *Sapium sebiferum*) used for the HPLC analysis.

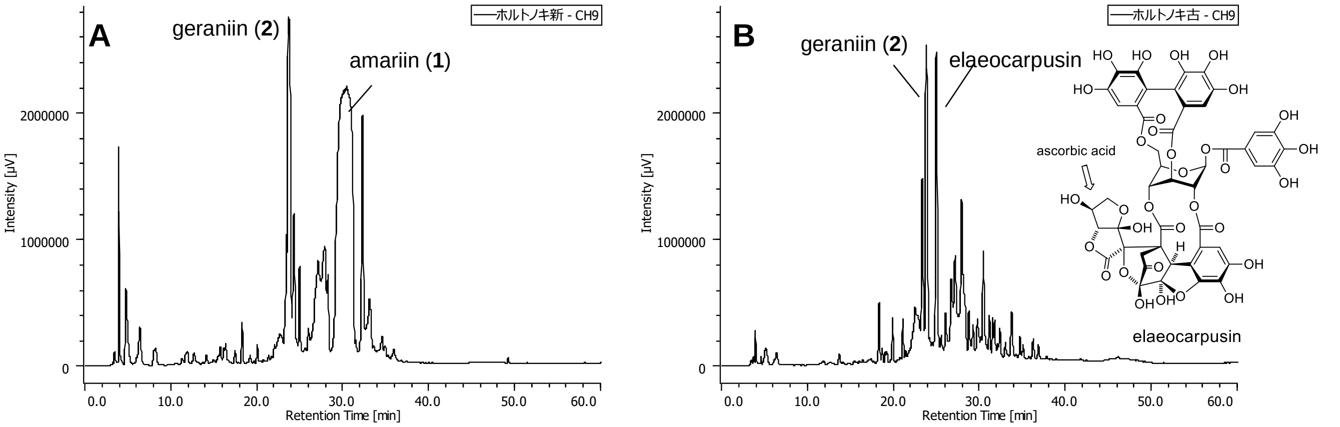

Figure S2. HPLC profiles of 60% CH_3CN extracts of *Elaeocarpus sylvestris var. ellipticus* fresh leaves collected in July (0.5g/7.5 mL, r.t.). **A**: young soft leaf at the top of twig, **B**: matured hard leaf at lower part of the same twig. Elaeocarpusin is an ascorbic acid adduct of geraniin. This compound easily decompose to give geraniin and ascorbic acid.[8]

Table S1. ^{1}H NMR data for phenazine derivatives in acetone-d_6 (δ in ppm, J in Hz).

	**isoamariin-phen**[a,c]			**didehydrogeraniin-phen**[b,c]			**amariin-phen**[a,c]		
Position	δ_H (J in Hz)			δ_H (J in Hz)			δ_H (J in Hz)		
1	6.22	d	(5.7)	6.29	d	(5)	6.22	d	(5.5)
**2**	**5.71**	**d**	**(5.7)**	**5.90**	**d**	**(5)**	**5.85**	**dd**	**(1.0, 5.5)**
3	5.48	d	(4.1)	5.44	d	(1)	5.39	d	(3.9)
**4**	**5.63**	**dd**	**(0.7, 4.1)**	**5.83**	**d**	**(4)**	**5.79**	**br. d**	**(3.9)**
5	5.11	dd	(5.1, 8.5)	4.80-5.16	m		4.94-5.04	m	
6a	4.84	dd	(8.5, 11.6)						
6b	4.12	t	(5.1, 11.6)	4.17	d	(9)	4.11	dd	(3.3, 11.8)
aromatic[d]	8.27, 7.86, 7.46, 7.16			8.37, 8.29, 7.57, 7.05			8.31, 8.23, 7.51, 7.02		

[a] 400 MHz, [b] ref 6 (measured in 200 MHz), [c] Phenazine derivatives were represented by amariin-phen, isoamariin-phen, and didehydrogeraniin-phen, [d] All aromatic signals are singlet.

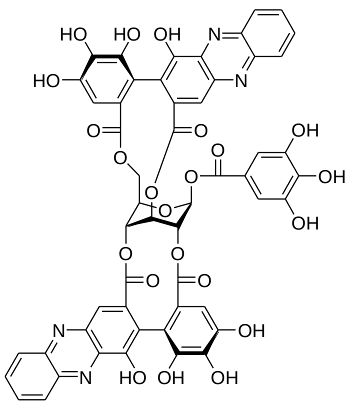

amariin phenazine derivative (**1b**)
(didehydrogeraniin phenazine derivative)

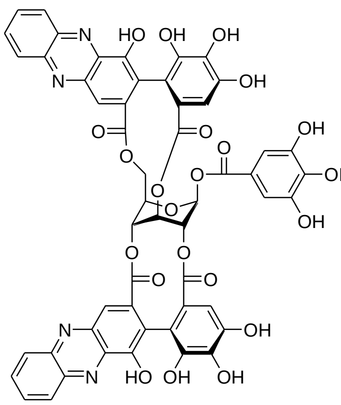

isoamariin phenazine derivative (**3a**)

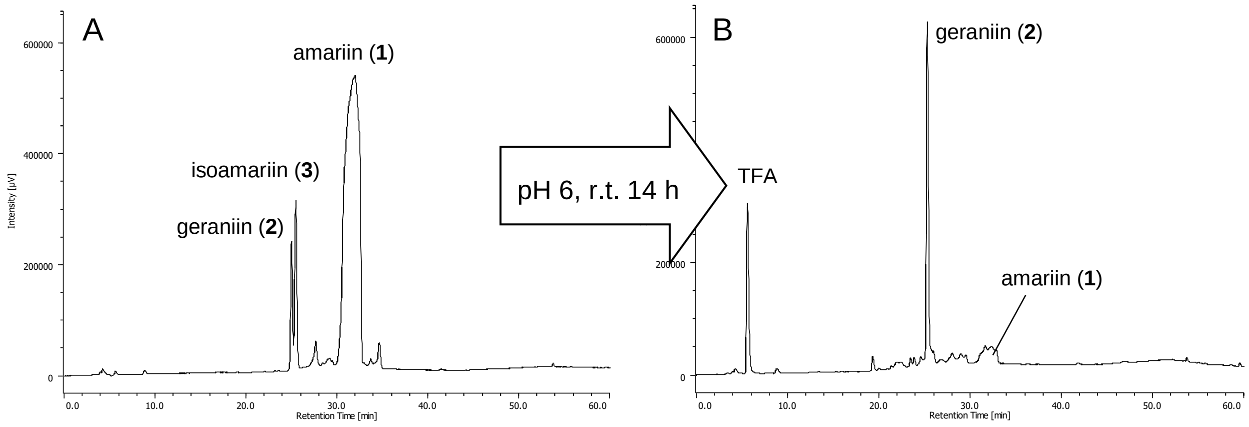

Figure S3. HPLC profiles of aqueous solution of amariin (**1**) in pH 6 citrate-phosphate buffer at r.t. **A**: 0 h, **B**: after 14 h. TFA was added to acidify the reaction mixture.

.

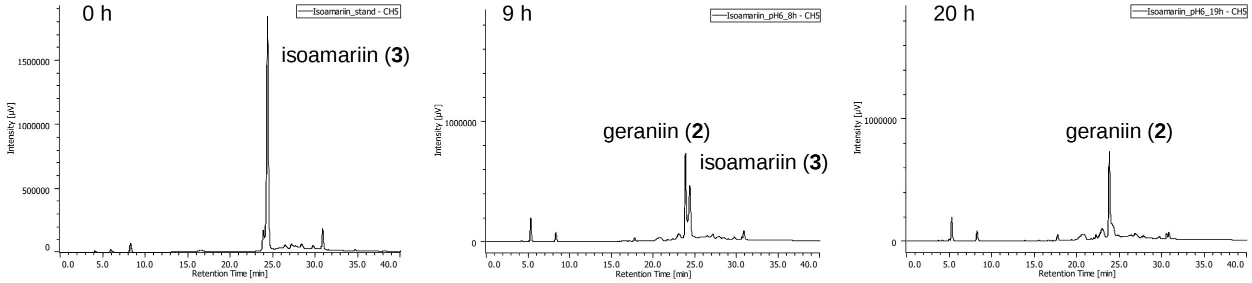

Figure S4. HPLC profiles of isoamariin (**3**) in pH 6 citrate-phosphate buffer at r.t.

Isoamariin (**3**) (5 mg) was dissolved in pH 6 citrate-phosphate buffer (0.05M, 1.0 mL) and kept at r.t. and analyzed by HPLC

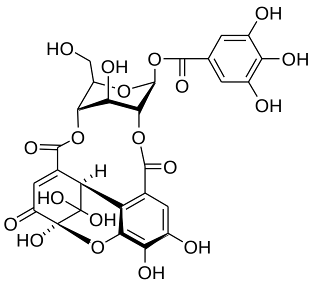

furosin

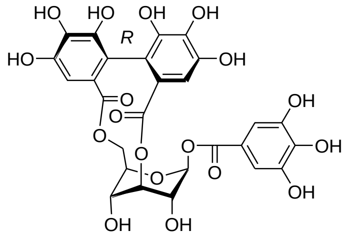

corilagin

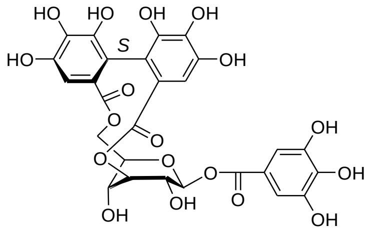

1-O-galloyl-3,6-(S)-HHDP–β-D-glucose

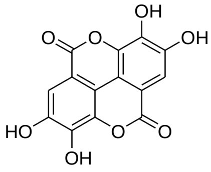

ellagic acid

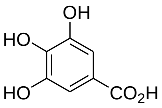

gallic acid

Figure S5. Structures of minor products produced by degradation of **1**.

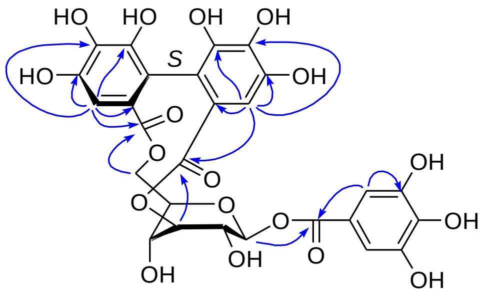

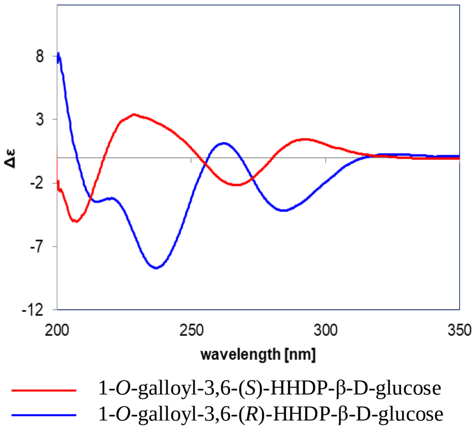

Figure S6. HMBC correlations and ECD spectra of 1-*O*-galloyl-3,6-(*S*)-HHDP-β-D-glucose.

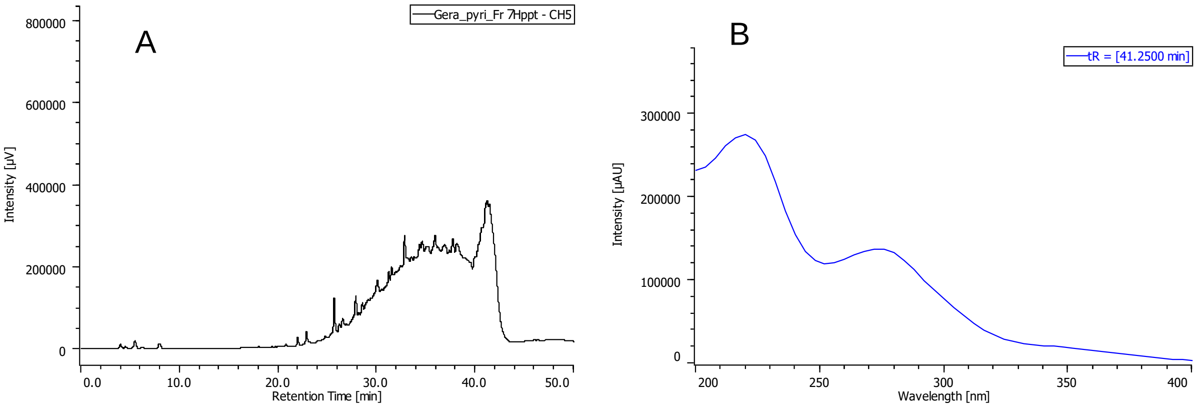

Figure S7. HPLC profile (A) and UV spectrum (B) of oligomeric polyphenol fraction obtained from **2**.

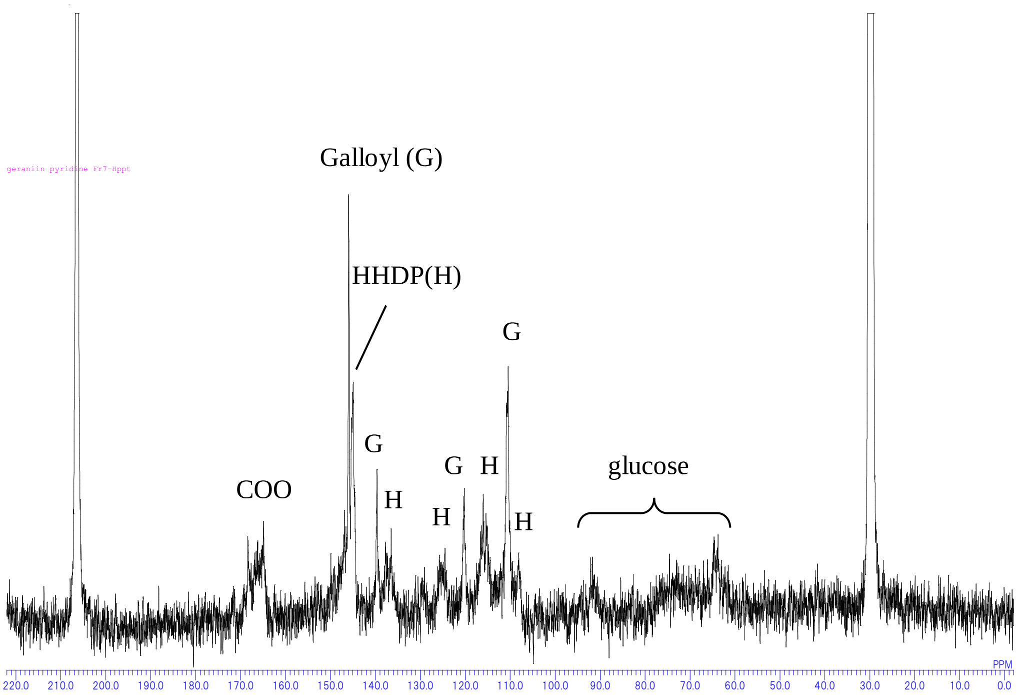

Figure S8. ^{13}C NMR spectrum of oligomeric polyphenol fraction measured in acetone-d_6 at 100 MHz.

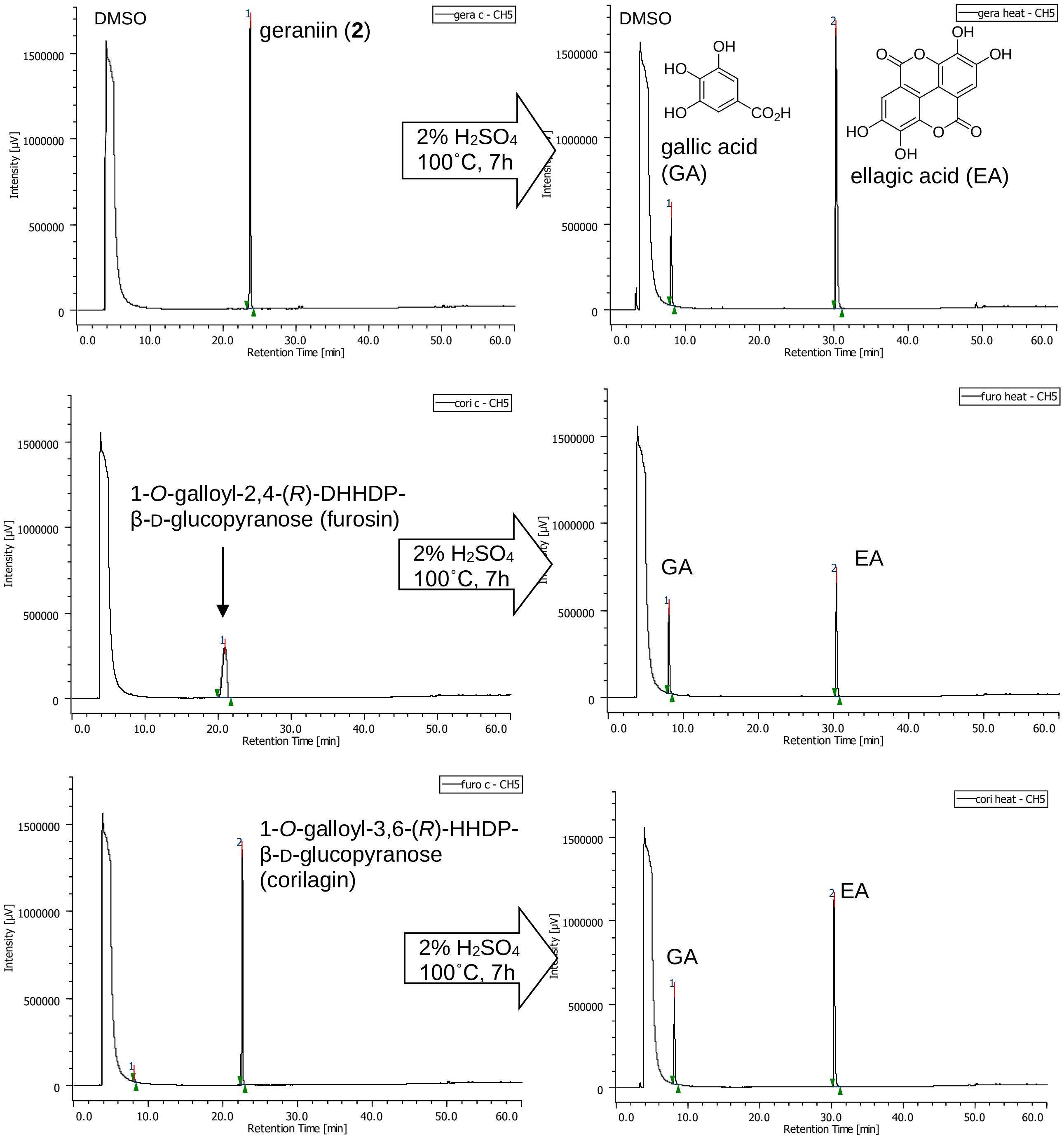

Figure S9. HPLC profiles of acid hydrolysis of **2**, 1-*O*-galloyl-2,4-(*R*)-DHHDP-β-D-glucopyranose, and 1-*O*-galloyl-3,6-(*R*)-HHDP-β-D-glucopyranose.

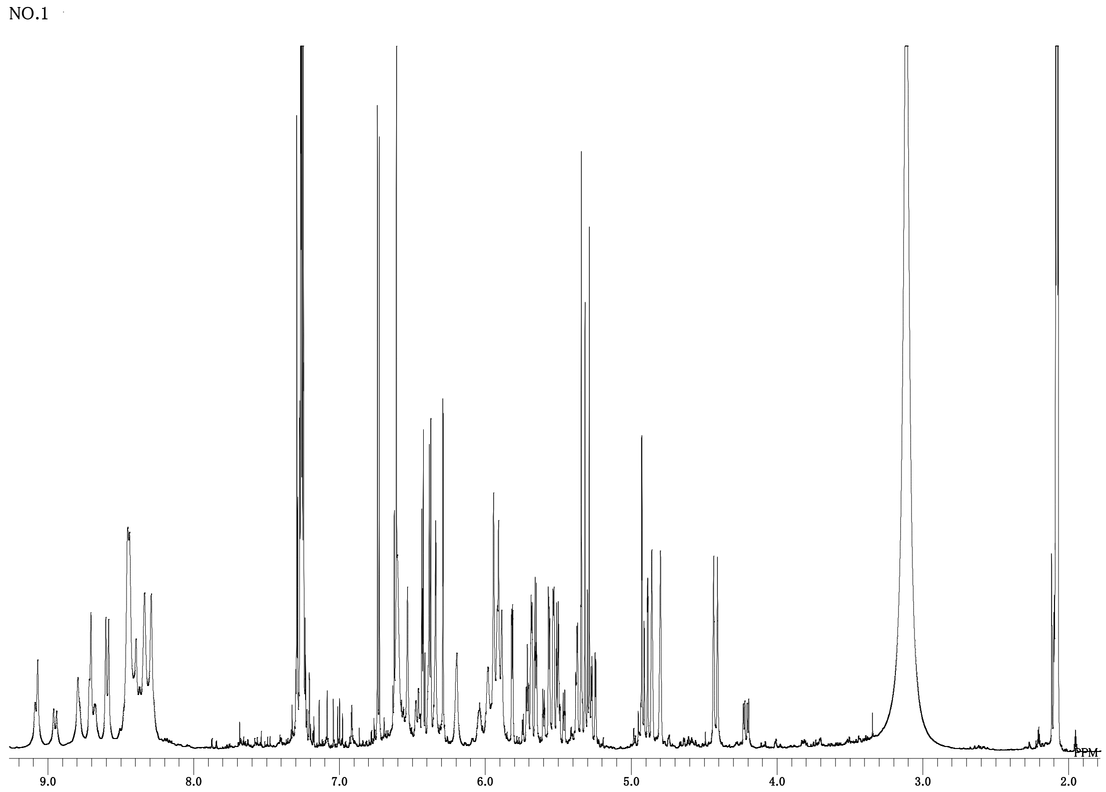

Figure S10. ^{1}H NMR spectrum of amariin (**1**) (500 MHz, in acetone-d_6)

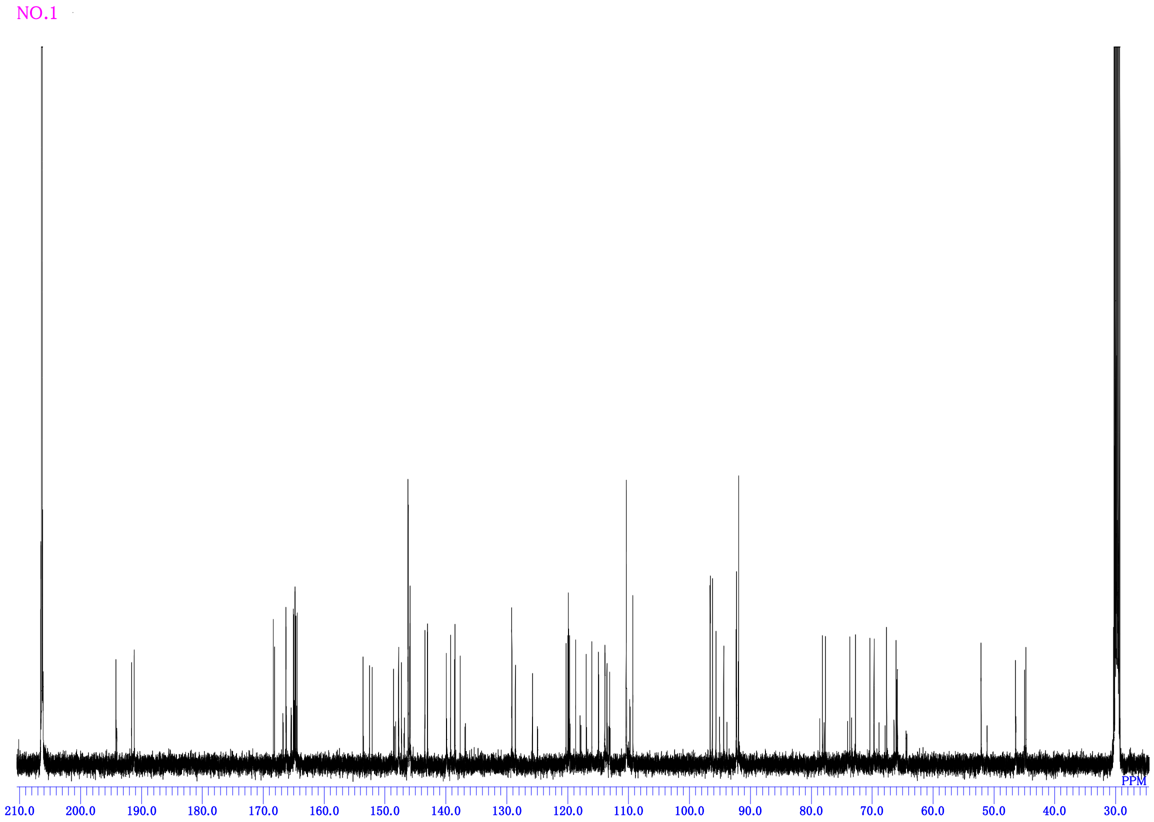

Figure S11. ^{13}C NMR spectrum of amariin (**1**) (125 MHz, in acetone-d_6)

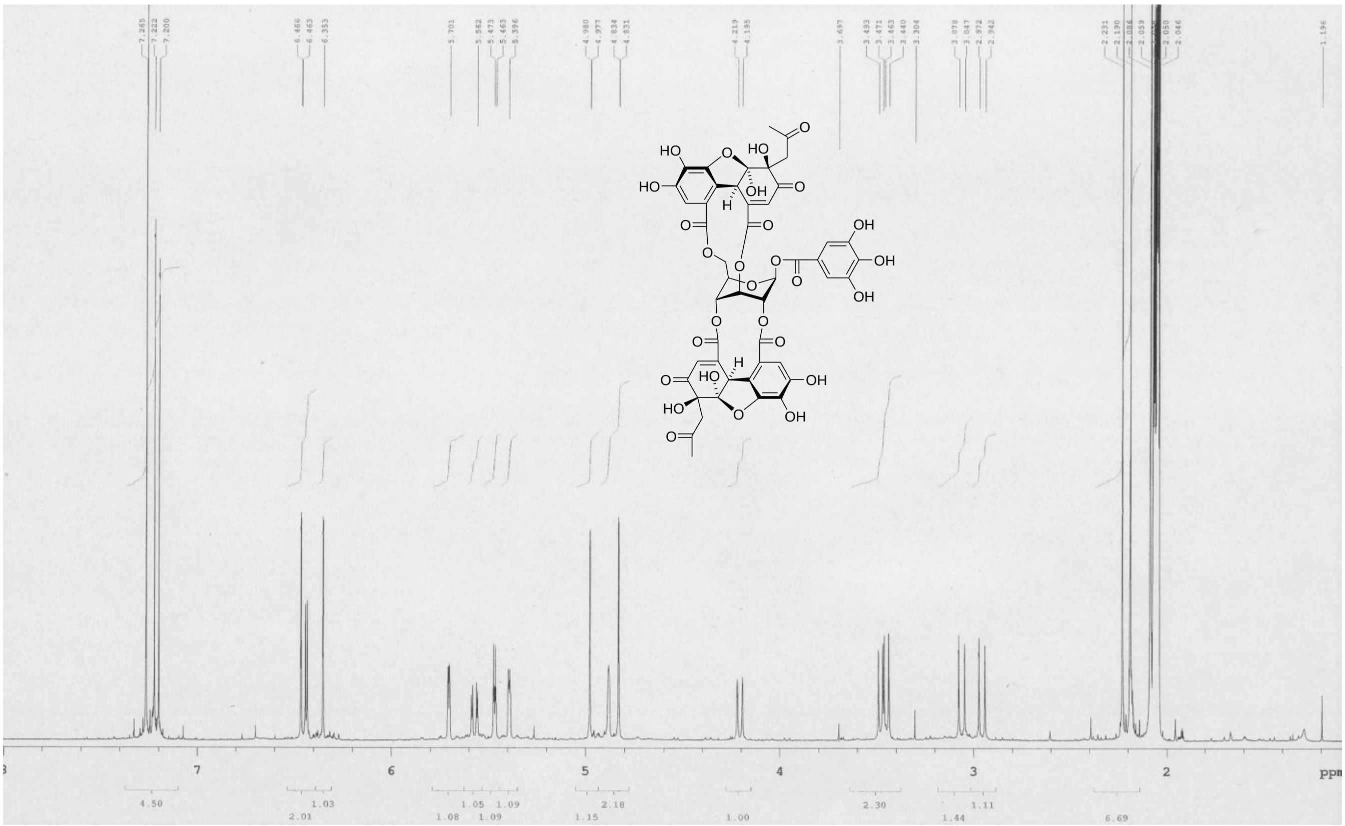

Figure S12. ^{1}H NMR spectrum of bisacetonyl amariin (**1a**) (500 MHz, in acetone-d_6)

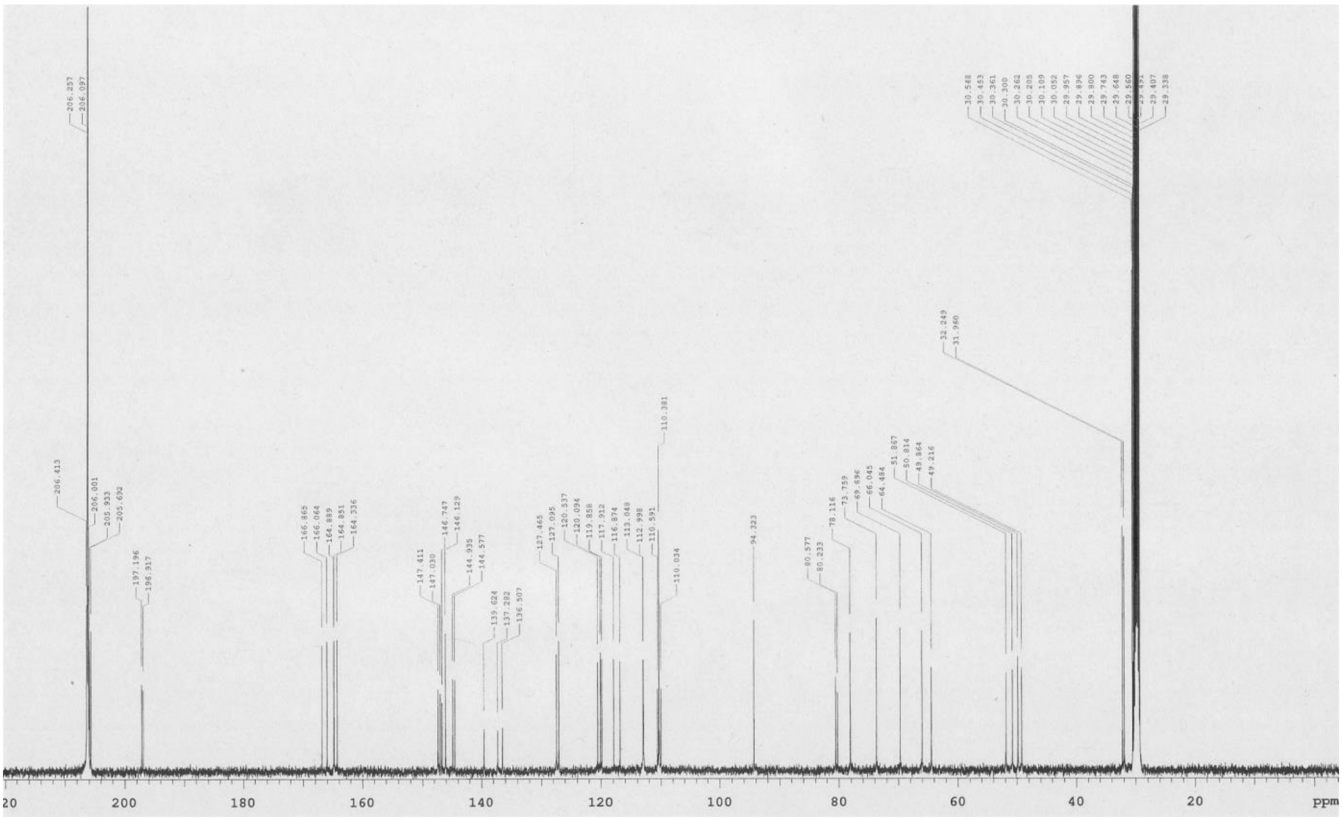

Figure S13. ^{13}C NMR spectrum of bisacetonyl amariin (**1a**) (125 MHz, in acetone-d_6)

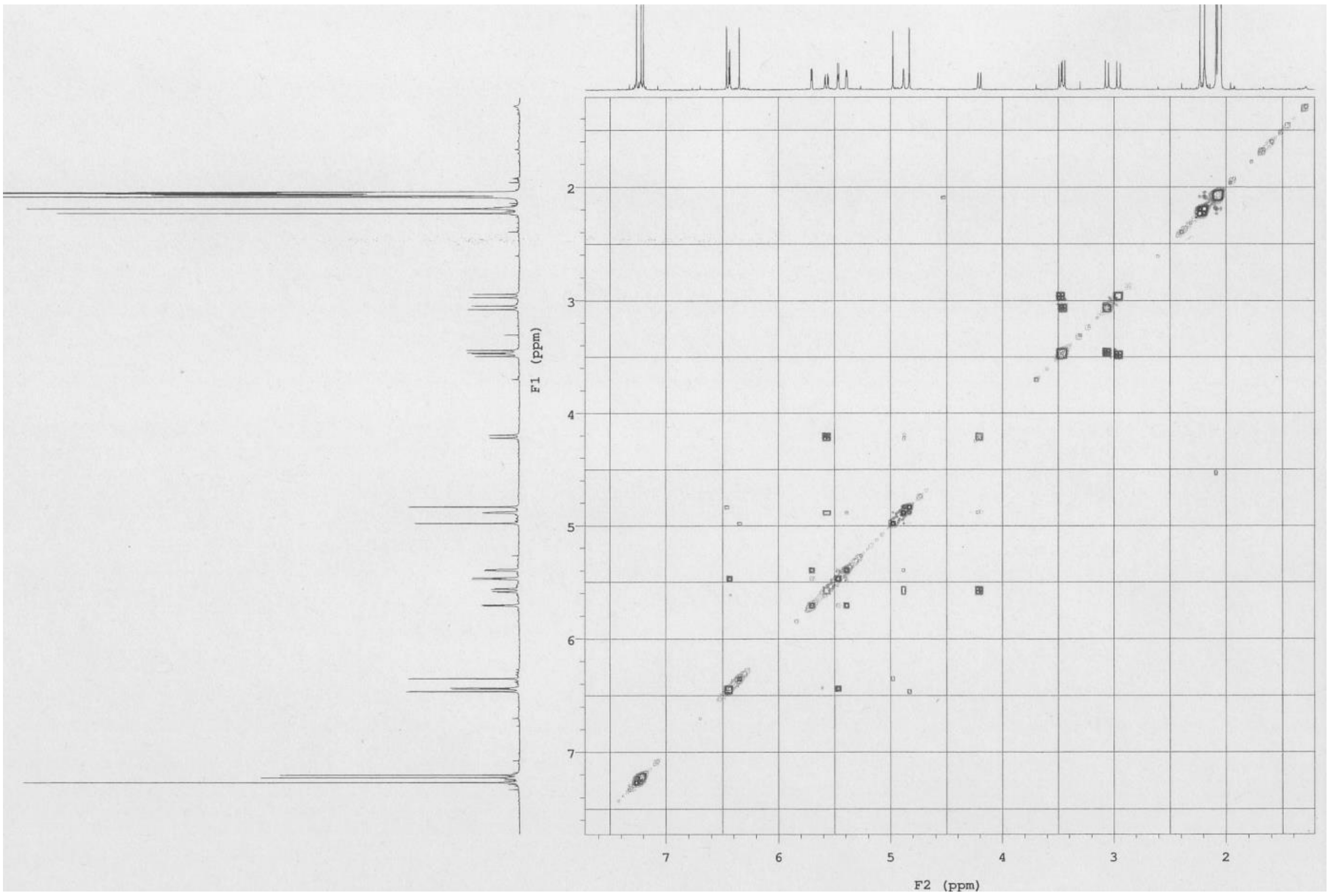

Figure S14. ^1H-^1H COSY spectrum of bisacetonyl amariin (**1a**) (in acetone-d_6)

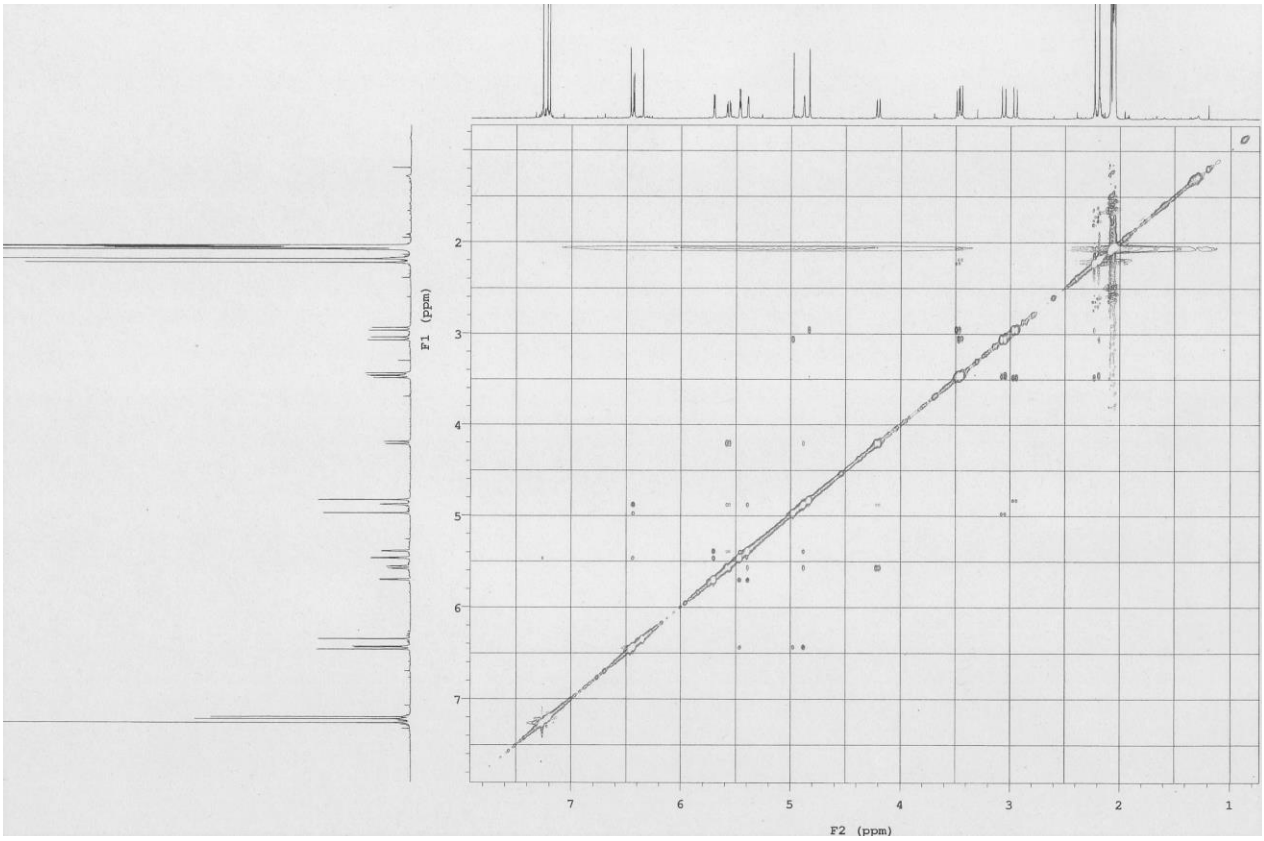

Figure S15. NOESY spectrum of bisacetonyl amariin (**1a**) (in acetone-d_6)

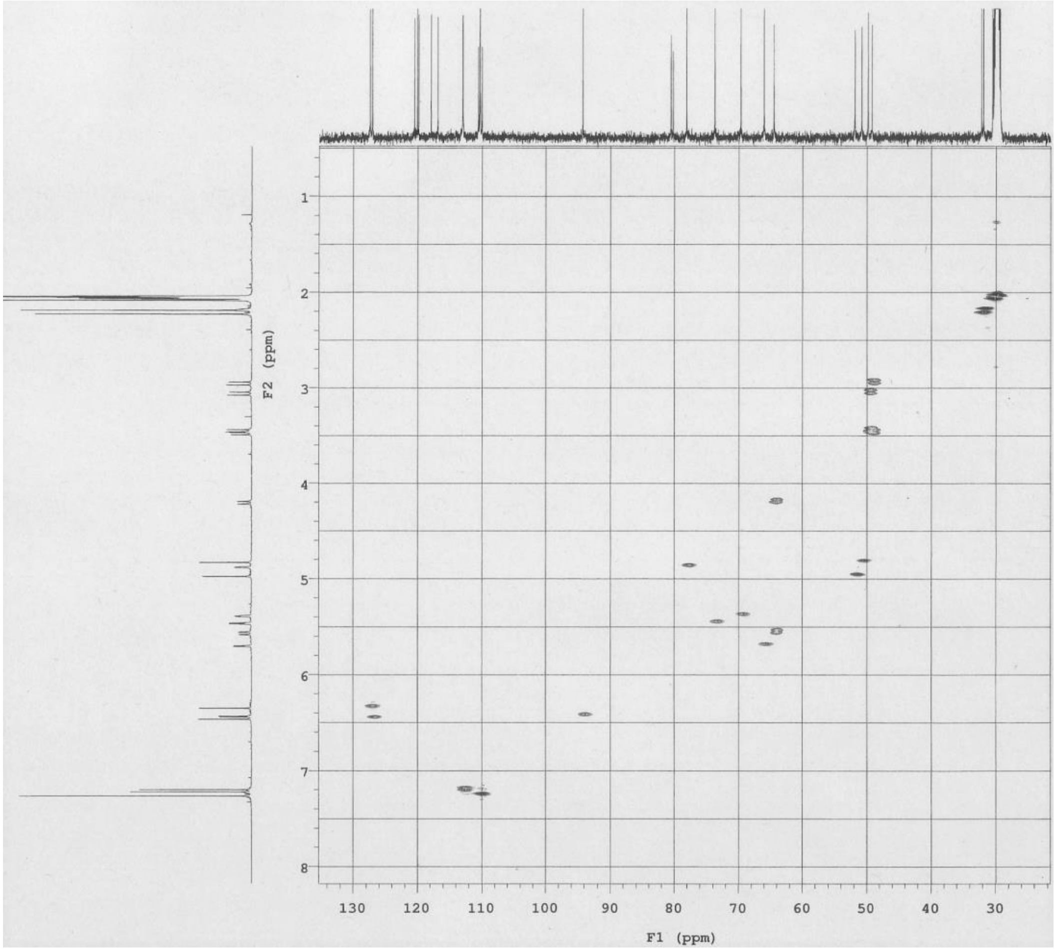

Figure S16. HSQC spectrum of bisacetonyl amariin (**1a**) (in acetone-d_6)

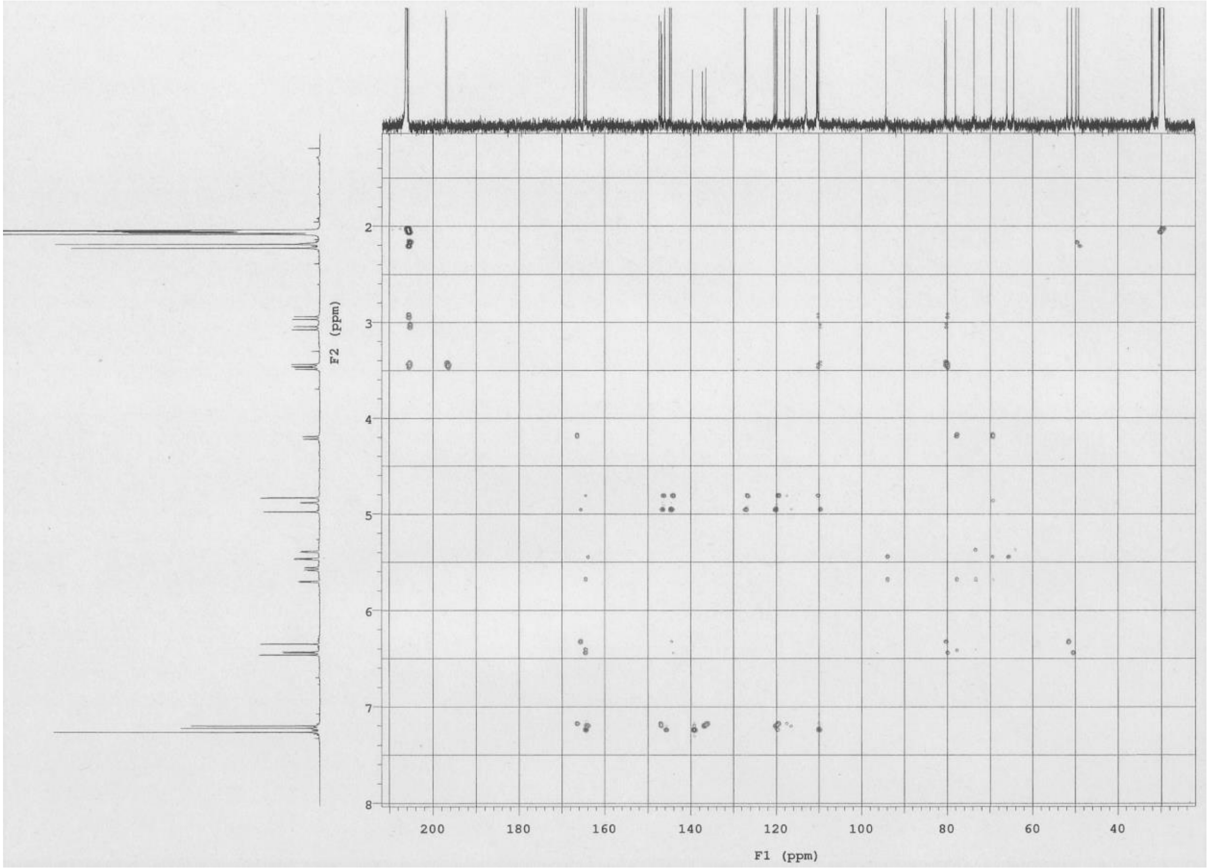

Figure S17. HMBC spectrum of bisacetonyl amariin (**1a**) (in acetone-d_6)

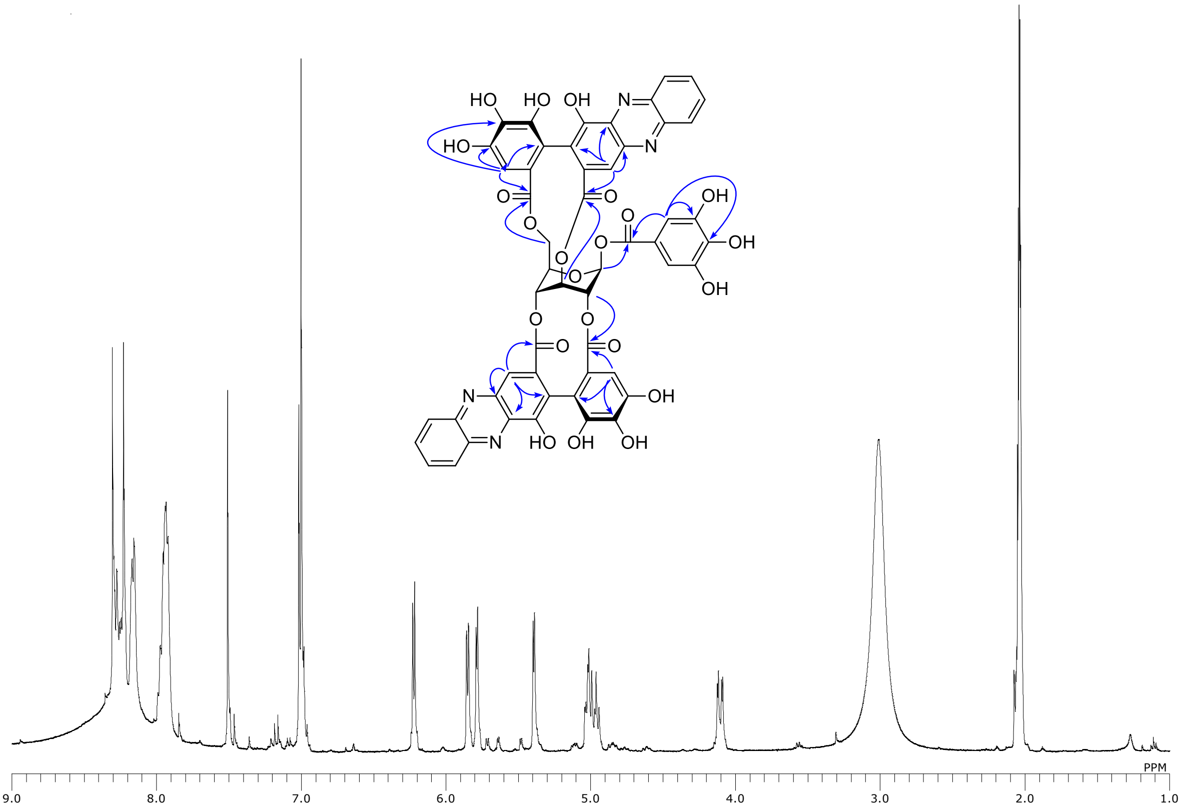

Figure S18. ^{1}H NMR spectrum and selected HMBC correlations for phenazine derivative of amariin.

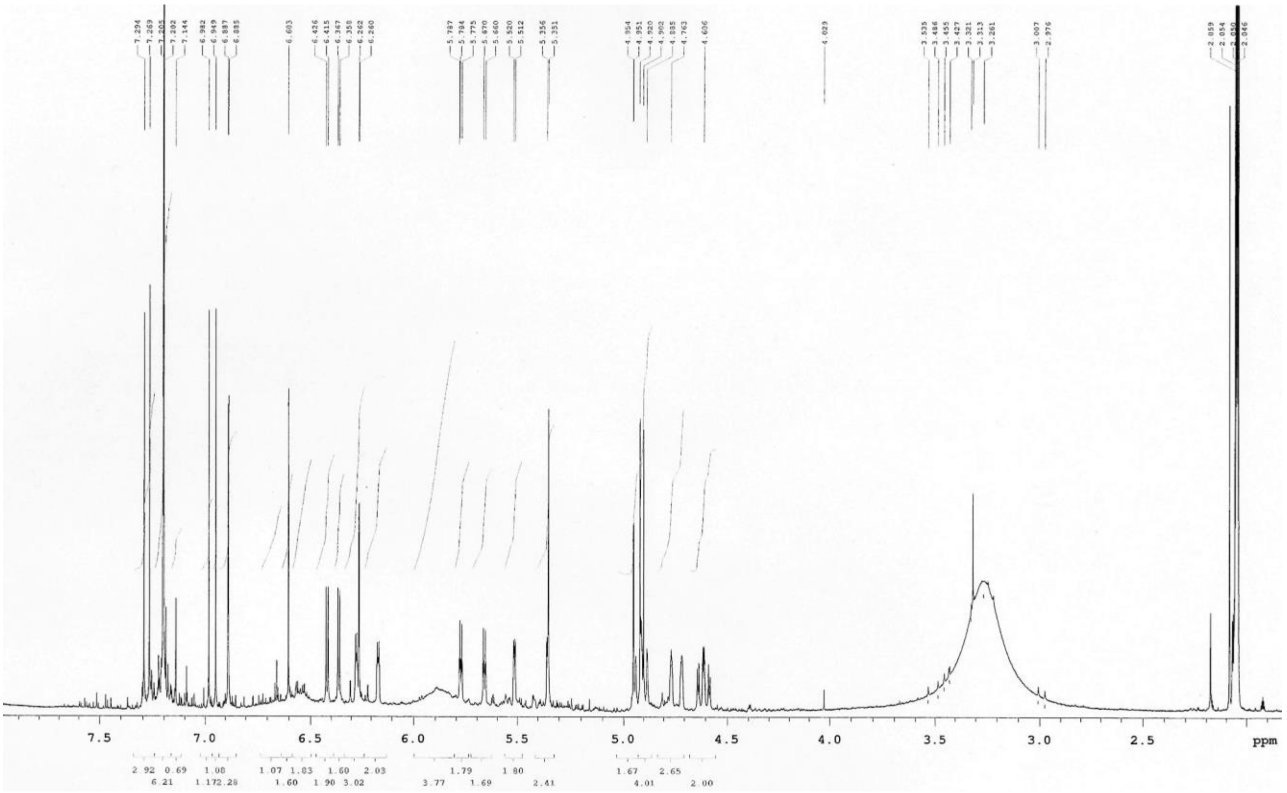

Figure S19. ^{1}H NMR spectrum of isoamariin (**3**) (500 MHz, in acetone-d_6).

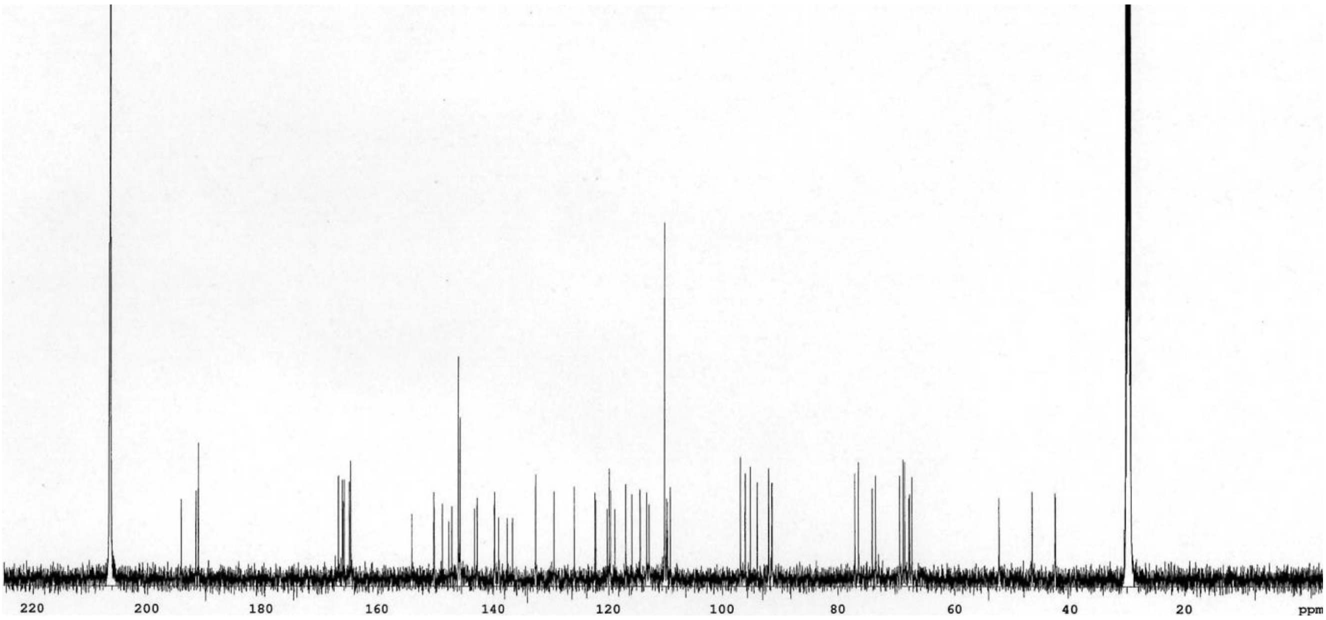

Figure S20. ^{13}C NMR spectrum of isoamariin (**3**) (125 MHz, in acetone-d_6).

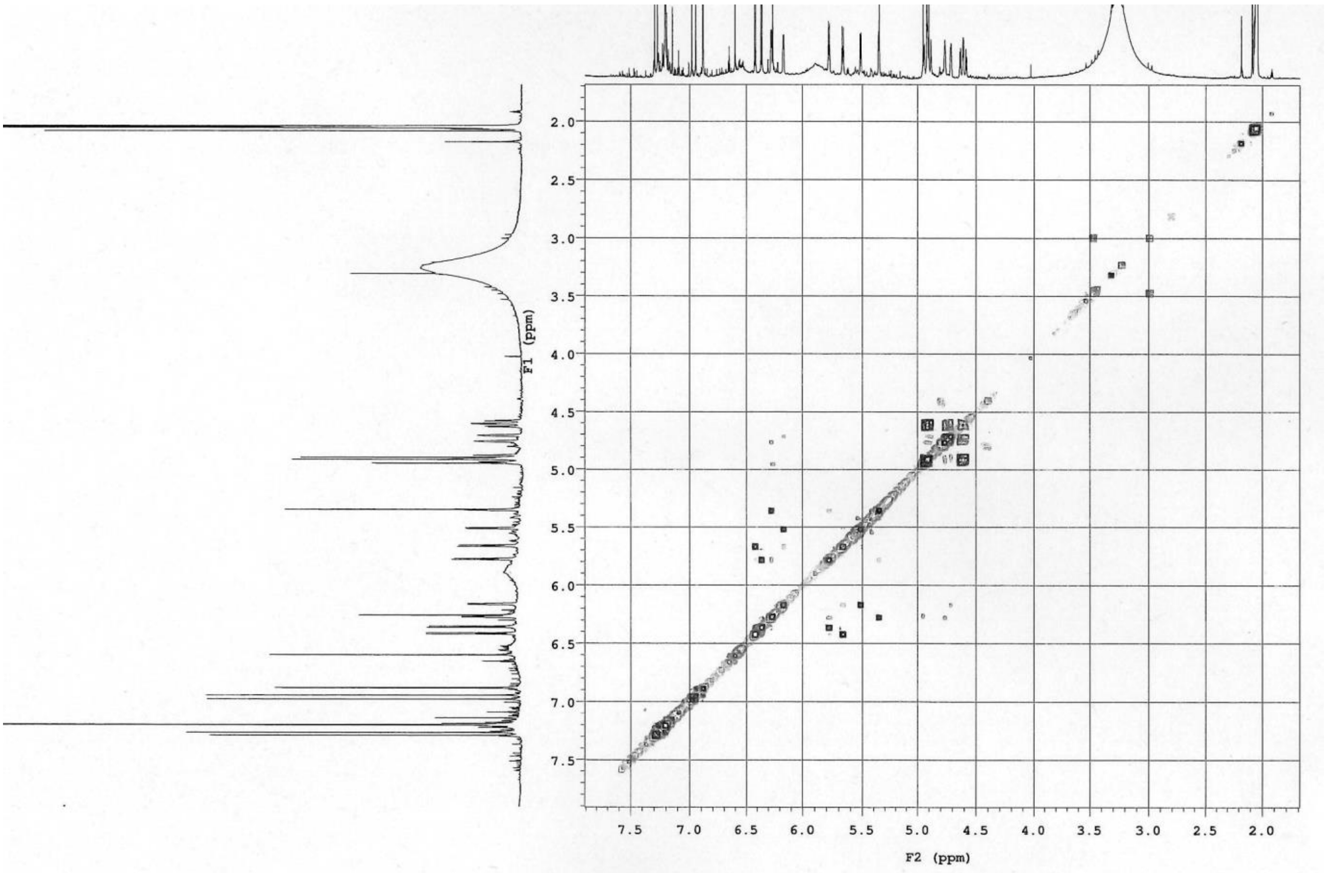

Figure S21. ^{1}H-^{1}H COSY spectrum of isoamariin (**3**) (500 MHz, in acetone-d_6).

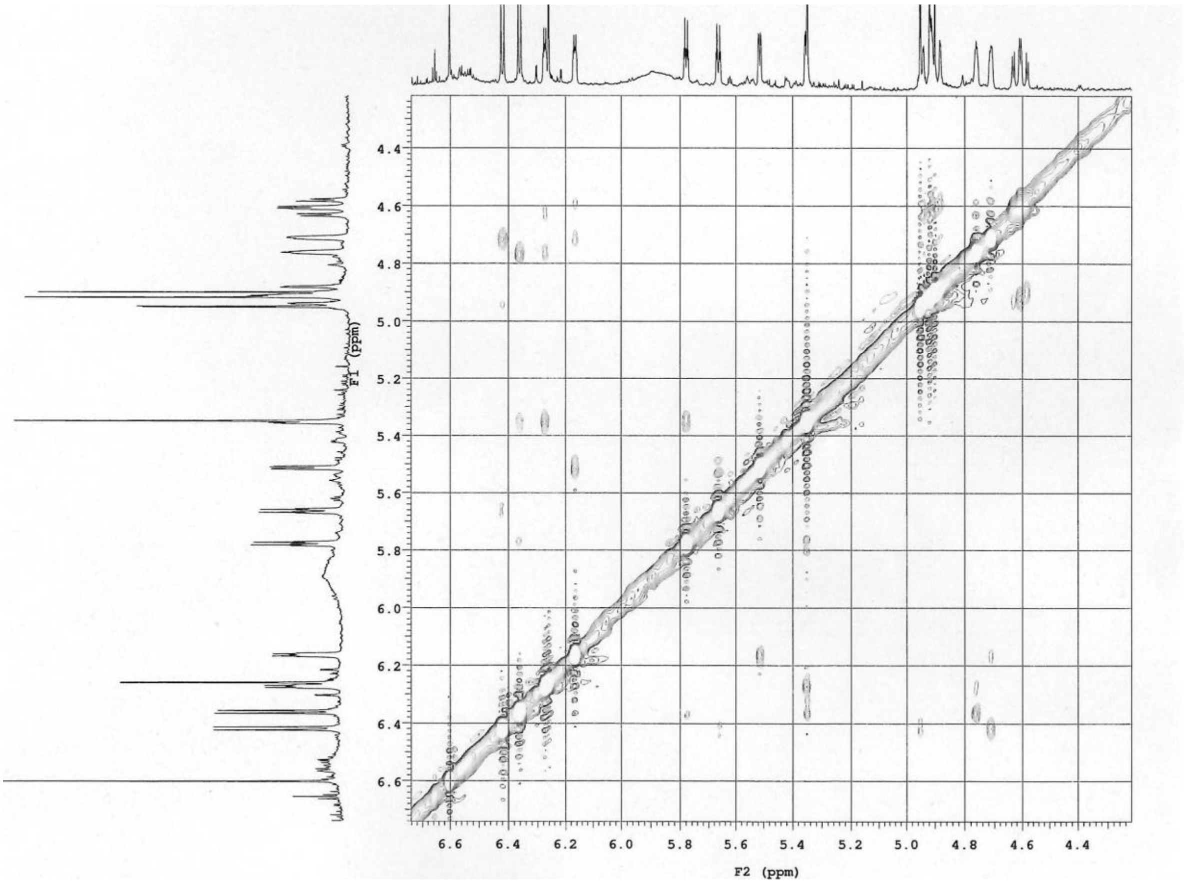

Figure S22. NOESY spectrum of isoamariin (**3**) (500 MHz, in acetone-d_6).

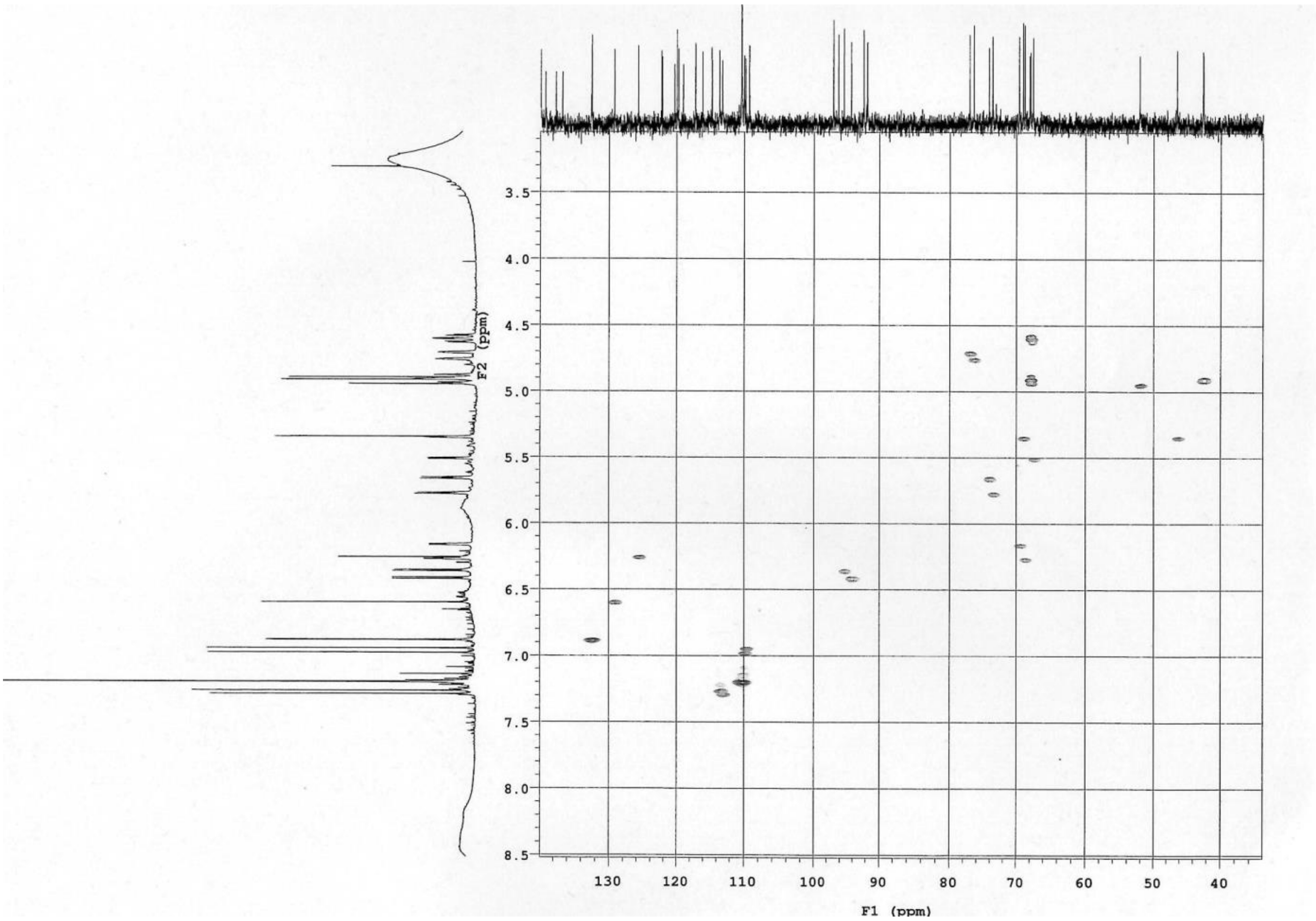

Figure S23. HSQC spectrum of isoamariin (**3**) (in acetone-d_6).

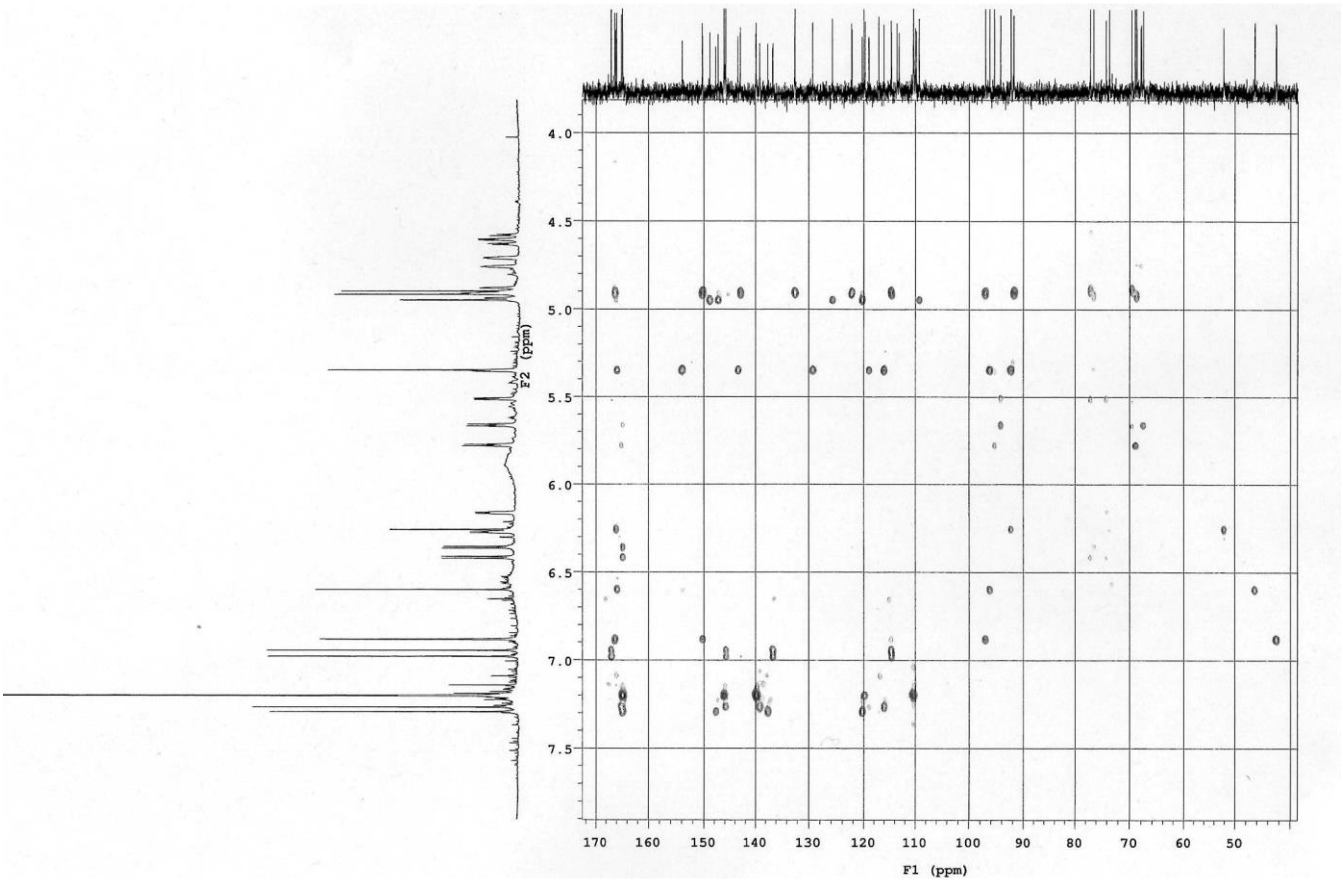

Figure S24. HMBC spectrum of isoamariin (**3**) (in acetone-d_6).

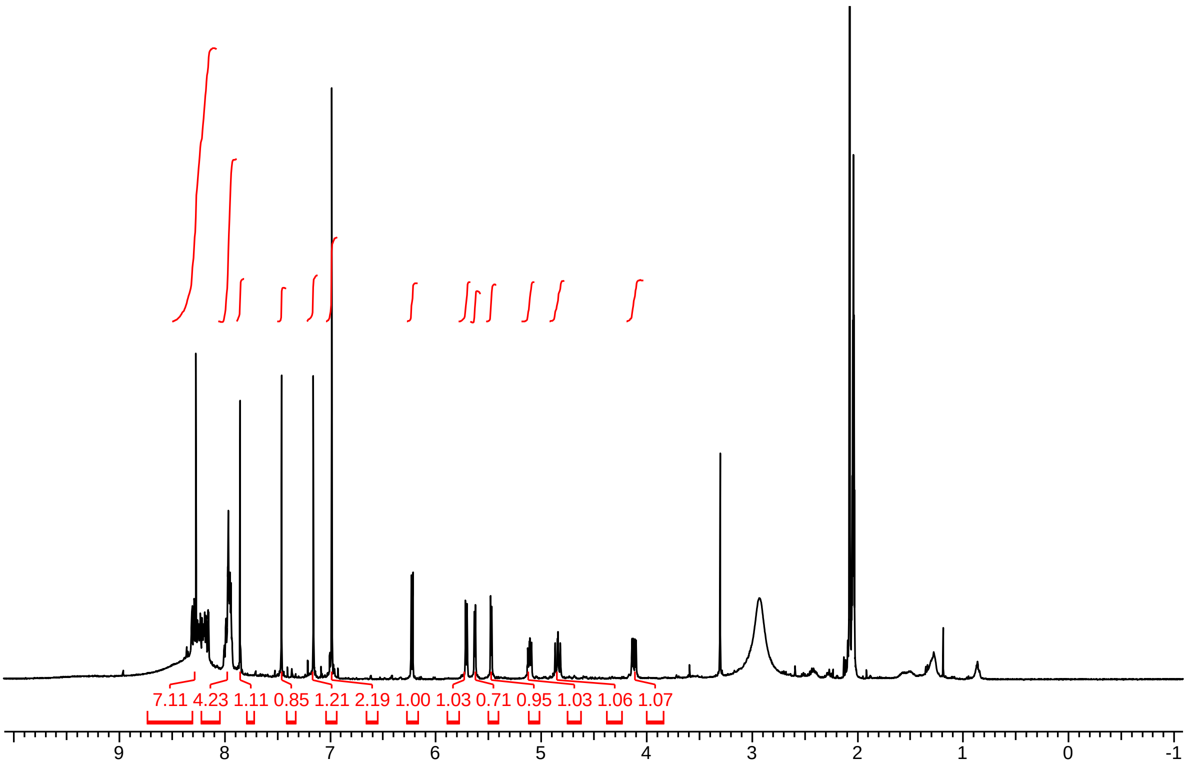

Figure S25. ^{1}H NMR spectrum of isoamariin phenazine derivative (**3a**) (400 MHz, in acetone-d_6).

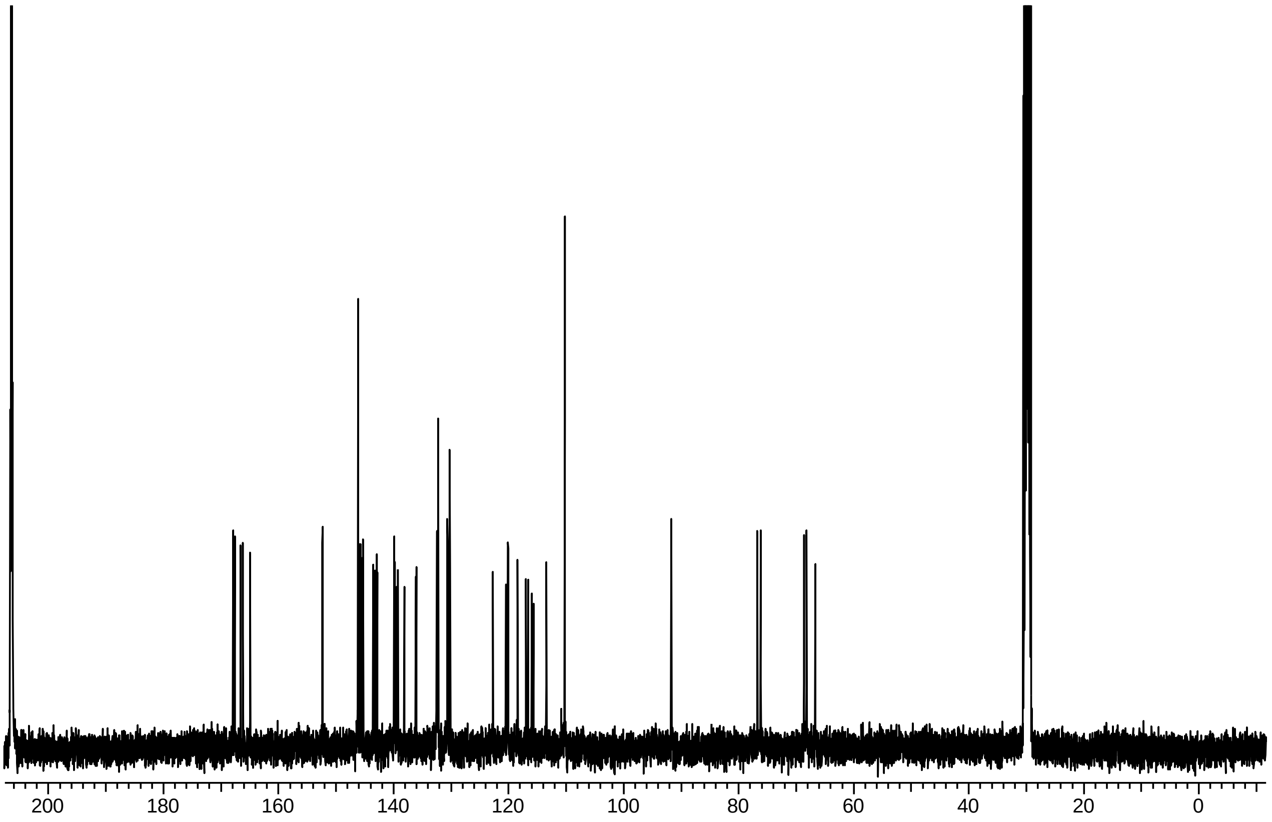

Figure S26. ^{13}C NMR spectrum of isoamariin phenazine derivative (**3a**) (100 MHz, in acetone-d_6).

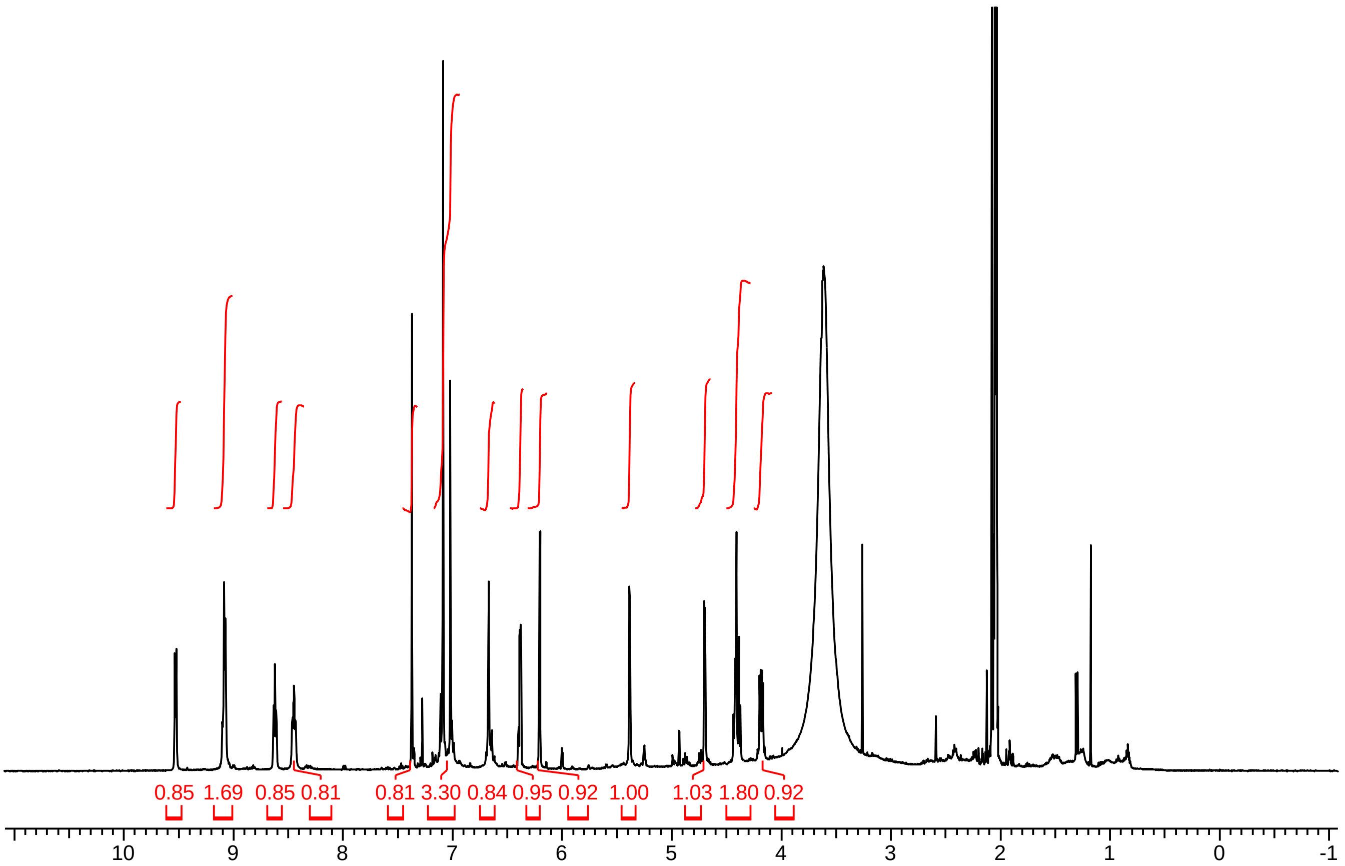

Figure S27. ^{1}H NMR spectrum of **9** in acetone-d_6 (500 MHz)

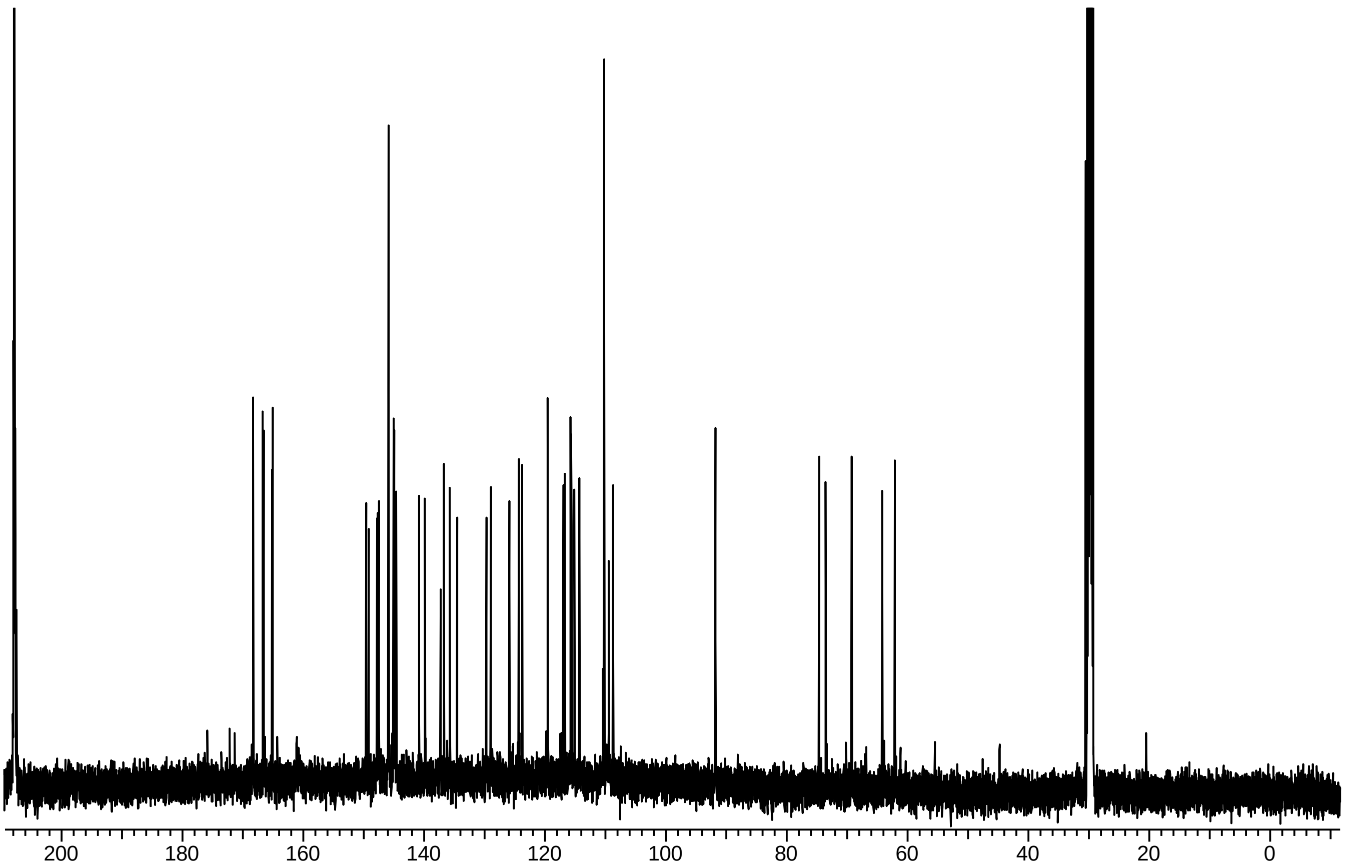

Figure S28. ^{13}C NMR spectrum of **9** in acetone-d_6 (125 MHz)

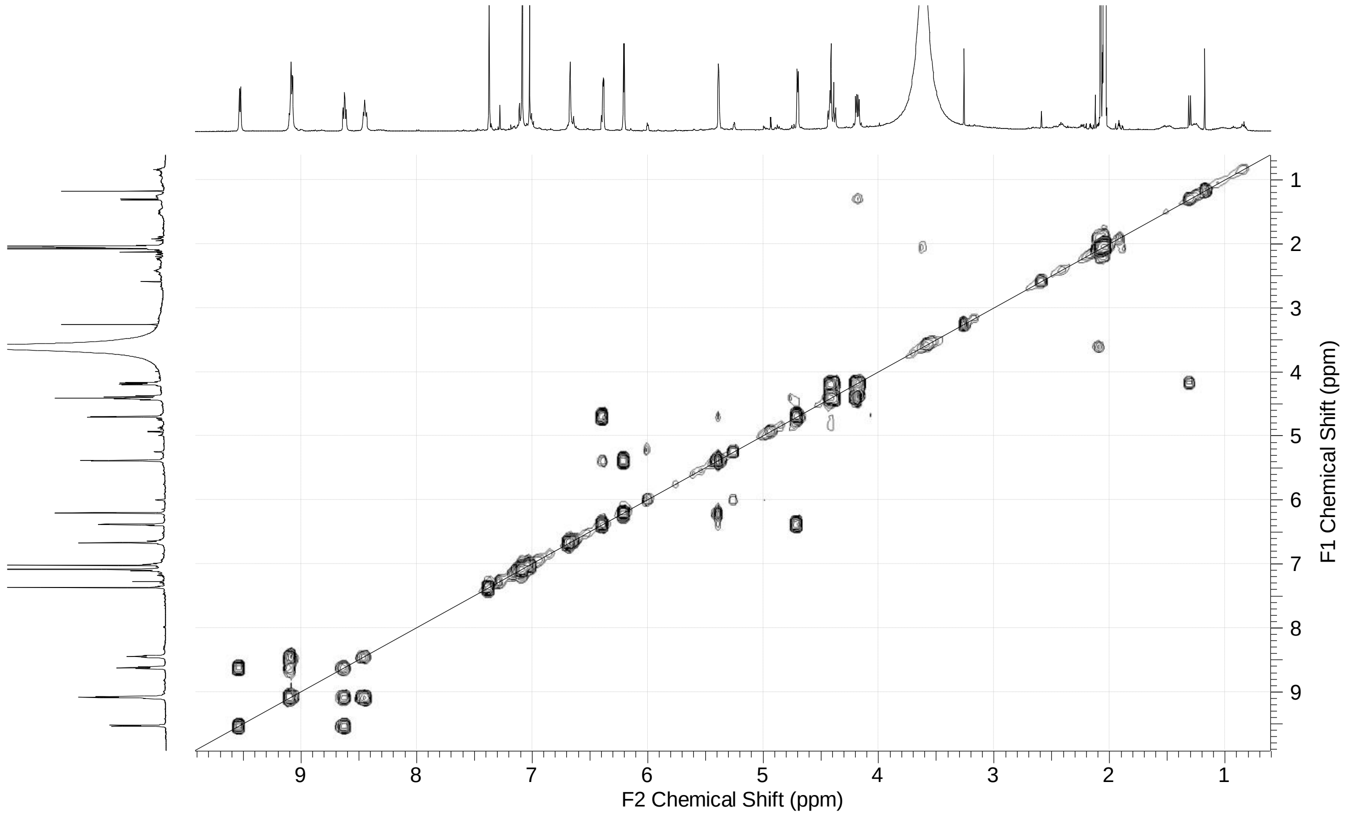

Figure S29. ^{1}H-^{1}H COSY spectrum of **9** in acetone-d_6 (500 MHz)

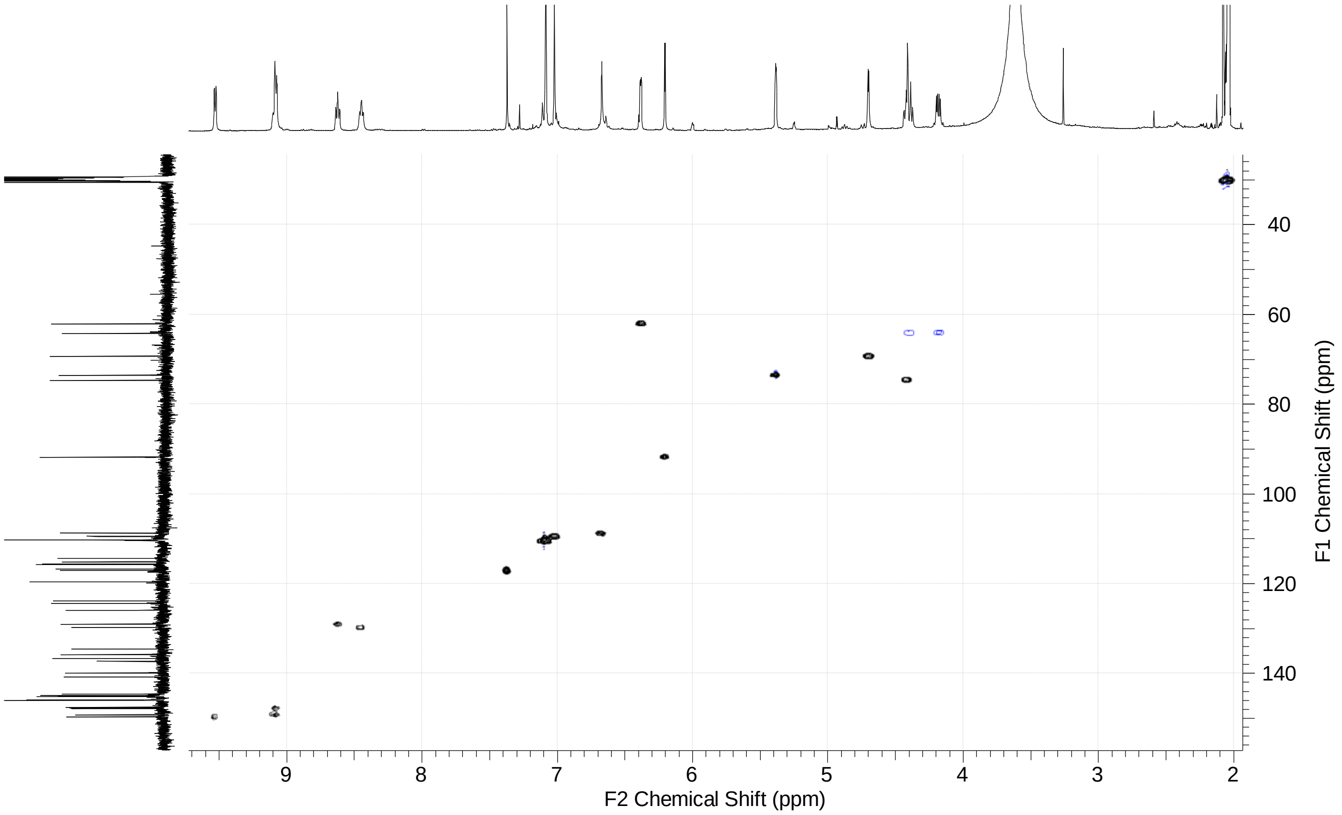

Figure S30. HSQC spectrum of **9** in acetone-d_6

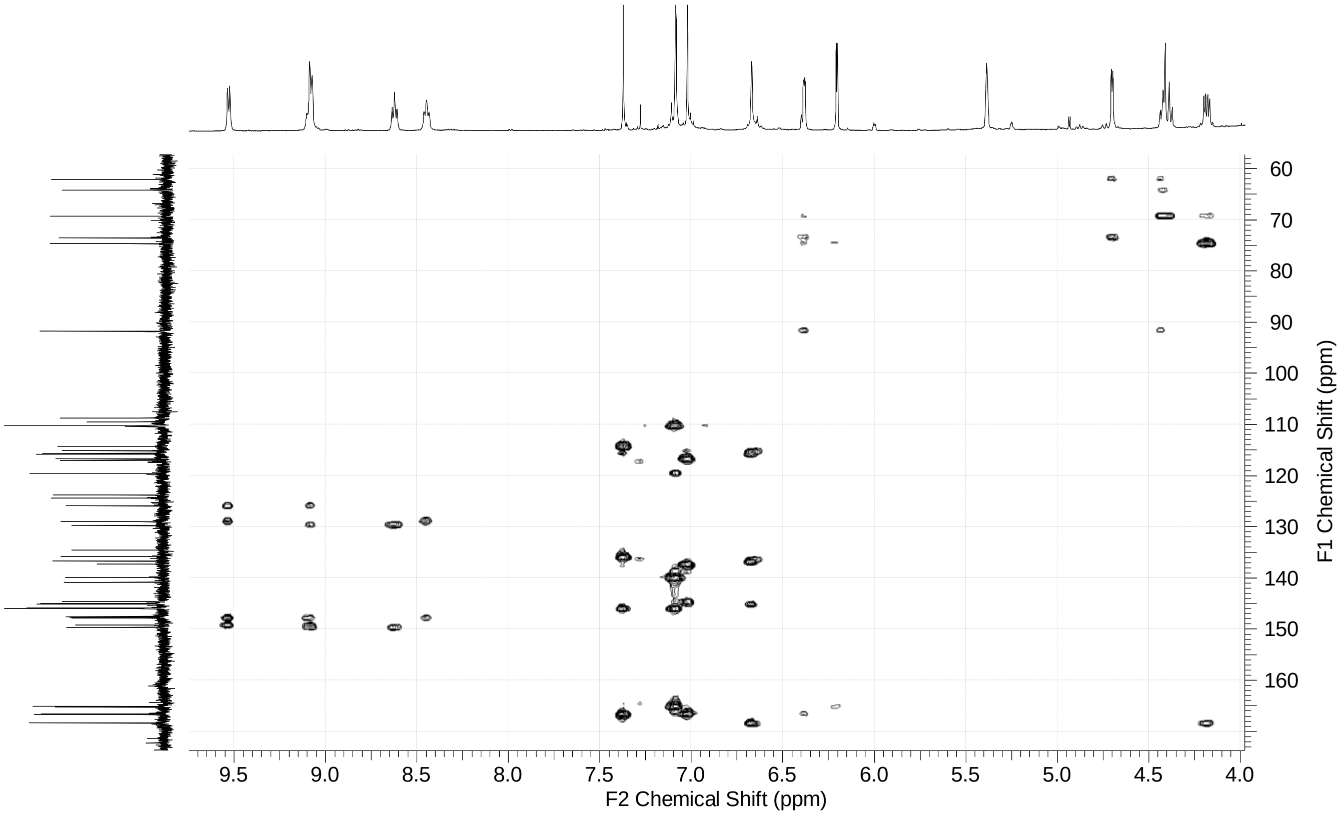

Figure S31. HMBC spectrum of **9** in acetone-d_6

Computational Methods.

A conformational search was performed using the Monte Carlo method and the MMFF94 force field with Spartan ′14 (Wavefunction, Irvine, CA). The obtained low-energy conformers within 6 kcal/mol were optimized at the B3LYP/6-31G(d,p) level in acetone (PCM). The vibrational frequencies were also calculated at the same level to confirm their stability, and no imaginary frequencies were found. ^{1}H NMR coupling constants of the low-energy conformers with Boltzmann populations greater than 1% were calculated at the B3LYP/6-31G(d,p)u+1s (using only the Fermi contact term) level in acetone (PCM) and scaled by using the slope parameter 0.94. The calculated data for each conformer were averaged according to the Boltzmann distribution theory at 298 K based on their relative Gibbs free energies. All DFT calculations were performed using Gaussian 09. GaussView was used to draw the three-dimensional molecular structures.

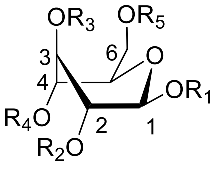

boat form ($^{O,3}B$)

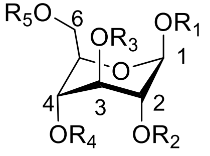

chair form ($^{1}C_4$)

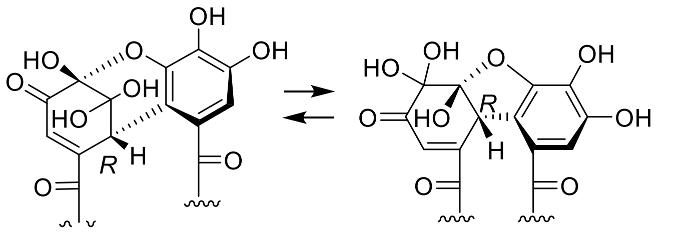

DHHDP (6 membered)   DHHDP (5 membered)

amariin (**1**)
R_1 = galloyl
A: R_2, R_4 = DHHDP (6 membered), R_3, R_5 = DHHDP (6 membered)
B: R_2, R_4 = DHHDP (5 membered), R_3, R_5 = DHHDP (6 membered)
C: R_2, R_4 = DHHDP (6 membered), R_3, R_5 = DHHDP (5 membered)
D: R_2, R_4 = DHHDP (5 membered), R_3, R_5 = DHHDP (5 membered)

acetonyl amariin (**1a**)
R_1 = galloyl
R_2, R_4 = ADHHDP, R_3, R_5 = ADHHDP

geraniin (**2**)
R_1 = galloyl
A: R_2, R_4 = DHHDP (5 membered), R_3, R_5 = HHDP
B: R_2, R_4 = DHHDP (6 membered), R_3, R_5 = HHDP

3,6-dimethoxyfurosin
R_1 = galloyl
A: R_2, R_4 = DHHDP (5 membered), R_3, R_5 = Me
B: R_2, R_4 = DHHDP (6 membered), R_3, R_5 = Me

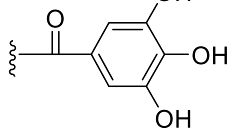

galloyl

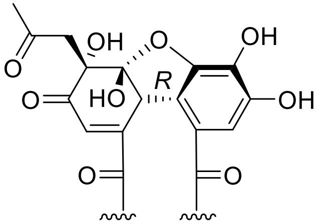

ADHHDP

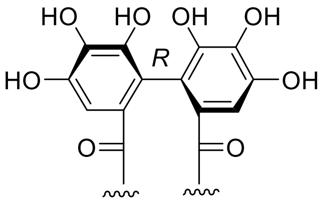

HHDP

Figure S32. Structures of calculated ellagitannins.

Figure S33. Optimized $^{1}C_4$ and $^{O,3}B$ conformers of acetonyl amariin (**1a**) at the B3LYP/6-31G(d,p) level in acetone (PCM).

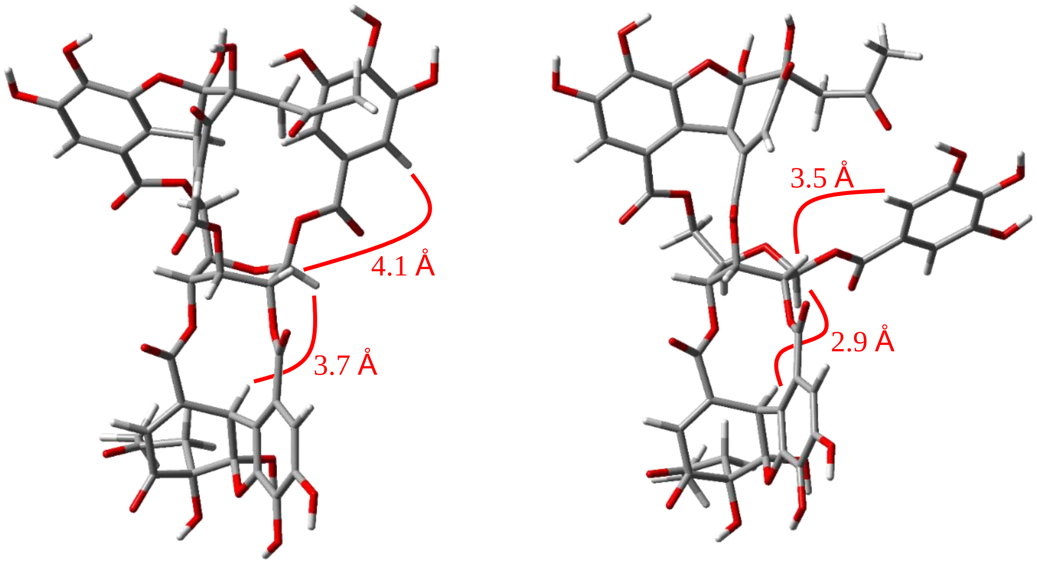

1a ($^{1}C_4$)-1 Δ G = +4.5 kcal/mol

1a ($^{O,3}B$)-1 Δ G = 0.0 kcal/mol

Table S3. Important thermodynamic parameters of most stable conformers of **1a** at the B3LYP/6-31G(d,p) level in acetone (PCM).

conformers	*E* (a.u.)	*E'* (a.u.)	*H* (a.u.)	*G* (a.u.)	Δ*G* (kcal/mol)
**1a ($^{O,3}B$)-1**	-3919.037145	-3918.231827	-3918.164705	-3918.334914	0.00
**1a ($^{1}C_4$)-1**	-3919.030617	-3918.224649	-3918.157568	-3918.327756	4.49

E: total energy; E': total energy with zero point energy; H: enthalpy; G: Gibbs free energy; ΔG: relative Gibbs free energy at the B3LYP/6-31G(d,p) level in acetone (PCM).

Figure S34. Optimized conformers of acetonyl amariin (**1a**) at the B3LYP/6-31G(d,p) level in acetone (PCM) with populations greater than 1% calculated from their relative Gibbs free energies (Δ*G*).

A: Chair form ($^{1}C_{4}$)

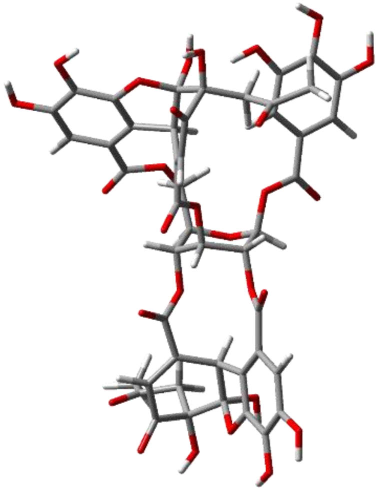

1a ($^{1}C_{4}$)-1

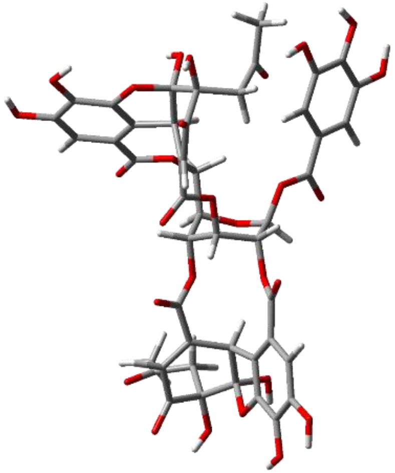

1a ($^{1}C_{4}$)-2

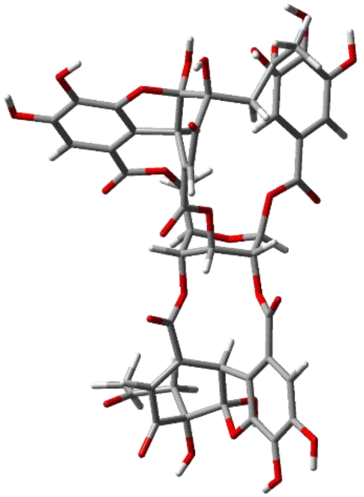

1a ($^{1}C_{4}$)-3

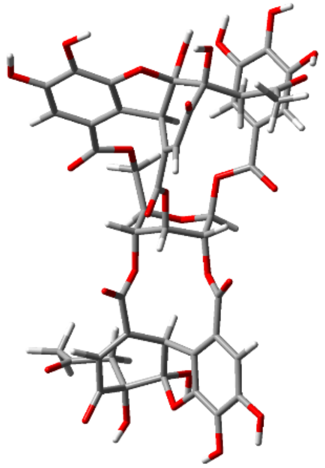

1a ($^{1}C_{4}$)-4

B: Boat form (^{O,3}B)

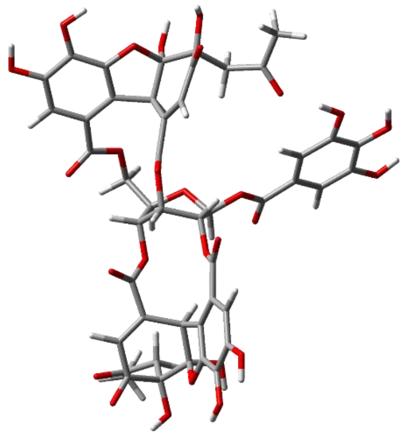

1a (^{O,3}B)-1

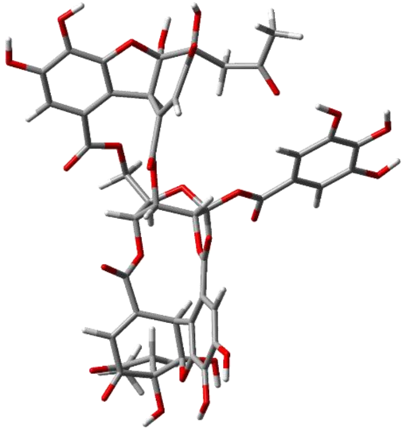

1a (^{O,3}B)-2

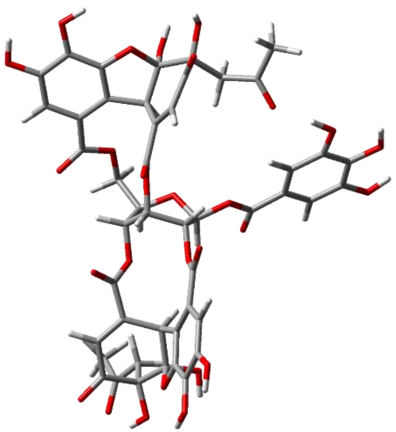

1a (^{O,3}B)-3

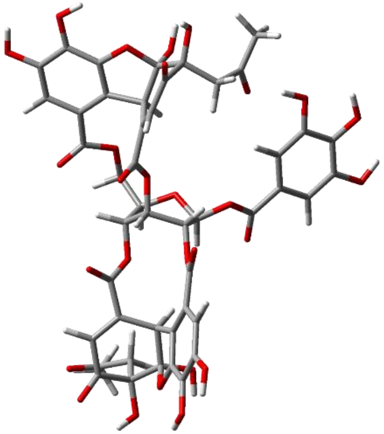

1a (^{O,3}B)-4

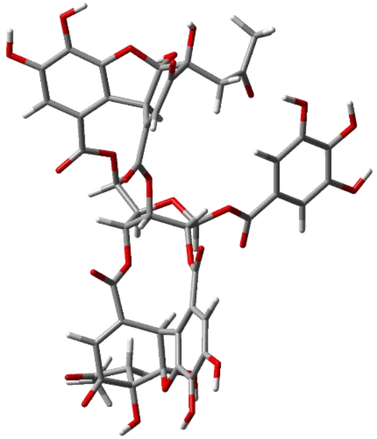

1a (^{O,3}B)-5

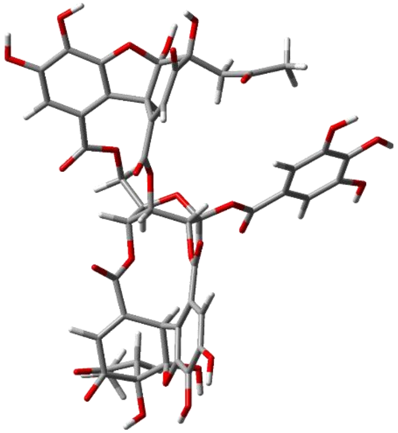

1a (^{O,3}B)-6

Table S2. Important thermodynamic parameters and conformational analysis of **1a** at the B3LYP/6-31G(d,p) level in acetone (PCM). A: Chair form ($^{1}C_{4}$), B: Boat form ($^{O,3}B$).

A

conformers	E (a.u.)	E' (a.u.)	H (a.u.)	G (a.u.)	ΔG (kcal/mol)	P_G (%)
**1a ($^{1}C_{4}$)-1**	-3919.030617	-3918.224649	-3918.157568	-3918.327756	0.00	67.8
**1a ($^{1}C_{4}$)-2**	-3919.029642	-3918.223870	-3918.156881	-3918.326737	0.64	23.0
**1a ($^{1}C_{4}$)-3**	-3919.028136	-3918.222394	-3918.155361	-3918.325573	1.37	6.7
**1a ($^{1}C_{4}$)-4**	-3919.028793	-3918.222819	-3918.156009	-3918.324108	2.29	1.4

E: total energy; E': total energy with zero point energy; H: enthalpy; G: Gibbs free energy; ΔG: relative Gibbs free energy at the B3LYP/6-31G(d,p) level in acetone (PCM). P_G: conformational distribution calculated from relative Gibbs free energy.

B

conformers	E (a.u.)	E' (a.u.)	H (a.u.)	G (a.u.)	ΔG (kcal/mol)	P_G (%)
**1a ($^{O,3}B$)-1**	-3919.037145	-3918.231827	-3918.164705	-3918.334914	0.00	33.5
**1a ($^{O,3}B$)-2**	-3919.037145	-3918.231826	-3918.164705	-3918.334899	0.01	32.9
**1a ($^{O,3}B$)-3**	-3919.038203	-3918.232288	-3918.165473	-3918.334404	0.32	19.5
**1a ($^{O,3}B$)-4**	-3919.037433	-3918.231548	-3918.164648	-3918.333742	0.74	9.7
**1a ($^{O,3}B$)-5**	-3919.035158	-3918.229635	-3918.162571	-3918.332275	1.66	2.0
**1a ($^{O,3}B$)-6**	-3919.031576	-3918.227154	-3918.159481	-3918.331819	1.94	1.3

E: total energy; E': total energy with zero point energy; H: enthalpy; G: Gibbs free energy; ΔG: relative Gibbs free energy at the B3LYP/6-31G(d,p) level in acetone (PCM). P_G: conformational distribution calculated from relative Gibbs free energy.

Table S3. Calculated ^{1}H NMR coupling constants of glucose moiety in **1a** (ppm). A: Chair form ($^{1}C_4$), B: Boat form ($^{O,3}B$).

A

	calculated[a]						experimental[b]
	**1a ($^{1}C_4$)-1**	**1a ($^{1}C_4$)-2**	**1a ($^{1}C_4$)-3**	**1a ($^{1}C_4$)-4**	averaged-**1a** ($^{1}C_4$)[c]	averaged-**1a** ($^{1}C_4$) (corrected)[d]	**1a**
$J_{1\text{–}2}$	0.9	0.4	0.8	1.3	0.8	0.7	4.9
$J_{2\text{-}3}$	2.1	1.6	2.0	2.4	2.0	1.8	1.5
$J_{3\text{-}4}$	3.5	3.5	3.5	3.4	3.5	3.3	3.8
$J_{4\text{-}5}$	1.6	2.2	1.7	1.3	1.7	1.6	1.4
$J_{5\text{-}6a}$	2.7	7.7	4.7	1.1	4.0	3.7	1.2
$J_{5\text{-}6b}$	10.4	10.4	10.9	9.2	10.4	9.8	3.2
$J_{6a\text{-}6b}$	14.3	13.8	14.2	14.6	14.2	13.4	13.1
$J_{2\text{-}4}$	1.4	1.3	1.3	1.4	1.3	1.3	1.4

[a] *Calculated at the B3LYP/6-31G(d,p)u+1s (using only the Fermi contact term) level in acetone (PCM).* [b] *Measured in acetone (500 MHz).* [c] *Averaged according to the Boltzmann distribution theory at 298 K based on relative Gibbs free energies.* [d] *Scaled by using the slope parameter 0.94.*

B

	calculated[a]								experimental[b]
	**1a ($^{O,3}B$)-1**	**1a ($^{O,3}B$)-2**	**1a ($^{O,3}B$)-3**	**1a ($^{O,3}B$)-4**	**1a ($^{O,3}B$)-5**	**1a ($^{O,3}B$)-6**	averaged-**1a** ($^{O,3}B$)[c]	averaged-**1a** ($^{O,3}B$) (corrected)[d]	**1a**
$J_{1\text{–}2}$	5.0	5.0	5.0	5.3	5.3	5.4	5.1	4.8	4.9
$J_{2\text{-}3}$	1.8	1.8	1.7	1.4	1.4	1.5	1.7	1.6	1.5
$J_{3\text{-}4}$	4.3	4.3	4.3	4.1	4.1	4.2	4.3	4.0	3.8
$J_{4\text{-}5}$	1.0	1.0	1.0	1.1	1.1	1.0	1.0	0.9	1.4
$J_{5\text{-}6a}$	1.9	1.9	1.9	1.8	2.0	1.7	1.9	1.8	1.2
$J_{5\text{-}6b}$	2.4	2.4	2.4	2.5	2.4	2.6	2.4	2.3	3.2
$J_{6a\text{-}6b}$	14.0	14.0	14.0	13.9	13.9	14.0	14.0	13.2	13.1
$J_{2\text{-}4}$	1.7	1.7	1.7	1.6	1.6	1.7	1.7	1.6	1.4

[a] *Calculated at the B3LYP/6-31G(d,p)u+1s (using only the Fermi contact term) level in acetone (PCM).* [b] *Measured in acetone (500 MHz).* [c] *Averaged according to the Boltzmann distribution theory at 298 K based on relative Gibbs free energies.* [d] *Scaled by using the slope parameter 0.94.*

Figure S35. Optimized $^{1}C_4$ and $^{O,3}B$ conformers of amariin (**1**) at the B3LYP/6-31G(d,p) level in acetone (PCM).

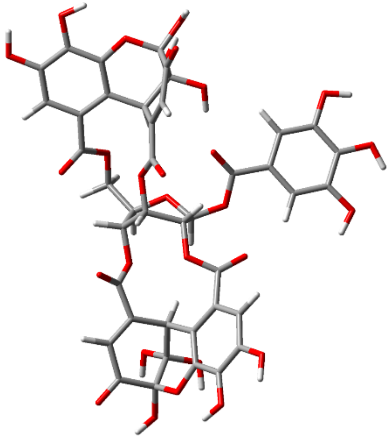

1–A (^{O,3}B)

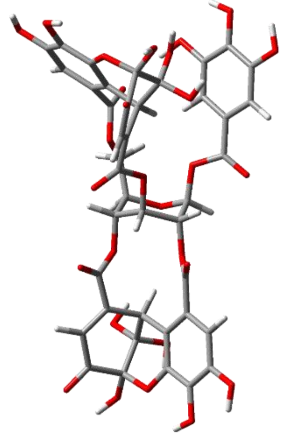

1–A ($^{1}C_4$)

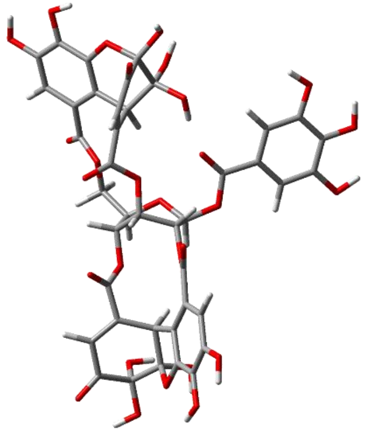

1–B (^{O,3}B)

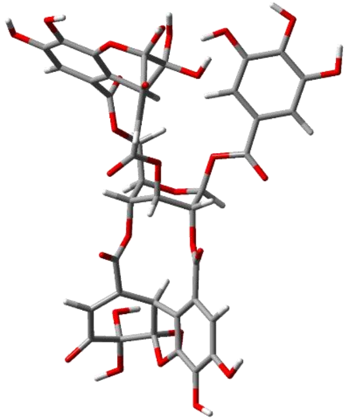

1–B ($^{1}C_4$)

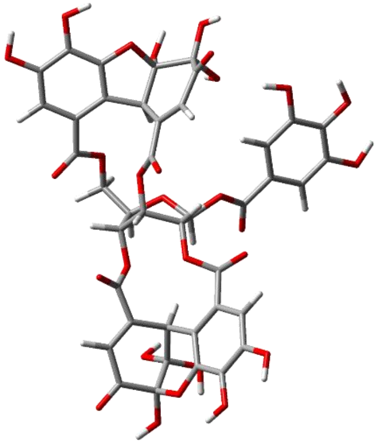

1–C (^{O,3}B)

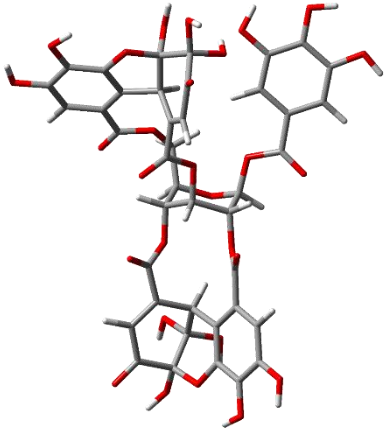

1–C ($^{1}C_4$)

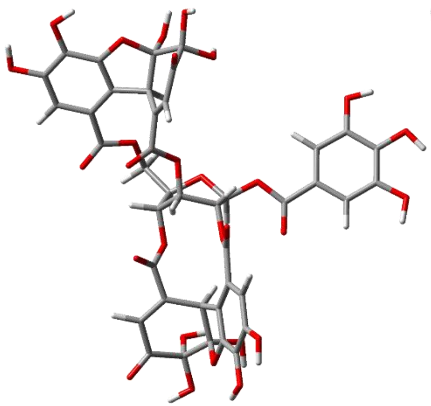

1–D (^{O,3}B)

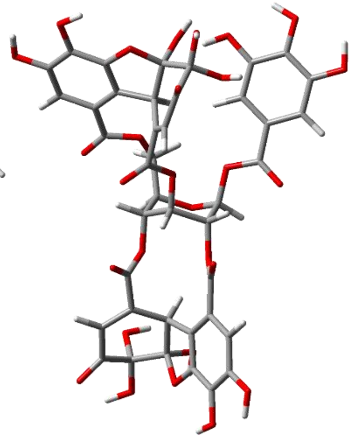

1–D ($^{1}C_4$)

Table S4. Important thermodynamic parameters and conformational analysis of **1** at the B3LYP/6-31G(d,p) level in acetone (PCM).

conformers	E (a.u.)	E' (a.u.)	H (a.u.)	G (a.u.)	ΔG (kcal/mol)
**1–A ($^{O,3}B$)**	-3685.559950	-3684.875447	-3684.816344	-3684.966683	0.01
**1–A ($^{1}C_4$)**	-3685.559488	-3684.874355	-3684.815433	-3684.965775	0.58
**1–B ($^{O,3}B$)**	-3685.556145	-3684.872692	-3684.813042	-3684.966706	0.00
**1–B ($^{1}C_4$)**	-3685.556909	-3684.872064	-3684.813060	-3684.964099	1.64
**1–C ($^{O,3}B$)**	-3685.559637	-3684.875226	-3684.816185	-3684.966441	0.17
**1–C ($^{1}C_4$)**	-3685.557725	-3684.873229	-3684.814041	-3684.965099	1.01
**1–D ($^{O,3}B$)**	-3685.552323	-3684.869791	-3684.809697	-3684.964947	1.10
**1–D ($^{1}C_4$)**	-3685.555858	-3684.871943	-3684.812508	-3684.963745	1.86

E: total energy; E': total energy with zero point energy; H: enthalpy; G: Gibbs free energy; ΔG: relative Gibbs free energy at the B3LYP/6-31G(d,p) level in acetone (PCM).

Figure S36. Optimized $^{1}C_4$ and $^{O,3}B$ conformers of geraniin (**2**) at the B3LYP/6-31G(d,p) level in acetone (PCM).

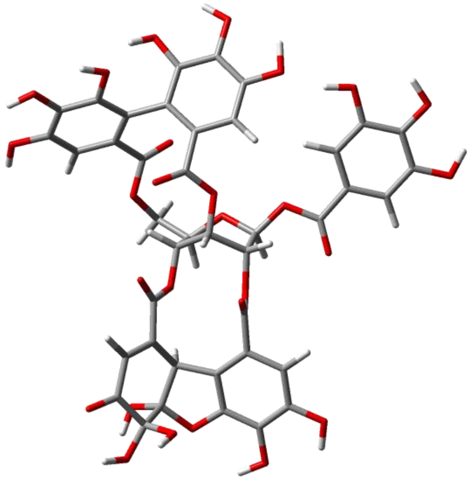

2–A (^{O,3}B)

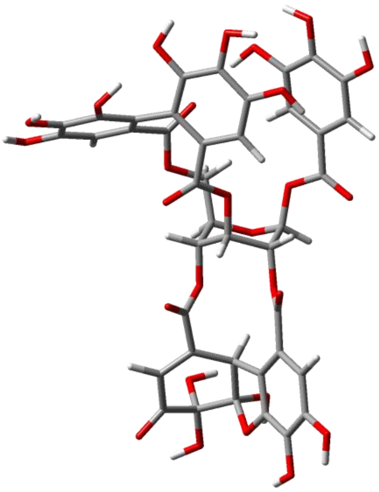

2–A (^{1}C$_4$)

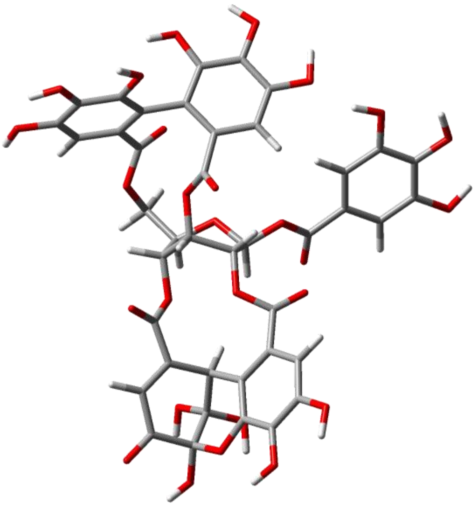

2–B (^{O,3}B)

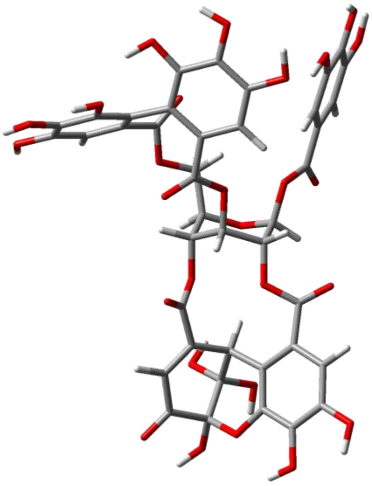

2–B (^{1}C$_4$)

Table S5. Important thermodynamic parameters and conformational analysis of **2** at the B3LYP/6-31G(d,p) level in acetone (PCM).

conformers	E (a.u.)	E' (a.u.)	H (a.u.)	G (a.u.)	ΔG (kcal/mol)
**2–A (^{O,3}B)**	-3610.345974	-3609.667655	-3609.608438	-3609.760436	8.37
**2–A (^{1}C$_4$)**	-3610.357796	-3609.679650	-3609.620037	-3609.771910	1.17
**2–B (^{O,3}B)**	-3610.352473	-3609.674640	-3609.614965	-3609.767234	4.10
**2–B (^{1}C$_4$)**	-3610.361707	-3609.682788	-3609.623667	-3609.773775	0.00

E: total energy; E': total energy with zero point energy; H: enthalpy; G: Gibbs free energy; ΔG: relative Gibbs free energy at the B3LYP/6-31G(d,p) level in acetone (PCM).

Figure S37. Optimized $^1C_4$ and $^{O,3}B$ conformers of 3,6-dimethoxyfurosin at the B3LYP/6-31G(d,p) level in acetone (PCM).
*In order to avoid hydrogen bonds between hydroxy moieties of 3 and 6 position, calculation was performed with 3,6-dimethoxyfurosin.

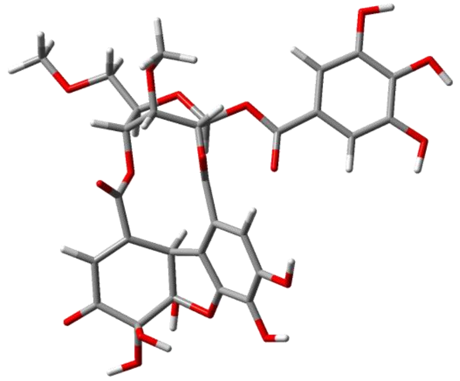

fro–A ($^{O,3}B$)

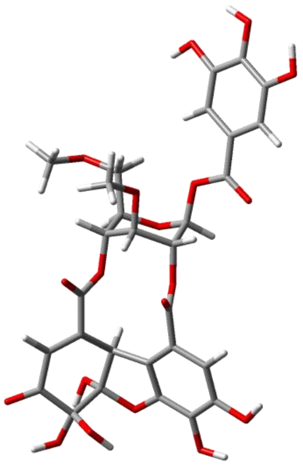

fro–A ($^1C_4$)

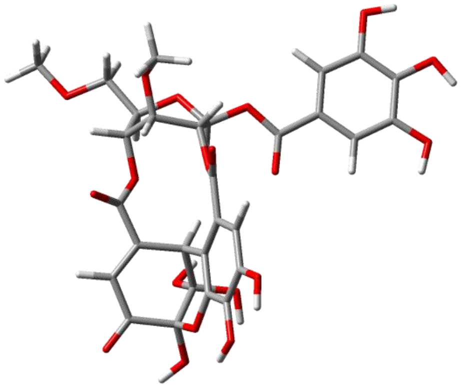

fro–B ($^{O,3}B$)

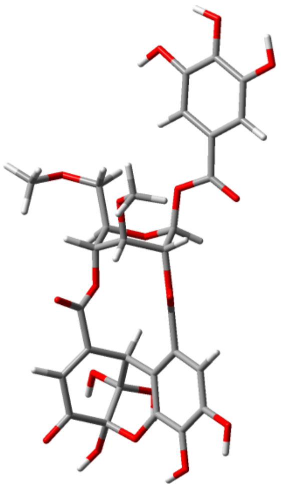

fro–B ($^1C_4$)

Table S6. Important thermodynamic parameters and conformational analysis of 3,6-dimethoxyfurosin at the B3LYP/6-31G(d,p) level in acetone (PCM).

conformers	E (a.u.)	E' (a.u.)	H (a.u.)	G (a.u.)	ΔG (kcal/mol)
**fro–A ($^{O,3}B$)**	-2550.010098	-2549.462581	-2549.417838	-2549.540987	6.31
**fro–A ($^1C_4$)**	-2550.009996	-2549.461914	-2549.417067	-2549.542069	5.63
**fro–B ($^{O,3}B$)**	-2550.018183	-2549.470502	-2549.425640	-2549.548647	1.51
**fro–B ($^1C_4$)**	-2550.019129	-2549.470926	-2549.426129	-2549.551048	0.00

E: total energy; E': total energy with zero point energy; H: enthalpy; G: Gibbs free energy; ΔG: relative Gibbs free energy at the B3LYP/6-31G(d,p) level in acetone (PCM).

Cartesian coordinates of the optimized geometries.

Cartesian coordinates of the low-energy conformer of acetonyl amariin [**1a ($^1C_4$)-1**] at the B3LYP/6-31G(d,p) level in acetone (PCM).

C	-1.39403500	1.11690200	-1.63587000
O	-0.67237300	1.64438300	-2.45285900
C	-1.63002600	2.73622200	1.72296300
O	-0.74171800	3.39764100	2.23386900
C	-2.89105400	1.11005400	-1.70146600
C	-3.42357300	1.07371900	-2.93912300
C	-4.87699300	0.92629900	-3.14186000
C	-5.61105400	0.22281100	-1.99215900
C	-5.23351000	1.04135800	-0.73433600
C	-3.71961700	1.16348300	-0.41686600
C	-3.67551400	2.57762400	0.17502400
C	-4.81144700	3.22220000	-0.30375500
C	-5.08965800	4.56357800	-0.08511800
C	-4.15368600	5.30281800	0.66180400
C	-3.03019600	4.67342600	1.19071500
C	-2.77953200	3.30532500	0.97548200
O	-4.36567900	6.62244300	0.90669400
O	-6.19609300	5.21284800	-0.53465700
O	-5.49772200	1.39499300	-4.08236600
C	-5.22951100	-1.25866100	-1.80704800
O	-5.90476500	0.57628100	0.40459200
O	-5.66164700	2.40248900	-1.00676900
H	-3.39563500	0.39843100	0.28684700
O	-7.00457000	0.29726200	-2.18908800
C	0.67769200	0.47135400	2.10070500
C	1.01909700	1.03000200	0.71249000
C	0.46617500	0.19998100	-0.46093800
C	0.75214500	-1.29611100	-0.26292600
C	0.55752400	-1.79913200	1.17234400
O	1.01077800	-0.93035000	2.17019100
O	2.46313000	0.89637600	0.67000700
O	2.14890800	-1.50642700	-0.57039600
C	-0.72664300	0.76517000	2.69802900
O	-0.95475300	0.38120700	-0.58361400
O	-0.84333300	-2.09088100	1.34075600
C	-1.13585500	-3.21757200	2.07355100
O	-0.27426000	-3.97881700	2.47153100
C	-2.58510300	-3.39662900	2.30540000
C	-2.98358200	-4.57198000	2.95862400
C	-4.33294100	-4.80250800	3.20352700
C	-5.28241200	-3.85785800	2.79262400
C	-4.88392500	-2.68029100	2.14076900
C	-3.53389500	-2.44321000	1.89592000
O	-4.72815600	-5.94137900	3.83844400
O	-6.59233400	-4.12459700	3.05091400
O	-5.92129900	-1.86358800	1.79572700
O	-1.69194500	1.38944400	1.82785500
C	3.17433200	1.52441400	-0.28751300
O	2.72654400	2.34055900	-1.06353400
C	2.44859500	-1.67232200	-1.88881800
O	1.59117300	-1.82312600	-2.73928600
C	4.61019800	1.10288200	-0.17107400
C	5.53551900	2.08011600	-0.20539100
C	6.94053200	1.79569700	0.14922800
C	7.11341200	0.62459700	1.12869900
C	6.42985200	-0.58114800	0.43484500
C	4.94032900	-0.37949700	0.02729700
C	4.90409300	-1.13987700	-1.30990600
C	6.22998400	-1.24990800	-1.72058200

C	6.62834800	-1.82490700	-2.91970000
C	5.62314900	-2.30979400	-3.77370900
C	4.28946400	-2.22602000	-3.38839400
C	3.90639900	-1.65830700	-2.15757200
O	5.95012900	-2.88165900	-4.96295700
O	7.92130400	-1.94876600	-3.32182600
O	7.91310900	2.35703600	-0.32935700
C	6.48956800	0.85126200	2.51905000
O	6.53613600	-1.74300100	1.18438500
O	7.14196200	-0.75065000	-0.82983700
H	4.28278900	-0.81723100	0.77876800
O	8.48408400	0.34624800	1.30732400
C	-5.32806800	-2.06767700	-3.09659400
O	-4.95611100	-1.58504300	-4.15393000
C	-5.85614900	-3.47384300	-2.98340200
C	6.86695500	2.18746300	3.14587100
C	7.02747100	2.21797500	4.64422500
O	6.99278300	3.18414000	2.45183700
H	-2.78958700	1.15041100	-3.81609900
H	-2.34839800	5.24513500	1.80814900
H	-5.19726000	6.88601100	0.48181900
H	-6.77357400	4.59640900	-1.00945000
H	-4.19350500	-1.36111700	-1.46288100
H	-5.86426300	-1.68269400	-1.02568000
H	-6.85038000	0.54353700	0.17294500
H	-7.13156000	0.89072500	-2.95346900
H	1.40631900	0.91953500	2.77928400
H	0.74390600	2.08109200	0.61843900
H	0.93015800	0.54099600	-1.38600900
H	0.13514100	-1.88282400	-0.94357700
H	1.12077200	-2.72075100	1.31214400
H	-1.18471700	-0.17721800	2.98792200
H	-0.60676300	1.39932100	3.57667700
H	-2.24505000	-5.29927100	3.27208800
H	-3.21081800	-1.53458300	1.40571000
H	-5.69426000	-5.92133000	3.92384600
H	-7.12205200	-3.37676300	2.72972400
H	-5.63714900	-1.02519200	1.38404700
H	5.26208900	3.10241100	-0.44648200
H	3.52637600	-2.62032300	-4.04786400
H	6.91527200	-2.86124500	-5.05965400
H	8.51279300	-1.61613800	-2.62968700
H	5.39410900	0.83432300	2.47084600
H	6.78255600	0.01799300	3.16186800
H	7.47447300	-1.83449100	1.42305000
H	8.94915500	0.87998700	0.63574600
H	-6.91234500	-3.43769600	-2.69094800
H	-5.75489500	-3.99779300	-3.93428000
H	-5.32811200	-4.01832200	-2.19292200
H	7.14788300	3.24382000	4.99386500
H	6.16535200	1.75050700	5.13219900
H	7.90781100	1.62694500	4.92343700

Cartesian coordinates of the low-energy conformer of acetonyl amariin [**1a ($^1C_4$)-2**] at the B3LYP/6-31G(d,p) level in acetone (PCM).

C	-1.43763200	-1.73336900	1.12015400
O	-0.67132000	-2.66151900	1.25054700
C	-1.92340400	-1.92409800	-2.45508100
O	-1.17361100	-2.12469600	-3.39348600
C	-2.91642300	-1.84087100	1.35757900
C	-3.28991700	-2.30430900	2.56715400
C	-4.69838000	-2.32619700	2.97968500
C	-5.66169700	-1.44174500	2.16716700
C	-5.37540700	-1.71925100	0.68223500
C	-3.89372600	-1.52130200	0.23284400
C	-3.78693700	-2.61133900	-0.84419300
C	-4.78727700	-3.53231700	-0.54295200
C	-4.97486400	-4.71812500	-1.24173700
C	-4.09492100	-4.99227000	-2.30485100
C	-3.10882500	-4.07093700	-2.65127400
C	-2.94042800	-2.87232900	-1.93836800
O	-4.22946400	-6.13644200	-3.02383300
O	-5.94886300	-5.62713500	-0.97491600
O	-5.12257900	-2.93744400	3.94994100
C	-5.37397400	0.04086800	2.56782500
O	-6.27120900	-0.97835800	-0.07363800
O	-5.61379800	-3.14974900	0.48192300
H	-3.73733000	-0.50777200	-0.13889700
O	-6.98662900	-1.79564400	2.46425700
C	0.38271700	0.24285000	-1.87141700
C	0.80404400	-0.73796900	-0.77397200
C	0.36678700	-0.29776700	0.63206100
C	0.74367600	1.17141600	0.88806300
C	0.64286300	2.12473000	-0.31676000
O	0.89715700	1.55421500	-1.57389600
O	2.25132700	-0.66566200	-0.80819300
O	2.15277900	1.16966900	1.22061500
C	-1.12774700	0.32776400	-2.21220400
O	-1.05628400	-0.46864200	0.81135100
O	-0.65085400	2.75951700	-0.28988000
C	-0.66186500	4.05263800	-0.77037400
O	0.36425500	4.63600000	-1.06791100
C	-2.01498800	4.63290700	-0.89501400
C	-2.08391200	5.92917100	-1.43063600
C	-3.31952400	6.54055700	-1.60863000
C	-4.48507600	5.85326400	-1.25221400
C	-4.42088900	4.55815800	-0.71227000
C	-3.18332200	3.93985700	-0.52844400
O	-3.39367900	7.79994300	-2.12777900
O	-5.67990100	6.48003000	-1.44109700
O	-5.63453100	4.01971200	-0.42370700
O	-1.93285800	-0.77152200	-1.75181100
C	2.98734500	-1.60305800	-0.17497400
O	2.54873600	-2.62586200	0.30286800
C	2.47714600	0.83431700	2.49849000
O	1.64229300	0.72423100	3.37734200
C	4.43374900	-1.20411100	-0.23001000
C	5.30956900	-2.14823800	-0.62246700
C	6.71226900	-1.79617300	-0.91917500
C	6.90983400	-0.34531300	-1.38189500
C	6.31299600	0.52020700	-0.24352000
C	4.82877000	0.23187500	0.12764100
C	4.87479500	0.42717900	1.65364100
C	6.21559900	0.31125100	2.01058600
C	6.67915000	0.36457400	3.31825000

C	5.72814300	0.53436700	4.33927500
C	4.38246200	0.67150500	4.01534700
C	3.93417400	0.63434700	2.68103000
O	6.12135500	0.59186700	5.63935600
O	7.98683500	0.26433200	3.67681300
O	7.67126100	-2.53148700	-0.74562300
C	6.22798600	-0.00017400	-2.71984000
O	6.46027700	1.87666000	-0.48776700
O	7.07069400	0.15441600	0.95300800
H	4.17047000	0.94336200	-0.37131900
O	8.28593500	-0.06484800	-1.50955200
C	-6.00571200	1.10248100	1.68300000
O	-5.26445100	1.78846200	0.98168900
C	-7.48723600	1.32713100	1.76702200
C	6.52637400	-1.00200000	-3.82842000
C	6.64743400	-0.45760000	-5.22868300
O	6.62271200	-2.19297600	-3.57694900
H	-2.55503600	-2.63740800	3.29294500
H	-2.46918100	-4.27547400	-3.50126100
H	-4.97079300	-6.64601800	-2.65997900
H	-6.52045900	-5.30033000	-0.26353400
H	-5.75410600	0.15134400	3.58882400
H	-4.30102200	0.23680100	2.57544200
H	-6.01172400	-0.99417400	-1.00755100
H	-6.92901500	-2.34262300	3.27119400
H	0.93047200	-0.04940200	-2.76908700
H	0.48206700	-1.75824800	-0.98244700
H	0.86771300	-0.92548000	1.36862200
H	0.16614700	1.56715800	1.72413700
H	1.39752700	2.90040100	-0.20033200
H	-1.56089300	1.19461700	-1.72130900
H	-1.24444700	0.42520800	-3.29276200
H	-1.17746800	6.45129100	-1.70996800
H	-3.13297600	2.94570100	-0.10191500
H	-4.32923900	8.05191500	-2.17290500
H	-6.37571700	5.88181800	-1.12060600
H	-5.54967200	3.16823000	0.07310500
H	4.99669900	-3.17745400	-0.76647900
H	3.66285900	0.82562200	4.80986900
H	7.08578700	0.49182600	5.67400100
H	8.54005700	0.19172500	2.88420400
H	5.13641600	0.02518700	-2.61611000
H	6.53346100	1.00893800	-3.00611700
H	7.39324500	2.01656400	-0.72432200
H	8.74607100	-0.83420400	-1.12399500
H	-8.01246300	0.37010800	1.79126500
H	-7.70506500	1.83968200	2.71248800
H	-7.83401200	1.94630900	0.93888500
H	6.71007300	-1.27283700	-5.95019200
H	5.79639800	0.19090200	-5.46330200
H	7.54766300	0.16485500	-5.29602400

Cartesian coordinates of the low-energy conformer of acetonyl amariin [**1a ($^1C_4$)-3**] at the B3LYP/6-31G(d,p) level in acetone (PCM).

C	1.34799500	1.48012800	1.31416100
O	0.61050400	2.37869900	1.65240500
C	1.72938300	2.19718100	-2.17689600
O	0.91435700	2.63484800	-2.97010100
C	2.83521200	1.49212400	1.51930600
C	3.25422000	1.79744800	2.76437000
C	4.67378100	1.73247500	3.12957900
C	5.55142600	0.86528100	2.21944200
C	5.25942100	1.31461100	0.77359800
C	3.76921100	1.27135400	0.33400700
C	3.70320000	2.51003500	-0.56797900
C	4.77261300	3.31315600	-0.18398400
C	5.01229300	4.57585900	-0.70893400
C	4.10702900	5.06154000	-1.67044000
C	3.04417100	4.26731400	-2.09232800
C	2.82740100	2.98283200	-1.56128000
O	4.28844700	6.29222600	-2.21755800
O	6.05625100	5.37403500	-0.36037000
O	5.16473600	2.27402400	4.11001200
C	5.20132800	-0.63865200	2.47591700
O	6.01554700	0.64009000	-0.16930700
O	5.59637600	2.74141900	0.75059700
H	3.54503900	0.34325800	-0.19075800
O	6.91217400	1.10079400	2.50154500
C	-0.59667200	-0.09026400	-1.94019200
C	-0.97609200	0.75668300	-0.71967900
C	-0.49703900	0.17128500	0.62163000
C	-0.84422600	-1.31980600	0.72979500
C	-0.64095700	-2.12233600	-0.56106700
O	-1.00876700	-1.45841500	-1.73972500
O	-2.42367200	0.67397200	-0.71943800
O	-2.25648400	-1.40507800	1.02428000
C	0.86403100	-0.00950400	-2.46569700
O	0.93025800	0.31171300	0.77236200
O	0.74115700	-2.51632900	-0.60892600
C	0.99282600	-3.76251700	-1.13833500
O	0.10755200	-4.56931100	-1.35136100
C	2.42363300	-3.98137600	-1.43106400
C	2.77372500	-5.20390000	-2.02862700
C	4.09164400	-5.44516900	-2.39551400
C	5.06134500	-4.45773400	-2.16403100
C	4.71605700	-3.23992700	-1.56002200
C	3.39629300	-2.99532200	-1.19144300
O	4.43863700	-6.63025600	-2.97280500
O	6.34193300	-4.71635900	-2.53548800
O	5.74347900	-2.33929500	-1.44471100
O	1.74112600	0.90415400	-1.77751700
C	-3.15816200	1.52559800	0.02428300
O	-2.72266700	2.49099500	0.61239100
C	-2.60402200	-1.24315700	2.33064100
O	-1.78023300	-1.21347100	3.22631300
C	-4.60127100	1.12253800	-0.07069400
C	-5.49112700	2.10334800	-0.31269700
C	-6.89091800	1.78052000	-0.65359500
C	-7.07230900	0.41538600	-1.33365800
C	-6.45676400	-0.60852100	-0.34681500
C	-4.97604000	-0.35606200	0.06519900
C	-5.01493200	-0.78441700	1.54256600
C	-6.35743000	-0.75605900	1.91137100
C	-6.81825700	-1.02467900	3.19308200

C	-5.86218600	-1.32663800	4.17789200
C	-4.51392900	-1.37450200	3.84043100
C	-4.06785100	-1.12115400	2.52864300
O	-6.25211100	-1.59646400	5.45199700
O	-8.12780900	-1.01492200	3.55892000
O	-7.85865700	2.46618500	-0.36418600
C	-6.39606700	0.28782200	-2.71278900
O	-6.57809200	-1.91205300	-0.80365300
O	-7.21830400	-0.45277600	0.89109200
H	-4.30828300	-0.97361600	-0.53606400
O	-8.44563300	0.13987700	-1.49718700
C	6.38080400	-1.57727000	2.21918600
O	6.57992800	-2.06870300	1.11586100
C	7.26408500	-1.90726100	3.39073100
C	-6.71324400	1.44342900	-3.65305300
C	-6.83518900	1.11810600	-5.11983200
O	-6.82387100	2.58030000	-3.22169800
H	2.54968900	2.08497400	3.53797600
H	2.38256700	4.63851000	-2.86533300
H	5.08320700	6.68913800	-1.82734300
H	6.63783100	4.90548800	0.25734600
H	4.90062300	-0.74816200	3.52214200
H	4.36367100	-0.95342000	1.85069100
H	6.94123900	0.66936700	0.12398300
H	6.92448400	1.72195100	3.25479300
H	-1.25514500	0.24531000	-2.74360900
H	-0.66708000	1.79698900	-0.82708800
H	-0.97604000	0.71779000	1.43405400
H	-0.27221700	-1.77488600	1.53861600
H	-1.25762300	-3.01971800	-0.52942300
H	1.33799500	-0.97994000	-2.35074400
H	0.84448600	0.26531900	-3.52052100
H	2.01532800	-5.95436100	-2.21550100
H	3.12378700	-2.05207100	-0.73615600
H	5.39069900	-6.60937100	-3.15716100
H	6.86614500	-3.93694800	-2.27620400
H	5.85062500	-2.03509500	-0.51546000
H	-5.19106300	3.14629200	-0.30263400
H	-3.78960800	-1.62764100	4.60461400
H	-7.21909300	-1.53173400	5.49796800
H	-8.68382900	-0.84727000	2.78288200
H	-5.30345800	0.25808500	-2.62156900
H	-6.69464800	-0.66841700	-3.14851000
H	-7.50918800	-2.03275900	-1.05717100
H	-8.91281000	0.83466600	-0.99589400
H	7.61594400	-0.98121700	3.85413600
H	6.67943600	-2.44626700	4.14571000
H	8.10647000	-2.52141900	3.07137500
H	-5.98097700	0.51891400	-5.45313500
H	-7.73159800	0.50686400	-5.27852300
H	-6.90657700	2.03322700	-5.70854700

Cartesian coordinates of the low-energy conformer of acetonyl amariin [**1a ($^1C_4$)-4**] at the B3LYP/6-31G(d,p) level in acetone (PCM).

C	-1.34286000	0.35293100	-1.84655000
O	-0.66982000	0.38546600	-2.85291700
C	-1.49168000	3.23751200	0.50335800
O	-0.58658200	4.05542200	0.54135900
C	-2.84210400	0.28726300	-1.82053800
C	-3.42006600	-0.27249500	-2.90348300
C	-4.86371600	-0.55976100	-2.95525700
C	-5.54440400	-0.70422700	-1.59050300
C	-5.14249100	0.57307800	-0.80361000
C	-3.62291500	0.84255100	-0.63170700
C	-3.60422300	2.37543600	-0.70572400
C	-4.76650800	2.73155100	-1.38666000
C	-5.07753400	4.03087100	-1.76061600
C	-4.14659800	5.03482300	-1.44092700
C	-2.98979500	4.71249000	-0.73710900
C	-2.70415100	3.39121100	-0.34277000
O	-4.38974900	6.32737700	-1.78381300
O	-6.21230600	4.40041100	-2.41315900
O	-5.50046600	-0.67816900	-3.99201200
C	-5.12156100	-1.96383100	-0.80110100
O	-5.75935000	0.63760600	0.43212400
O	-5.60637200	1.67964200	-1.64716900
H	-3.25511000	0.44339600	0.31407100
O	-6.95148800	-0.72673100	-1.71979000
C	0.88047800	1.37010700	1.79811500
C	1.19347800	1.30884600	0.29602400
C	0.57285500	0.10295700	-0.43676100
C	0.79599700	-1.19851200	0.34636000
C	0.54879400	-1.05215800	1.85431100
O	1.11299900	0.08514900	2.41753600
O	2.62924100	1.10402200	0.28697800
O	2.18243600	-1.57499000	0.19547700
C	-0.44781500	2.02746900	2.25176300
O	-0.84590000	0.27966000	-0.58928600
O	-0.87161500	-1.01012400	2.12884400
C	-1.54826700	-2.19102000	2.12873500
O	-1.05027600	-3.22947100	1.72633800
C	-2.92839800	-2.04965600	2.64256100
C	-3.85668700	-3.04524600	2.29940100
C	-5.17415500	-2.92634400	2.73106400
C	-5.54895100	-1.85325400	3.55229100
C	-4.61186700	-0.87844800	3.92419100
C	-3.30526600	-0.96636400	3.45259400
O	-6.17931100	-3.79868800	2.40508000
O	-6.82878100	-1.71275700	3.98935900
O	-4.98106900	0.16462200	4.71963000
O	-1.50740100	2.12763900	1.27655000
C	3.33625100	1.25027500	-0.85059900
O	2.89603000	1.68421600	-1.89327600
C	2.47906700	-2.28584400	-0.92816300
O	1.61875100	-2.73452100	-1.66288700
C	4.75994000	0.86474700	-0.57415100
C	5.71728000	1.68860700	-1.03965700
C	7.11817700	1.53335100	-0.59829000
C	7.26775100	0.91708700	0.80161400
C	6.52974400	-0.44411800	0.72101600
C	5.04312500	-0.38885100	0.25829000
C	4.96275700	-1.66111900	-0.60326400
C	6.27901600	-1.99778100	-0.90894500
C	6.64213700	-3.06365100	-1.72072800

C	5.60983700	-3.83540200	-2.28012900
C	4.28434600	-3.52977400	-1.99065000
C	3.93622000	-2.45787300	-1.14549400
O	5.90177300	-4.89191600	-3.08449600
O	7.92503600	-3.40877600	-2.01099300
O	8.10117900	1.77916100	-1.27897600
C	6.67885700	1.76674600	1.94411800
O	6.60013600	-1.14768100	1.91433300
O	7.21998600	-1.19177800	-0.32658600
H	4.38111700	-0.42602000	1.12377500
O	8.63122400	0.69539800	1.08401100
C	-5.78100400	-3.28005300	-1.20460600
O	-6.15786500	-4.05713100	-0.33250900
C	-5.89400400	-3.65508900	-2.65636400
C	7.10443000	3.22845000	1.89597000
C	7.30300700	3.92747000	3.21637600
O	7.23799700	3.79922500	0.82474200
H	-2.82157800	-0.54108600	-3.76789400
H	-2.30489000	5.50526800	-0.46330600
H	-5.24192400	6.36853600	-2.24585400
H	-6.79073800	3.63164400	-2.52920200
H	-4.03751900	-2.11331400	-0.87045200
H	-5.35302000	-1.79212200	0.25045100
H	-6.70279500	0.45180100	0.28242100
H	-7.15871700	-0.41159500	-2.61677600
H	1.67384000	1.97961400	2.23638800
H	0.94813900	2.24640600	-0.20442400
H	1.01637700	0.02148900	-1.42924700
H	0.16240500	-1.98864900	-0.05254200
H	0.98406300	-1.90961500	2.37144100
H	-0.85849200	1.43172500	3.06415300
H	-0.22827200	3.03370500	2.60810000
H	-3.55462700	-3.88092600	1.67895000
H	-2.59208500	-0.20211100	3.73286500
H	-6.15637300	-3.96080600	1.43220800
H	-7.34445200	-2.43288400	3.58538300
H	-5.92257600	0.06606400	4.93111900
H	5.47409600	2.51122800	-1.70447600
H	3.49940100	-4.14195500	-2.41690100
H	6.86628800	-4.96037100	-3.16432600
H	8.53670600	-2.83195200	-1.52872400
H	5.58256200	1.76544400	1.91662000
H	6.96296300	1.30352400	2.89175900
H	7.53725000	-1.16297700	2.17370300
H	9.10105800	0.85285500	0.24338200
H	-6.33050800	-4.65000500	-2.74459400
H	-6.51123700	-2.92469100	-3.18629400
H	-4.90523900	-3.64253900	-3.12824600
H	6.44749000	3.75103300	3.87712800
H	8.18204800	3.50447200	3.71707700
H	7.44770200	4.99740600	3.06353200

Cartesian coordinates of the low-energy conformer of acetonyl amariin [**1a (^{O,3}B)-1**] at the B3LYP/6-31G(d,p) level in acetone (PCM).

C	-2.01068600	-1.40417400	1.46833200
O	-1.52728300	-1.60861800	2.55947300
C	-1.92308000	-3.58852900	-1.63132700
O	-1.00750200	-4.38390700	-1.75710400
C	-3.47174800	-1.18197100	1.19744400
C	-4.18872800	-0.72961900	2.24723600
C	-5.58015400	-0.28288800	2.10476500
C	-6.02240800	0.05991500	0.68091900
C	-5.57076600	-1.11632100	-0.21160200
C	-4.05465500	-1.43443600	-0.19409100
C	-4.10216400	-2.94089000	-0.44695600
C	-5.35295400	-3.35654300	0.00432300
C	-5.74412700	-4.68671500	0.04678000
C	-4.81104900	-5.64912400	-0.38487600
C	-3.57204400	-5.25291100	-0.88377900
C	-3.20489200	-3.89576600	-0.94400500
O	-5.13629100	-6.96893600	-0.36176400
O	-6.95955400	-5.12973800	0.46835200
O	-6.36112400	-0.15891900	3.03938400
C	-5.36761800	1.36103800	0.14834400
O	-5.99086500	-0.95609300	-1.52045700
O	-6.18230400	-2.32151300	0.36423700
H	-3.51437600	-0.87962800	-0.96061500
O	-7.42462500	0.18149900	0.60822900
C	0.22407900	-1.07291900	-2.10991600
C	0.74872000	-1.72534100	-0.80658500
C	0.12263900	-1.06294400	0.42823200
C	0.41961300	0.45061800	0.42635300
C	0.31548300	1.03450800	-0.99773600
O	-0.42369700	0.17776700	-1.84722400
O	2.17267900	-1.43953800	-0.77413400
O	1.77068700	0.66514400	0.87225700
C	-0.74194500	-1.94115500	-2.90724500
O	-1.30116800	-1.23793500	0.33038100
O	-0.41085200	2.24142300	-0.90575600
C	-0.11057900	3.23319000	-1.81027600
O	0.74794400	3.09898800	-2.66083500
C	-0.92681100	4.44580300	-1.59469900
C	-0.63030300	5.56782900	-2.38366100
C	-1.35534200	6.74200500	-2.21068800
C	-2.37715300	6.79145300	-1.25303100
C	-2.67823300	5.66712700	-0.46889600
C	-1.95187800	4.49005900	-0.63287400
O	-1.07585400	7.83851500	-2.97243200
O	-3.06677900	7.95905700	-1.12027500
O	-3.68920000	5.85668000	0.42572300
O	-1.90858500	-2.34196600	-2.16010200
C	2.93803000	-2.09853100	0.12411900
O	2.54961400	-3.01332800	0.81736100
C	1.98321200	0.71373600	2.21632700
O	1.07522100	0.76892300	3.02349700
C	4.34371000	-1.57374300	0.07537700
C	5.32218500	-2.48466400	-0.08465800
C	6.71382700	-2.06138800	-0.33398200
C	6.84566300	-0.69068900	-1.01214100
C	6.07849500	0.28956000	-0.08979000
C	4.59159300	-0.06837000	0.20331300
C	4.47469800	0.38819100	1.66786300
C	5.77938600	0.46313500	2.14735500
C	6.10806700	0.79963300	3.45357600

C	5.05211200	1.06875800	4.34184800
C	3.73782900	1.02050200	3.88909000
C	3.42548400	0.69317300	2.55595600
O	5.31188600	1.40258700	5.63385400
O	7.37869500	0.88926000	3.92885700
O	7.70533900	-2.67507300	0.02803900
C	6.27159100	-0.62379300	-2.44106600
O	6.13993300	1.59564000	-0.55170900
O	6.74232900	0.19991500	1.20941400
H	3.93464100	0.48804600	-0.46533300
O	8.20274500	-0.31205300	-1.07095100
C	-5.50481900	2.63611100	0.96796900
O	-4.50309800	3.33008400	1.11666000
C	-6.84275000	3.04612000	1.51697200
C	6.75754400	-1.75135200	-3.34334400
C	6.99364300	-1.41371300	-4.79314200
O	6.90852400	-2.87826300	-2.89844500
H	-3.73741400	-0.66383500	3.23172400
H	-2.89311900	-6.00676800	-1.26333500
H	-6.03757500	-7.05776900	-0.01340500
H	-7.53057000	-4.37584100	0.67918100
H	-4.30078400	1.22122700	-0.02780800
H	-5.83707200	1.55412300	-0.82441400
H	-6.94128200	-0.75217600	-1.48573100
H	-7.77284200	-0.07305200	1.48134800
H	1.09579500	-0.90500300	-2.75957100
H	0.59911900	-2.80524600	-0.79714000
H	0.48411700	-1.52034800	1.34882100
H	-0.27346200	0.95785600	1.09830200
H	1.31042900	1.22576500	-1.41397400
H	-1.12053000	-1.36226800	-3.75087500
H	-0.21874300	-2.82726100	-3.26978800
H	0.16072300	5.52428300	-3.12226400
H	-2.19281200	3.62350700	-0.03143200
H	-1.68654100	8.54705100	-2.71571500
H	-3.75919000	7.82031700	-0.45318900
H	-3.98846400	5.00504700	0.80710600
H	5.10814900	-3.54842200	-0.06238400
H	2.93417800	1.25289000	4.57683200
H	6.27321500	1.40316500	5.76481500
H	8.00921400	0.73878100	3.20813900
H	5.17677800	-0.68717100	-2.43357800
H	6.52156400	0.35340900	-2.86028800
H	7.07747600	1.78477200	-0.72790600
H	8.68122600	-0.97083400	-0.53304100
H	-7.14800600	2.33929100	2.29611300
H	-6.77877400	4.05005400	1.93781000
H	-7.60837000	2.99942200	0.73785800
H	7.85282900	-0.73646800	-4.86792600
H	7.18885800	-2.31894000	-5.36896400
H	6.13279000	-0.87863300	-5.20839100

Cartesian coordinates of the low-energy conformer of acetonyl amariin [**1a (^{O,3}B)-2**] at the B3LYP/6-31G(d,p) level in acetone (PCM).

C	-2.01083100	-1.40486600	1.46816100
O	-1.52735500	-1.61011900	2.55912000
C	-1.92386600	-3.58796700	-1.63226900
O	-1.00845600	-4.38349100	-1.75824900
C	-3.47187200	-1.18228700	1.19753700
C	-4.18873000	-0.73038400	2.24761300
C	-5.58003500	-0.28319300	2.10539100
C	-6.02208900	0.06061600	0.68171800
C	-5.57086600	-1.11530600	-0.21142500
C	-4.05486700	-1.43390200	-0.19410900
C	-4.10281300	-2.94021400	-0.44765200
C	-5.35371500	-3.35570800	0.00347800
C	-5.74522200	-4.68580300	0.04538800
C	-4.81241000	-5.64827300	-0.38667100
C	-3.57329500	-5.25219000	-0.88541800
C	-3.20579600	-3.89512200	-0.94511300
O	-5.13800000	-6.96801000	-0.36411200
O	-6.96076800	-5.12868700	0.46677900
O	-6.36098600	-0.15950600	3.04005700
C	-5.36673300	1.36173100	0.14998600
O	-5.99099400	-0.95434500	-1.52016600
O	-6.18276300	-2.32063300	0.36388900
H	-3.51443500	-0.87889500	-0.96038300
O	-7.42426900	0.18275700	0.60898700
C	0.22377800	-1.07259700	-2.11018000
C	0.74836000	-1.72534400	-0.80698600
C	0.12239500	-1.06315000	0.42799100
C	0.41952400	0.45041700	0.42642800
C	0.31567200	1.03456700	-0.99757100
O	-0.42380100	0.17812900	-1.84717700
O	2.17235800	-1.43964500	-0.77447600
O	1.77054300	0.66467200	0.87254600
C	-0.74235800	-1.94050100	-2.90772300
O	-1.30143600	-1.23803000	0.33022900
O	-0.41019000	2.24174300	-0.90557500
C	-0.10933100	3.23354500	-1.80985700
O	0.74956900	3.09930600	-2.66002900
C	-0.92537100	4.44632700	-1.59447300
C	-0.62799300	5.56847400	-2.38291400
C	-1.35287400	6.74278800	-2.21017200
C	-2.37541400	6.79222800	-1.25331200
C	-2.67739100	5.66776100	-0.46971200
C	-1.95120200	4.49055100	-0.63344900
O	-1.07247100	7.83944800	-2.97136400
O	-3.06484900	7.95997700	-1.12076700
O	-3.68910500	5.85732500	0.42402200
O	-1.90911500	-2.34126600	-2.16074300
C	2.93768400	-2.09893500	0.12358700
O	2.54926800	-3.01393200	0.81655400
C	1.98294900	0.71281700	2.21663600
O	1.07487600	0.76787000	3.02373100
C	4.34337900	-1.57418600	0.07496200
C	5.32176800	-2.48511500	-0.08556600
C	6.71347700	-2.06189300	-0.33466700
C	6.84544500	-0.69085800	-1.01208000
C	6.07834700	0.28897900	-0.08922200
C	4.59139100	-0.06888800	0.20360700
C	4.47443400	0.38697100	1.66835400
C	5.77909800	0.46141400	2.14801400
C	6.10767400	0.79730600	3.45442400

C	5.05168100	1.06629600	4.34267500
C	3.73743300	1.01850600	3.88975100
C	3.42517900	0.69182700	2.55644600
O	5.31137300	1.39955000	5.63484500
O	7.37826900	0.88649500	3.92988800
O	7.70486200	-2.67588500	0.02712900
C	6.27146000	-0.62303700	-2.44096700
O	6.14012300	1.59529400	-0.55032500
O	6.74208100	0.19842800	1.21007400
H	3.93453100	0.48790300	-0.46482000
O	8.20256800	-0.31227200	-1.07070600
C	-5.50372100	2.63664400	0.96991000
O	-4.50211200	3.33099300	1.11765200
C	-6.84127400	3.04617700	1.52015600
C	6.75678900	-1.75037000	-3.34382700
C	6.99168800	-1.41232800	-4.79377000
O	6.90824100	-2.87737800	-2.89937300
H	-3.73737700	-0.66525300	3.23212900
H	-2.89457900	-6.00608900	-1.26526700
H	-6.03921300	-7.05676300	-0.01554500
H	-7.53144700	-4.37473300	0.67831500
H	-4.29989600	1.22164800	-0.02595800
H	-5.83577000	1.55552400	-0.82284500
H	-6.94133800	-0.75010500	-1.48529200
H	-7.77262000	-0.07209300	1.48196700
H	1.09551800	-0.90464300	-2.75979600
H	0.59867600	-2.80524200	-0.79777500
H	0.48387700	-1.52075000	1.34848400
H	-0.27362000	0.95758800	1.09836600
H	1.31071800	1.22544200	-1.41374300
H	-1.12078100	-1.36138600	-3.75127100
H	-0.21929500	-2.82663300	-3.27040900
H	0.16358800	5.52494500	-3.12092400
H	-2.19279400	3.62391500	-0.03239200
H	-1.68313600	8.54808900	-2.71488200
H	-3.75749800	7.82140500	-0.45389600
H	-3.98761600	5.00590400	0.80648500
H	5.10760100	-3.54886100	-0.06375900
H	2.93375200	1.25079400	4.57749300
H	6.27269100	1.39987500	5.76590500
H	8.00883000	0.73634700	3.20913900
H	5.17660700	-0.68574800	-2.43359900
H	6.52196600	0.35423000	-2.85974300
H	7.07770800	1.78421200	-0.72656000
H	8.68096900	-0.97091000	-0.53257600
H	-7.60756100	2.99925400	0.74171600
H	-7.14562200	2.33920400	2.29952700
H	-6.77723700	4.05010500	1.94100100
H	7.85150300	-0.73595200	-4.86922000
H	7.18545100	-2.31752600	-5.37013300
H	6.13094800	-0.87624500	-5.20793600

Cartesian coordinates of the low-energy conformer of acetonyl amariin [**1a (^{O,3}B)-3**] at the B3LYP/6-31G(d,p) level in acetone (PCM).

C	-2.05011300	-1.42626500	1.44610300
O	-1.57796400	-1.65733200	2.53683300
C	-1.96139700	-3.56521400	-1.68091800
O	-1.05323700	-4.36874700	-1.80885200
C	-3.50609200	-1.18241000	1.16707600
C	-4.22648300	-0.73716100	2.21754000
C	-5.61087700	-0.27058700	2.06969500
C	-6.03601200	0.09899700	0.64741500
C	-5.59167200	-1.06961400	-0.25866000
C	-4.07985200	-1.40682100	-0.23296000
C	-4.14357100	-2.90868200	-0.50812300
C	-5.40340300	-3.31533500	-0.07419500
C	-5.81100800	-4.64114500	-0.05479700
C	-4.88549200	-5.60861300	-0.49152100
C	-3.63703600	-5.22053400	-0.97295000
C	-3.25308500	-3.86722300	-1.01019500
O	-5.22680800	-6.92453800	-0.49050600
O	-7.03563900	-5.07542300	0.34880600
O	-6.39844300	-0.15049100	2.99925800
C	-5.35925600	1.39905900	0.14046500
O	-5.99823700	-0.88488800	-1.56856600
O	-6.22310600	-2.27549000	0.29377200
H	-3.52609200	-0.84760400	-0.98655300
O	-7.43584400	0.23985500	0.56419000
C	0.21786100	-1.06709300	-2.10701800
C	0.72537400	-1.74059300	-0.80721300
C	0.09500900	-1.08655100	0.42994800
C	0.40741700	0.42389000	0.44935400
C	0.32454100	1.02566100	-0.96842500
O	-0.41754900	0.18765400	-1.83428500
O	2.15210900	-1.46891200	-0.76414800
O	1.75536000	0.61635700	0.91418800
C	-0.75057100	-1.91441100	-2.92356000
O	-1.32952900	-1.24541400	0.31719400
O	-0.38807700	2.23992700	-0.86867800
C	-0.06782500	3.23944800	-1.75764900
O	0.79706100	3.10602600	-2.60188000
C	-0.87214500	4.45845900	-1.53377000
C	-0.55563800	5.58724300	-2.30508700
C	-1.26900900	6.76725900	-2.12335200
C	-2.29930700	6.81576500	-1.17478100
C	-2.62040600	5.68473500	-0.40841000
C	-1.90575800	4.50178600	-0.58105400
O	-0.96967700	7.87040600	-2.86774800
O	-2.97699100	7.98925200	-1.03294600
O	-3.63795000	5.87381400	0.47876100
O	-1.92825900	-2.31180700	-2.19245700
C	2.90835000	-2.12919000	0.14046600
O	2.51130500	-3.03388700	0.84138500
C	1.94972500	0.64440900	2.26224800
O	1.03125500	0.71602200	3.05616200
C	4.31856500	-1.61433000	0.08521600
C	5.28778600	-2.52404500	-0.13125500
C	6.67887900	-2.13976100	-0.46109200
C	6.84251800	-0.69065400	-0.95986600
C	6.06866900	0.22423100	0.00971700
C	4.57469800	-0.11963900	0.26018200
C	4.44067400	0.27395300	1.74163200
C	5.73798200	0.27894400	2.24635100
C	6.05250500	0.54253300	3.57269900

C	4.98918800	0.80860500	4.45324000
C	3.68286500	0.82754400	3.97574900
C	3.38455200	0.57113500	2.62385400
O	5.23525500	1.07409100	5.76362500
O	7.31579600	0.56435200	4.07424600
O	7.60744100	-2.92883400	-0.43435900
C	6.27918800	-0.55875800	-2.40137800
O	6.16773900	1.55631100	-0.36089400
O	6.70682900	0.02801000	1.31298900
H	3.93336200	0.46695400	-0.39766900
O	8.17918000	-0.24838300	-0.90470800
C	-5.48809400	2.66454000	0.97624600
O	-4.48059300	3.34726900	1.13737800
C	-6.82430700	3.08022400	1.52518500
C	7.13242400	-1.22989700	-3.47189900
C	6.43779600	-1.60258600	-4.75412700
O	8.33091400	-1.43289700	-3.30674300
H	-3.78305600	-0.69180100	3.20676000
H	-2.96375100	-5.97720300	-1.35696500
H	-6.13210800	-7.00744000	-0.15122100
H	-7.60010400	-4.31777100	0.56384300
H	-4.29257900	1.24770500	-0.02705500
H	-5.81616900	1.61249600	-0.83405100
H	-6.94650100	-0.67035700	-1.53912200
H	-7.79503400	-0.02163900	1.43080900
H	1.09671000	-0.90119900	-2.74749300
H	0.56500500	-2.81912500	-0.81180900
H	0.44308900	-1.55913600	1.34790500
H	-0.28804100	0.93028500	1.11940900
H	1.32544800	1.20940200	-1.37363600
H	-1.11489900	-1.32073800	-3.76320000
H	-0.23383900	-2.80159500	-3.29270500
H	0.24192400	5.54444000	-3.03666900
H	-2.16220200	3.63031200	0.00670500
H	-1.57406500	8.58282900	-2.60690900
H	-3.67613100	7.85011600	-0.37300400
H	-3.94760000	5.02106600	0.84915300
H	5.05794000	-3.58546000	-0.14589800
H	2.87451500	1.05510300	4.65955700
H	6.19321100	1.03289500	5.91179600
H	7.95453900	0.42397900	3.35859200
H	5.25116600	-0.92299700	-2.47271200
H	6.24400400	0.50985800	-2.65106600
H	7.10745000	1.70883800	-0.56501400
H	8.62521800	-0.68250400	-1.66063300
H	-7.58762700	3.04818500	0.74312600
H	-7.13817300	2.36796100	2.29596300
H	-6.75301200	4.07901000	1.95695300
H	5.74693300	-2.43213900	-4.56208500
H	5.83494400	-0.76577800	-5.12194600
H	7.16783100	-1.90322100	-5.50606500

Cartesian coordinates of the low-energy conformer of acetonyl amariin [**1a (^{O,3}B)-4**] at the B3LYP/6-31G(d,p) level in acetone (PCM).

C	-1.78464900	-1.88386600	1.33177500
O	-1.16437800	-2.60838500	2.07709700
C	-2.05769300	-2.95613900	-2.15367900
O	-1.18764200	-3.73163600	-2.51117400
C	-3.27165200	-1.67966700	1.36720200
C	-3.84902800	-1.80413600	2.58032200
C	-5.24100600	-1.39740900	2.81335800
C	-5.82826500	-0.42037400	1.78314200
C	-5.52488000	-1.01826500	0.39473400
C	-4.03405200	-1.33981300	0.08935200
C	-4.18786800	-2.62728000	-0.73459300
C	-5.43866700	-3.14041500	-0.40194500
C	-5.91481400	-4.36837800	-0.83835500
C	-5.06152100	-5.14185600	-1.64375700
C	-3.81045700	-4.64883500	-2.00622900
C	-3.36036000	-3.38573600	-1.57916400
O	-5.46550700	-6.35690700	-2.09906300
O	-7.13990900	-4.87780500	-0.53676300
O	-5.91394700	-1.73999400	3.77499400
C	-5.17603400	0.97469800	2.00799500
O	-6.03589900	-0.24212100	-0.63800100
O	-6.18376800	-2.32132800	0.40552400
H	-3.55966900	-0.52101200	-0.45258200
O	-7.22481400	-0.34846700	1.94238600
C	0.21272100	-0.45958000	-2.09937800
C	0.74719500	-1.36122100	-0.96088200
C	0.20186500	-0.91688300	0.40513100
C	0.53174600	0.56901300	0.66287400
C	0.42327000	1.40066800	-0.62882400
O	-0.38147100	0.73244600	-1.58143600
O	2.18310500	-1.13673700	-0.92707700
O	1.90003100	0.66757800	1.10159200
C	-0.81448900	-1.11112600	-3.01193400
O	-1.22975700	-1.06010600	0.41087800
O	-0.25679600	2.60259300	-0.34811900
C	0.11278700	3.71952300	-1.06965300
O	1.18404000	3.80126200	-1.63797300
C	-0.93385000	4.75808900	-1.05222400
C	-0.59639900	6.04116800	-1.50945400
C	-1.57546200	7.02805400	-1.56207700
C	-2.89010200	6.72440400	-1.18144300
C	-3.22828200	5.43674400	-0.73775500
C	-2.24711800	4.45154000	-0.65817200
O	-1.26039000	8.28327500	-1.99382000
O	-3.82085500	7.71667800	-1.26720800
O	-4.54024900	5.26435100	-0.42115400
O	-1.96953600	-1.61415100	-2.30493300
C	2.94900800	-1.99000800	-0.21002800
O	2.54674100	-3.01840900	0.28898500
C	2.15256300	0.39084700	2.40908300
O	1.26808500	0.25750800	3.23411200
C	4.37027800	-1.50768400	-0.19257500
C	5.31366200	-2.40512800	-0.53549800
C	6.71325700	-1.98500600	-0.74432400
C	6.87175300	-0.52238200	-1.18367200
C	6.16294500	0.30536700	-0.08228400
C	4.67410100	-0.05804700	0.19612500
C	4.61620500	0.12790400	1.72229400
C	5.93651800	0.08320100	2.16112900
C	6.31357600	0.15830900	3.49524100

C	5.29292700	0.27416800	4.45498100
C	3.96428700	0.33757200	4.04823500
C	3.60330000	0.27816100	2.68881900
O	5.60143600	0.35029300	5.77678600
O	7.59973300	0.12788300	3.93518400
O	7.69290800	-2.67712800	-0.51675500
C	6.26308000	-0.19873100	-2.56187500
O	6.25371000	1.66901500	-0.31544500
O	6.86265800	-0.02528500	1.15816100
H	4.01784200	0.62894100	-0.33812100
O	8.23858900	-0.17738000	-1.22121900
C	-5.60487000	2.11636600	1.09390400
O	-4.74877300	2.67785000	0.41543400
C	-7.02228700	2.61660600	1.14826800
C	6.68032400	-1.18024200	-3.65015500
C	6.89810000	-0.61993600	-5.03202400
O	6.79302100	-2.36985000	-3.39872600
H	-3.27663200	-2.16153900	3.42998300
H	-3.18237100	-5.24358200	-2.65789600
H	-6.35838700	-6.53101600	-1.76144600
H	-7.64406500	-4.23639500	-0.01390700
H	-5.43627800	1.26851600	3.03275500
H	-4.08927500	0.90790500	1.94246000
H	-6.99410100	-0.17125100	-0.49215800
H	-7.42357100	-0.84703200	2.75814100
H	1.07230100	-0.19839800	-2.73461500
H	0.55143200	-2.41899800	-1.14249500
H	0.61764700	-1.53772100	1.19812500
H	-0.13703500	0.95831300	1.43133900
H	1.41728300	1.61589600	-1.03514000
H	-1.19706400	-0.35613400	-3.70060400
H	-0.34912700	-1.92413900	-3.56962200
H	0.41639000	6.26589700	-1.82131500
H	-2.50648400	3.45836400	-0.31650400
H	-2.06733200	8.82103400	-1.97036100
H	-4.67539300	7.34687900	-0.99093600
H	-4.71443700	4.32712100	-0.17063800
H	5.06295100	-3.44836400	-0.69858500
H	3.18883700	0.44858800	4.79607400
H	6.56602300	0.30498100	5.87098800
H	8.20468600	0.09322300	3.17867300
H	5.16724900	-0.22407500	-2.52906700
H	6.54144500	0.82464600	-2.82371100
H	7.19141300	1.85951200	-0.48929100
H	8.70864000	-0.92649800	-0.80878700
H	-7.73287500	1.79388500	1.23528800
H	-7.12943600	3.22478500	2.05591400
H	-7.23737700	3.24220700	0.28089600
H	6.04525400	-0.00243700	-5.33425000
H	7.77537200	0.03767500	-5.01865000
H	7.05324200	-1.42568400	-5.75017400

Cartesian coordinates of the low-energy conformer of acetonyl amariin [**1a (^{O,3}B)-5**] at the B3LYP/6-31G(d,p) level in acetone (PCM).

C	-1.77735400	-1.91174400	1.33935200
O	-1.15578400	-2.64168800	2.07790600
C	-2.07532500	-3.00120200	-2.11979800
O	-1.21079400	-3.79254100	-2.45375700
C	-3.26281600	-1.70010000	1.38251100
C	-3.83540400	-1.80674000	2.59780600
C	-5.21696000	-1.37390700	2.83178200
C	-5.81817000	-0.39466600	1.80305000
C	-5.52145700	-0.97624300	0.40724600
C	-4.02770800	-1.34779300	0.11334900
C	-4.21092100	-2.62355700	-0.72110600
C	-5.48082000	-3.10114000	-0.40649600
C	-5.98219600	-4.31738600	-0.85110600
C	-5.13728900	-5.11391500	-1.64155800
C	-3.86812700	-4.65368100	-1.98850300
C	-3.39216800	-3.40332600	-1.55654700
O	-5.56493000	-6.31834900	-2.10163400
O	-7.22512000	-4.79235700	-0.56805200
O	-5.87575000	-1.68440600	3.81428300
C	-5.15794400	0.99629500	2.02565400
O	-6.05262400	-0.11228100	-0.53843800
O	-6.22142800	-2.26021400	0.37837400
H	-3.52774900	-0.53741300	-0.41990900
O	-7.20635000	-0.32851800	2.00354200
C	0.21460100	-0.51420500	-2.09898600
C	0.75252700	-1.39763100	-0.94790300
C	0.20532400	-0.93629900	0.41150000
C	0.52769900	0.55475300	0.64838500
C	0.41693100	1.36719300	-0.65536300
O	-0.38534700	0.68245700	-1.59841300
O	2.18743600	-1.16728600	-0.91702900
O	1.89503300	0.66582500	1.08687000
C	-0.80860000	-1.18254300	-3.00347200
O	-1.22544000	-1.08457800	0.41947700
O	-0.26478600	2.57258200	-0.39233200
C	0.10278500	3.67667500	-1.13440000
O	1.16143000	3.73670900	-1.72877700
C	-0.92682900	4.73215300	-1.10504900
C	-0.58015800	5.99848400	-1.60080600
C	-1.54054000	7.00393500	-1.64253600
C	-2.84681300	6.73514900	-1.21132600
C	-3.19517800	5.46472100	-0.72696600
C	-2.23171600	4.46079000	-0.65962000
O	-1.21552900	8.24287000	-2.11235700
O	-3.76094300	7.74336800	-1.28780100
O	-4.49840400	5.32991100	-0.36124000
O	-1.97164600	-1.66278200	-2.29342600
C	2.95628000	-2.00853600	-0.18866900
O	2.55757800	-3.03250600	0.32211100
C	2.14735400	0.40737100	2.39827500
O	1.26242300	0.28147200	3.22398500
C	4.37543900	-1.51998000	-0.17489100
C	5.32298600	-2.41861000	-0.50296000
C	6.72139400	-1.99610800	-0.71519900
C	6.87470700	-0.53949200	-1.17598600
C	6.16086700	0.30141800	-0.08778800
C	4.67298200	-0.06375600	0.19395200
C	4.61251600	0.14369800	1.71739000
C	5.93243600	0.11039600	2.15833600
C	6.30791800	0.20609700	3.49158100

C	5.28592800	0.33149900	4.44863600
C	3.95749400	0.38395100	4.03962500
C	3.59813700	0.30396500	2.68082900
O	5.59267200	0.42773300	5.76951500
O	7.59386800	0.18733000	3.93269600
O	7.70335800	-2.68058300	-0.47493500
C	6.26685300	-0.23864600	-2.55963600
O	6.24667900	1.66184000	-0.34066600
O	6.86028000	-0.00849300	1.15820600
H	4.01477000	0.61310600	-0.35073100
O	8.24018500	-0.18959200	-1.21648400
C	-5.60507700	2.14707400	1.13132300
O	-4.73917300	2.77768100	0.52939700
C	-7.05041400	2.55495800	1.10134700
C	6.68554300	-1.23666700	-3.63214600
C	6.89726100	-0.69895600	-5.02391100
O	6.80388800	-2.42142200	-3.36110300
H	-3.26606600	-2.16345600	3.44979600
H	-3.24813300	-5.26522000	-2.63240500
H	-6.46645300	-6.47041500	-1.77638500
H	-7.71852400	-4.13651300	-0.05259000
H	-5.39472500	1.28428600	3.05789300
H	-4.07194600	0.93326100	1.93918300
H	-5.76571000	-0.37309000	-1.42704300
H	-7.36171700	-0.77161400	2.85998500
H	1.07354700	-0.25858100	-2.73734900
H	0.56015600	-2.45858300	-1.11454700
H	0.62342400	-1.54437300	1.21316400
H	-0.14373100	0.95137600	1.41078800
H	1.41035100	1.57838700	-1.06488400
H	-1.18258500	-0.44232400	-3.71269400
H	-0.34330300	-2.01058300	-3.53876900
H	0.42585200	6.19570400	-1.95068000
H	-2.49888300	3.48139300	-0.28563800
H	-2.00934900	8.79869500	-2.06973100
H	-4.61149500	7.39638500	-0.97209800
H	-4.68665000	4.40361800	-0.07975100
H	5.07652200	-3.46500700	-0.65157600
H	3.18083400	0.50266300	4.78502200
H	6.55733900	0.38798800	5.86538700
H	8.19932700	0.14505500	3.17695700
H	5.17100600	-0.26451600	-2.52735400
H	6.54413600	0.78075900	-2.83774200
H	7.18393000	1.85341500	-0.51577900
H	8.71246600	-0.93010600	-0.79126500
H	-7.19580000	3.37066800	0.39207500
H	-7.67707100	1.69830600	0.84796800
H	-7.35306000	2.88088000	2.10318100
H	7.77326500	-0.03950400	-5.02452300
H	7.05174600	-1.51627900	-5.72901200
H	6.04216300	-0.08828100	-5.33349500

Cartesian coordinates of the low-energy conformer of acetonyl amariin [**1a (^{O,3}B)-6**] at the B3LYP/6-31G(d,p) level in acetone (PCM).

C	-1.99046800	-1.53807500	1.38931700
O	-1.45897600	-1.97872500	2.38450300
C	-1.99604900	-3.32520300	-1.90895100
O	-1.09555400	-4.12519300	-2.09935400
C	-3.46592000	-1.32615200	1.22001700
C	-4.15757100	-1.11902500	2.35861100
C	-5.58185400	-0.73865400	2.33100300
C	-6.02615700	-0.02432200	1.04946300
C	-5.60828700	-0.97554200	-0.09715700
C	-4.09908500	-1.33448500	-0.17433500
C	-4.18719200	-2.77769100	-0.68265500
C	-5.45541900	-3.22772200	-0.32454800
C	-5.88495000	-4.53565500	-0.50029200
C	-4.97211900	-5.44723200	-1.06057400
C	-3.71023700	-5.01528600	-1.46114700
C	-3.30530100	-3.67659200	-1.30129400
O	-5.33324200	-6.74440300	-1.25192500
O	-7.12144100	-4.99853900	-0.16915100
O	-6.39356000	-1.03160800	3.19548600
C	-5.39897200	1.36922200	0.84573200
O	-6.03351600	-0.51794700	-1.33270500
O	-6.26228700	-2.25580900	0.20636400
H	-3.57036800	-0.66578900	-0.85373000
O	-7.42959800	0.12392300	1.04189600
C	0.23179600	-0.83347400	-2.15706800
C	0.71250300	-1.57399400	-0.88480100
C	0.10491900	-0.95035000	0.38079300
C	0.45436000	0.55169700	0.45581500
C	0.41236800	1.19843300	-0.94192100
O	-0.37014200	0.42259600	-1.82703800
O	2.14684900	-1.34552000	-0.82254900
O	1.80246600	0.69430700	0.93936600
C	-0.75795500	-1.60838100	-3.01483600
O	-1.32511600	-1.07520200	0.30289800
O	-0.23463300	2.44675300	-0.82293800
C	0.05486000	3.39051400	-1.77846600
O	0.90126600	3.21701700	-2.63362200
C	-0.77108400	4.60553900	-1.62425100
C	-1.77569000	4.69761300	-0.65010700
C	-2.53794800	5.85833600	-0.56051800
C	-2.29662100	6.92481700	-1.43659600
C	-1.28855600	6.82669900	-2.40741900
C	-0.52559500	5.67059400	-2.50703000
O	-3.52152100	5.95439000	0.37980800
O	-3.07228500	8.03596300	-1.30555400
O	-1.15468900	7.93437600	-3.19813400
O	-1.93632400	-2.02891200	-2.29691000
C	2.88035400	-2.08975900	0.03473700
O	2.45485600	-3.03581400	0.66082900
C	1.99260700	0.61876200	2.28483900
O	1.07201100	0.63386500	3.07977000
C	4.30327300	-1.61040200	0.03230000
C	5.25336100	-2.54182300	-0.17467600
C	6.66095000	-2.15052200	-0.38439200
C	6.84417800	-0.74411700	-0.97205800
C	6.10069100	0.19909600	0.00628600
C	4.59890800	-0.12495600	0.25846100
C	4.47783800	0.23919600	1.74813500
C	5.77744900	0.23328600	2.24686400
C	6.09867400	0.46634900	3.57727300

C	5.03982300	0.71013200	4.46962500
C	3.73120700	0.74127100	3.99903600
C	3.42729200	0.52025900	2.64233200
O	5.29241600	0.94306100	5.78490200
O	7.36443200	0.47733300	4.07352200
O	7.62816000	-2.81845600	-0.05420100
C	6.28508900	-0.56631100	-2.39713500
O	6.21432700	1.52997500	-0.36625200
O	6.74334800	-0.00093500	1.30409300
H	3.97005200	0.49506700	-0.38057300
O	8.21340200	-0.40775500	-0.99583800
C	-5.58051300	2.27515100	2.06067000
O	-5.38904800	1.83251600	3.18268400
C	-5.95710500	3.71112800	1.80811700
C	6.73615200	-1.65104000	-3.36709900
C	6.97697300	-1.23323400	-4.79519800
O	6.85549600	-2.80669000	-2.99193800
H	-3.67385000	-1.22522700	3.32393800
H	-3.04004100	-5.72107500	-1.93630200
H	-6.24281900	-6.86021400	-0.93464500
H	-7.66446100	-4.27151200	0.17063400
H	-4.31948000	1.29385700	0.67105400
H	-5.83645300	1.80973500	-0.05272600
H	-6.97342000	-0.28793700	-1.23342400
H	-7.75005900	-0.38184500	1.81254900
H	1.11803600	-0.66093500	-2.78561200
H	0.52053900	-2.64634200	-0.93219600
H	0.45865200	-1.46543900	1.27335200
H	-0.23894000	1.05211600	1.13287900
H	1.42417200	1.33467100	-1.33931700
H	-1.11698000	-0.95519300	-3.81169600
H	-0.26336200	-2.48100400	-3.44335600
H	-1.97274600	3.88001900	0.03069600
H	0.25395100	5.57939700	-3.25533800
H	-3.95757500	6.81536400	0.28108500
H	-2.80491400	8.68270200	-1.97784400
H	-0.46052400	7.79863000	-3.85774700
H	5.00410100	-3.59712400	-0.22065900
H	2.92639300	0.95356100	4.69195700
H	6.25077100	0.89472900	5.92816300
H	8.00001900	0.34556100	3.35366300
H	5.18863000	-0.58968800	-2.39962600
H	6.57340000	0.42558500	-2.75260700
H	7.16039900	1.69574000	-0.51902300
H	8.66445300	-1.11494900	-0.49706500
H	-6.91658100	3.74940000	1.27957100
H	-6.03029000	4.25311900	2.75188000
H	-5.21526100	4.19472100	1.16121400
H	7.85217400	-0.57381400	-4.83375000
H	7.14770900	-2.10710600	-5.42475000
H	6.12801900	-0.65389900	-5.17415300

Cartesian coordinates of the lowest-energy conformer of amariin [**1–A (^{O,3}B)**] at the B3LYP/6-31G(d,p) level in acetone (PCM).

C	0.05045800	-0.92487600	-2.39593100
C	0.67204700	-1.69930000	-1.20511200
C	0.31114400	-1.04865300	0.13907800
C	0.73538300	0.43878700	0.15680500
C	0.48655900	1.09825800	-1.22370700
O	-0.42867900	0.36451900	-1.99762600
O	2.10940000	-1.56516900	-1.36399300
O	2.15702700	0.46630900	0.38519200
C	-1.09801800	-1.62464800	-3.11143900
O	-1.11052400	-1.15593500	0.26486900
O	0.02514200	2.44099100	-1.14639900
C	-1.18510800	2.72822700	-0.59620600
O	-1.88314600	1.87741400	-0.05855300
C	-1.52758300	4.15754000	-0.71242400
C	-0.72507200	5.04406200	-1.45311300
C	-1.09266200	6.38136700	-1.53535400
C	-2.24730600	6.83999600	-0.88456900
C	-3.04245400	5.95277000	-0.14477000
C	-2.68359700	4.61201600	-0.05867600
O	-0.41034700	7.34665800	-2.22208400
O	-2.64086800	8.14048600	-0.93895800
O	-4.16005100	6.40131800	0.48978900
O	-2.25053800	-1.85184300	-2.27538900
C	2.90999600	-2.34838600	-0.61140500
O	2.53152200	-3.30590400	0.02861000
C	2.62016300	0.82239600	1.61403700
O	1.89807600	1.30183800	2.46686800
C	4.33824400	-1.89065800	-0.69922100
C	5.28413200	-2.84264400	-0.83435300
C	6.67853700	-2.47816300	-1.09209700
C	7.04298200	-0.98299000	-0.96073900
C	5.90570800	-0.08886200	-1.47201300
C	4.66162900	-0.39800800	-0.62058900
C	4.95729100	-0.02029400	0.83648600
C	6.27623400	-0.24736900	1.25003000
C	6.72671200	0.04107900	2.54244800
C	5.84123000	0.58759700	3.47583900
C	4.52901500	0.82863800	3.09479700
C	4.07025700	0.53318900	1.79995000
O	6.25528400	0.88426600	4.73628800
O	8.00814800	-0.17808100	2.93378100
O	7.55909300	-3.26905300	-1.40201400
O	8.20624600	-0.71034200	-1.64110800
O	7.26963000	-0.76422800	0.44806600
O	6.21491700	1.26809300	-1.31963900
O	5.62197500	-0.43372000	-2.80485300
H	3.83389900	0.18287500	-1.01514700
C	-1.64900900	-1.04494300	1.49850200
O	-1.00098700	-1.04825900	2.52408400
C	-2.40514700	-3.08895500	-1.74751300
O	-1.61365200	-3.99621800	-1.94318600
C	-3.13264400	-0.84904200	1.41526100
C	-3.78616100	-0.61727100	2.57281400
C	-5.19886700	-0.22245800	2.57687700
C	-5.94125800	-0.29791700	1.22783800
C	-5.00642000	0.14875300	0.08913900
C	-3.82139000	-0.83736000	0.05217000
C	-4.33433900	-2.24072300	-0.24834800
C	-5.55110800	-2.57352000	0.35606100
C	-6.10487500	-3.85440200	0.26951400

C	-5.42086500	-4.85510000	-0.42766300
C	-4.21098800	-4.54934300	-1.04056900
C	-3.67090900	-3.25499800	-0.97704600
O	-5.93652200	-6.11098800	-0.52081800
O	-7.29321800	-4.17757600	0.84761800
O	-5.80885900	0.14901400	3.57038400
O	-7.09447600	0.43996100	1.26207300
O	-6.31399600	-1.69670300	1.08658400
O	-5.64507400	0.09380800	-1.15798200
O	-4.61834600	1.45102800	0.43082200
H	-3.12330600	-0.50790000	-0.71369600
H	0.84802200	-0.80349200	-3.14420600
H	0.39846900	-2.75487500	-1.21575700
H	0.79395200	-1.57101700	0.96608500
H	0.22910400	0.96541400	0.96303200
H	1.44792600	1.18941300	-1.74323700
H	-1.45060500	-0.97607200	-3.91463500
H	-0.75026600	-2.57218700	-3.52480500
H	0.16594700	4.69040400	-1.95784600
H	-3.29926900	3.93431300	0.51940600
H	0.38173100	6.97545600	-2.63452300
H	-2.00051700	8.63820400	-1.47207200
H	-4.24430500	7.35333100	0.32333600
H	5.02697900	-3.89611700	-0.85932800
H	3.83717800	1.24940700	3.81314000
H	7.19400600	0.65172700	4.81332400
H	8.50692600	-0.53782300	2.18236200
H	8.67623600	-1.56230600	-1.73848600
H	7.10846600	1.41613600	-1.66757400
H	6.45810700	-0.41013200	-3.29770200
H	-3.25956800	-0.62828700	3.52086100
H	-3.68801800	-5.32025200	-1.59267900
H	-6.77842000	-6.13103300	-0.03915100
H	-7.64691800	-3.38846800	1.28890900
H	-7.30075200	0.57802200	2.20709700
H	-6.39579500	0.70629000	-1.10857300
H	-3.71828200	1.63272600	0.09374000

Cartesian coordinates of the lowest-energy conformer of amariin [**1–A ($^1C_4$)**] at the B3LYP/6-31G(d,p) level in acetone (PCM).

C	0.33519600	0.20567700	-2.12086500
C	0.82979800	-0.74996000	-1.03132300
C	0.61024600	-0.20644500	0.39336500
C	1.12861500	1.23394500	0.52391700
C	0.81104000	2.13579800	-0.67682300
O	0.89998100	1.51978900	-1.93524000
O	2.25821100	-0.79410700	-1.25194400
O	2.56862100	1.15784700	0.60652000
C	-1.19730400	0.29668800	-2.32419600
O	-0.79914700	-0.22487100	0.71550900
O	-0.50490500	2.65711200	-0.43655000
C	-0.75817400	3.93224000	-0.88272900
O	0.09705200	4.62398100	-1.40129700
C	-2.16394300	4.32696800	-0.66050900
C	-3.07251700	3.47561400	-0.00733700
C	-4.39233000	3.88687500	0.15337500
C	-4.79917500	5.14155400	-0.32604000
C	-3.88714600	5.99065700	-0.96693200
C	-2.56761600	5.58306300	-1.13572700
O	-5.37560400	3.16397800	0.75590300
O	-6.08255100	5.57752900	-0.18878500
O	-4.29170200	7.20765300	-1.42901200
O	-1.90724500	-0.80538700	-1.72305700
C	3.00155600	-1.78262400	-0.71865600
O	2.56282900	-2.79635500	-0.22103900
C	3.10057400	1.02370600	1.85469500
O	2.42363500	1.16008400	2.85709800
C	4.45744200	-1.44051900	-0.86711600
C	5.31208500	-2.43522400	-1.18073900
C	6.70640700	-2.14231600	-1.52552700
C	7.19450500	-0.69382900	-1.29678900
C	6.08273600	0.31845600	-1.61700300
C	4.89554100	0.01120100	-0.67625400
C	5.32756200	0.21660400	0.77698500
C	6.65558800	-0.13243300	1.04612200
C	7.22689300	-0.01771600	2.31768500
C	6.45228900	0.45494600	3.38108000
C	5.12555700	0.78869300	3.14916800
C	4.54948900	0.68077900	1.87123500
O	6.98456200	0.58437900	4.62571000
O	8.52051000	-0.34561500	2.56699100
O	7.49407700	-2.96064000	-1.97975600
O	8.33290300	-0.44642500	-2.01506000
O	7.53544800	-0.62192800	0.11105200
O	6.48776000	1.63588900	-1.38333700
O	5.76443600	0.09614300	-2.96682200
H	4.07685800	0.67968200	-0.93064400
C	-1.26140800	-1.41354300	1.17078800
O	-0.56072700	-2.38224000	1.36760900
C	-2.61263700	-1.65062700	-2.51384800
O	-2.47133400	-1.70841900	-3.72017400
C	-2.73447900	-1.40572800	1.46842000
C	-3.09288500	-1.90598100	2.66999300
C	-4.49129900	-1.91764700	3.09154100
C	-5.53579500	-1.58768200	2.00376900
C	-5.04009400	-0.46480700	1.08298900
C	-3.74385800	-0.95303000	0.41407500
C	-4.08914800	-2.16261900	-0.46052300
C	-5.05947400	-3.02199500	0.07015600

C	-5.47212200	-4.18639500	-0.58350700
C	-4.92423700	-4.50853400	-1.83050000
C	-3.99356300	-3.64992100	-2.40121000
C	-3.56375700	-2.49201000	-1.73551300
O	-5.31261100	-5.63254300	-2.48768900
O	-6.40743500	-5.02514700	-0.06738100
O	-4.88672300	-2.18849800	4.21693100
O	-6.73087000	-1.22672000	2.57928000
O	-5.71782800	-2.81065200	1.26350800
O	-5.96271500	-0.15773800	0.08557100
O	-4.74760600	0.66766200	1.89221000
H	-3.33215200	-0.14274500	-0.18133500
H	0.79468400	-0.13598800	-3.05091200
H	0.40175700	-1.74692300	-1.13633900
H	1.13114800	-0.84590500	1.10703000
H	0.73510900	1.68993900	1.43187400
H	1.51644800	2.96546200	-0.71397600
H	-1.57706200	1.19184100	-1.83810800
H	-1.42661500	0.32870100	-3.38869500
H	-2.74893200	2.51702900	0.37790900
H	-1.86015200	6.23201200	-1.63713500
H	-5.05455300	2.28140000	1.03495100
H	-6.59062800	4.88060700	0.25745500
H	-5.23535000	7.30871300	-1.22822800
H	4.97161700	-3.45958700	-1.28848600
H	4.51664100	1.13691500	3.97351600
H	7.91374300	0.30749900	4.59202000
H	8.93181900	-0.63968300	1.73782700
H	8.70890000	-1.32012000	-2.23926300
H	7.27120700	1.79130700	-1.93513900
H	5.04133600	0.69689800	-3.20335900
H	-2.35201100	-2.25675000	3.37991200
H	-3.59513500	-3.87468200	-3.38272900
H	-5.97196100	-6.09353300	-1.94528700
H	-6.71374800	-4.67136800	0.78299300
H	-6.72561200	-1.59752600	3.48474500
H	-6.83754700	-0.06103700	0.49512300
H	-5.45893300	0.74847200	2.55211500

Cartesian coordinates of the lowest-energy conformer of amariin [**1–B (^{O,3}B)**] at the B3LYP/6-31G(d,p) level in acetone (PCM).

C	3.00989600	-2.28029400	-0.21140200
O	2.63690400	-3.09921500	0.59906700
C	2.70671600	0.63246700	1.84998600
O	1.94425800	0.76203000	2.78929800
C	4.44146800	-1.94689800	-0.52877100
C	5.24015700	-2.98666800	-0.84095700
C	6.57390200	-2.78299900	-1.41574500
C	6.82743100	-1.38265200	-2.00015000
C	6.34636300	-0.31281900	-1.01049500
C	4.88631700	-0.48586600	-0.50249400
C	5.03766300	0.01595500	0.94541000
C	6.39945400	-0.04514500	1.22751500
C	6.95716900	0.27452000	2.45752100
C	6.08132900	0.67435500	3.48193800
C	4.71665700	0.76473600	3.22861000
C	4.17332100	0.45288000	1.96785000
O	6.56522000	0.99925700	4.70922400
O	8.28609000	0.22946000	2.73822700
O	7.42871900	-3.64828300	-1.52708100
O	8.18256500	-1.18366300	-2.24415800
O	6.46317300	0.96186900	-1.56654700
O	7.17836600	-0.43308500	0.16618900
H	4.20952300	0.12547100	-1.09951100
O	6.04380100	-1.31550600	-3.17017800
C	0.14901900	-0.97515300	-2.08052000
C	0.75886900	-1.65713700	-0.83147000
C	0.40856900	-0.88567700	0.45044600
C	0.85873600	0.58376200	0.34260200
C	0.60926100	1.13667800	-1.08410100
O	-0.31488000	0.34701600	-1.79307700
O	2.19715600	-1.57002700	-1.02149000
O	2.28447800	0.63986600	0.55654600
C	-0.98582800	-1.73310100	-2.73872600
O	-1.01037900	-0.85823400	0.61448100
O	0.14672800	2.48039400	-1.11649300
C	-1.05263500	2.80833000	-0.55959500
O	-1.72169300	2.00401600	0.07174600
C	-1.42206800	4.21555100	-0.79729300
C	-0.59964400	5.07947500	-1.53656700
C	-0.99291100	6.40001800	-1.73184100
C	-2.20356200	6.85532600	-1.19080500
C	-3.02251700	5.98600500	-0.45447200
C	-2.63843900	4.66689000	-0.25453500
O	-0.20159300	7.24667800	-2.44694000
O	-2.54072900	8.15453000	-1.41040200
O	-4.17353400	6.55602800	0.01360900
O	-2.08639100	-1.91552600	-1.82074200
C	-1.61346000	-1.80146000	1.36351900
O	-1.05632400	-2.73892200	1.89047100
C	-3.08783900	-2.68886900	-2.30773600
O	-2.99665200	-3.23544800	-3.39410200
C	-3.05657000	-1.42202700	1.51002100
C	-3.58252700	-1.48896100	2.75055200
C	-4.86717100	-0.85206700	3.04991800
C	-5.68295100	-0.33917200	1.84590400
C	-4.76045100	0.25679300	0.76656800
C	-3.81281300	-0.87304100	0.30143500
C	-4.62666600	-2.00704400	-0.31875300
C	-5.85972400	-2.25250200	0.29711700

C	-6.71731100	-3.27954700	-0.10809600
C	-6.35486400	-4.09272300	-1.18533500
C	-5.15695400	-3.84567500	-1.84077100
C	-4.28592200	-2.82204300	-1.42812700
O	-7.17067700	-5.09881700	-1.60264600
O	-7.90977000	-3.51620700	0.49962400
O	-5.32120900	-0.70228700	4.17662900
O	-6.63892600	0.55404800	2.25029600
O	-6.36374700	-1.51948500	1.34328300
O	-5.48502400	0.68990600	-0.35353900
O	-4.08409400	1.30330000	1.40155700
H	-3.10661500	-0.46060200	-0.41625200
H	4.88901100	-4.00914500	-0.74318200
H	4.05543600	1.09642800	4.01954100
H	7.52944200	0.89158100	4.69953600
H	8.78392800	-0.01210200	1.94241600
H	8.57864400	-2.07164400	-2.31626500
H	7.40051000	1.09451800	-1.78761300
H	6.04992500	-0.38672000	-3.45554500
H	0.95063300	-0.92488800	-2.83208600
H	0.46902800	-2.70568300	-0.74242300
H	0.86643600	-1.36545100	1.31646300
H	0.34808100	1.16804700	1.10448500
H	1.56867300	1.18271300	-1.61328500
H	-1.33752700	-1.17546200	-3.61005600
H	-0.62622400	-2.70755000	-3.07656600
H	0.33602400	4.73759200	-1.95912000
H	-3.26824400	3.98582900	0.30710300
H	-0.63394700	8.11458000	-2.47760800
H	-3.39387200	8.33166600	-0.98287000
H	-4.68589700	5.91737500	0.52823000
H	-3.01525700	-1.89164700	3.58296800
H	-4.88975700	-4.44936500	-2.69837000
H	-7.95684700	-5.10982100	-1.03432600
H	-8.02733000	-2.86650600	1.21165300
H	-6.74879200	0.41511100	3.21149300
H	-6.09160900	1.37878100	-0.03909400
H	-3.30486600	1.56214600	0.86854100

Cartesian coordinates of the lowest-energy conformer of amariin [**1–B ($^1C_4$)**] at the B3LYP/6-31G(d,p) level in acetone (PCM).

C	3.08096200	-1.63543600	-0.63096900
O	2.65808100	-2.62329400	-0.07067900
C	2.88279600	0.78207200	2.03483300
O	2.15478600	0.68049900	3.00524100
C	4.52671900	-1.28658100	-0.85102600
C	5.30484200	-2.26172000	-1.36058700
C	6.63466100	-1.96956900	-1.90627800
C	6.89085700	-0.48813800	-2.23541900
C	6.44593400	0.38660400	-1.05466200
C	4.99672800	0.13619900	-0.55209200
C	5.19261400	0.34424000	0.96164700
C	6.56339500	0.21821200	1.19053700
C	7.14824000	0.26994400	2.45094900
C	6.28559700	0.45716300	3.54709600
C	4.92059800	0.61316500	3.35390500
C	4.34949300	0.57180300	2.06561400
O	6.91574700	0.49353000	4.75687200
O	8.48715400	0.14655600	2.60103100
O	7.47977800	-2.80667400	-2.18370400
O	8.24295400	-0.26118300	-2.47435400
O	6.56564900	1.74166700	-1.37679900
O	7.30537700	0.04803000	0.05109000
H	4.31062400	0.85692800	-0.99765900
O	6.08326200	-0.20099900	-3.35469900
C	0.44126600	0.31854400	-2.13986900
C	0.87984700	-0.72015000	-1.09587800
C	0.56020900	-0.33812000	0.36023400
C	0.97337600	1.11206300	0.65599200
C	0.65886200	2.10467500	-0.46837600
O	0.90813100	1.62978100	-1.76013600
O	2.32531000	-0.69648000	-1.22890700
O	2.41242800	1.11697500	0.80148700
C	-1.04072500	0.34271200	-2.61454300
O	-0.84894800	-0.48960100	0.58746800
O	-0.71801600	2.50267900	-0.33191500
C	-0.97643300	3.84091500	-0.52621000
O	-0.08828100	4.66568600	-0.63552700
C	-2.41971000	4.15148900	-0.58838100
C	-2.77117100	5.50340100	-0.72086100
C	-4.11142200	5.86222500	-0.81115900
C	-5.09751900	4.86831300	-0.77485300
C	-4.74730000	3.51490100	-0.64480300
C	-3.40651600	3.15072800	-0.54655900
O	-4.46264900	7.17347500	-0.93714800
O	-6.39804100	5.26168800	-0.87112600
O	-5.81290900	2.66762900	-0.63970900
O	-1.98551900	-0.47319000	-1.89023900
C	-1.24000000	-1.32860600	1.57601000
O	-0.48446000	-1.93211000	2.30594000
C	-2.03394000	-1.78182400	-2.22595400
O	-1.26280300	-2.26812100	-3.03750400
C	-2.73570200	-1.38910200	1.67149000
C	-3.25037600	-1.85157100	2.83009000
C	-4.69526800	-1.84698800	3.06239300
C	-5.58519500	-1.52218200	1.84335100
C	-4.94326000	-0.41918100	0.99276800
C	-3.59406600	-0.95077900	0.48758000
C	-3.84643400	-2.16546800	-0.40312900
C	-4.87561500	-3.01301600	0.02405000

C	-5.18083500	-4.21409500	-0.62221900
C	-4.43217900	-4.60143700	-1.73875800
C	-3.41511900	-3.77151100	-2.19239300
C	-3.11804300	-2.55615800	-1.55408900
O	-4.70440100	-5.76512000	-2.38558500
O	-6.18242400	-5.03456100	-0.21317100
O	-5.23560700	-2.09505500	4.13059700
O	-6.84130700	-1.13866000	2.24423200
O	-5.69013300	-2.75887300	1.10657200
O	-5.75380500	-0.10115400	-0.12220700
O	-4.70561400	0.70746300	1.79010700
H	-3.08713100	-0.16660500	-0.06561600
H	4.93216400	-3.27631800	-1.46127400
H	4.26755600	0.78107700	4.20310000
H	6.28008600	0.64455800	5.47013100
H	8.68695100	0.21418600	3.54917900
H	8.62869100	-1.12962700	-2.69265100
H	7.50189400	1.90349300	-1.58236200
H	6.08146700	0.76596600	-3.45163500
H	1.04263800	0.10390100	-3.02559700
H	0.50471600	-1.71643500	-1.33214000
H	1.08734100	-1.01018700	1.03655600
H	0.51052300	1.44934400	1.58341200
H	1.28661300	2.98754300	-0.35534700
H	-1.41845300	1.35691300	-2.51123400
H	-1.07511200	0.04384000	-3.66207700
H	-2.00324500	6.26606100	-0.75594400
H	-3.12682800	2.11179200	-0.43856300
H	-5.42997500	7.22314700	-0.98960500
H	-6.95440700	4.46682300	-0.82470500
H	-5.56303000	1.73494700	-0.48073800
H	-2.60469100	-2.16888000	3.64118100
H	-2.84641200	-4.06170800	-3.06654400
H	-5.44187900	-6.20324600	-1.93219000
H	-6.61580300	-4.64150800	0.56133400
H	-6.96628800	-1.48877600	3.14881400
H	-6.68554900	-0.13549700	0.15391800
H	-5.53338000	0.93717800	2.24361900

Cartesian coordinates of the lowest-energy conformer of amariin [**1–C (**^{O,3}**B)**] at the B3LYP/6-31G(d,p) level in acetone (PCM).

C	0.18199900	-0.94190400	-2.36459100
C	0.84767000	-1.68223300	-1.18006500
C	0.40875600	-1.08099200	0.16288300
C	0.73501400	0.42380800	0.21257600
C	0.34496700	1.12385600	-1.11070800
O	-0.42345700	0.28877400	-1.95672700
O	2.26989200	-1.43670500	-1.31891500
O	2.15064700	0.57215600	0.41517900
C	-0.89416200	-1.75203100	-3.07996500
O	-1.02278800	-1.22473800	0.23303100
O	-0.52223500	2.18501200	-0.76702300
C	-0.51642800	3.31814400	-1.54343200
O	0.31587600	3.51932200	-2.40593000
C	-1.62000200	4.22755500	-1.17089500
C	-1.61211800	5.52507100	-1.70326300
C	-2.64075900	6.40525800	-1.38181000
C	-3.68260400	5.98228600	-0.54370400
C	-3.69405200	4.68027000	-0.02208100
C	-2.65789600	3.80173800	-0.32401500
O	-2.64257500	7.67037100	-1.89034300
O	-4.67778800	6.87193700	-0.27174900
O	-4.76372000	4.38790700	0.77477100
O	-1.99132800	-2.11757800	-2.21818500
C	3.12525600	-2.17502200	-0.58222800
O	2.81628400	-3.16260700	0.04932700
C	2.59228100	0.91923400	1.65748800
O	1.83483400	1.28977200	2.53375900
C	4.51572900	-1.61637800	-0.68066300
C	5.52874400	-2.49267100	-0.83851800
C	6.89045900	-2.02455300	-1.11068000
C	7.14477500	-0.50773400	-0.97079000
C	5.93425300	0.29781600	-1.46949400
C	4.72172600	-0.10430400	-0.59880400
C	4.98664300	0.28931700	0.85679300
C	6.32054200	0.16196200	1.25880500
C	6.75810800	0.48544600	2.54758900
C	5.83971400	0.96146900	3.48782900
C	4.50829600	1.09346800	3.11954500
C	4.06357900	0.76411800	1.82757500
O	6.23974000	1.29216000	4.74469100
O	8.05599500	0.36566900	2.92737900
O	7.82186400	-2.74876200	-1.43345200
O	8.29790800	-0.14489300	-1.61273800
O	7.33697500	-0.28627500	0.44949800
O	6.12503300	1.67536200	-1.33202100
O	5.77518000	-0.08102500	-2.81310200
H	3.84375600	0.41043900	-0.98004300
C	-1.60107300	-1.17846500	1.45188800
O	-0.99529200	-1.27476400	2.49703300
C	-1.98353100	-3.35492900	-1.67117000
O	-1.12248900	-4.18792400	-1.89851800
C	-3.06733500	-0.86059100	1.33376000
C	-3.60680100	-0.29762900	2.43596400
C	-4.95932100	0.25993000	2.45242400
C	-5.61955400	0.45979400	1.08143400
C	-5.32216700	-0.73965700	0.17467400
C	-3.82315000	-1.10798600	0.02681500
C	-3.96467000	-2.61177400	-0.22248300
C	-5.17891600	-2.97619600	0.35426700

C	-5.62874300	-4.28492700	0.43820300
C	-4.79465800	-5.28714200	-0.09357700
C	-3.60359400	-4.94349600	-0.72916200
C	-3.18147800	-3.60472000	-0.82712100
O	-5.17584700	-6.59038100	-0.03628600
O	-6.81223500	-4.67235800	0.98553100
O	-5.55945200	0.60282900	3.45973700
O	-6.98860900	0.61480100	1.20310200
O	-5.84875800	-0.52011200	-1.09898200
O	-5.92417600	-1.90282200	0.79291000
H	-3.35829100	-0.56718600	-0.79766900
O	-4.99305900	1.59936200	0.49470700
H	0.96935800	-0.74421000	-3.10643300
H	0.66220400	-2.75591100	-1.21070900
H	0.87593600	-1.59922400	1.00058200
H	0.20855400	0.88936100	1.04572800
H	1.23038800	1.50700400	-1.62945800
H	-0.45080500	-2.64968500	-3.51394200
H	-1.33588700	-1.13786600	-3.86568600
H	-0.81116200	5.84499400	-2.35873100
H	-2.66890500	2.79682900	0.07694300
H	-3.43634200	8.12447300	-1.56674100
H	-5.34601400	6.41709800	0.26684500
H	-4.86200900	3.41921200	0.87386500
H	5.35072700	-3.56239400	-0.86794100
H	3.79098900	1.45270000	3.84622300
H	7.19566400	1.14068300	4.81161300
H	8.57395600	0.05161800	2.16825400
H	8.81139200	-0.96704200	-1.73662400
H	6.93213200	1.89836900	-1.82331100
H	5.01225800	0.40022600	-3.16806900
H	-3.02204800	-0.21637500	3.34593000
H	-3.00937600	-5.72425700	-1.18813100
H	-6.03264300	-6.64152700	0.41624500
H	-7.31540200	-3.89583900	1.27306800
H	-7.15220400	0.91521300	2.11670500
H	-6.80876300	-0.40165000	-1.00050000
H	-5.23182000	1.58780300	-0.44924300

Cartesian coordinates of the lowest-energy conformer of amariin [**1–C ($^1C_4$)**] at the B3LYP/6-31G(d,p) level in acetone (PCM).

C	0.40710800	0.11538600	-2.24828600
C	0.87552400	-0.81061900	-1.11968400
C	0.59126000	-0.26718500	0.29514100
C	1.02961700	1.19812400	0.43312800
C	0.66621800	2.06879500	-0.77483600
O	0.86787600	1.46369600	-2.02268100
O	2.31397500	-0.80451400	-1.27831000
O	2.46936100	1.21308100	0.54257300
C	-1.09667200	0.08371300	-2.63478100
O	-0.81736200	-0.31864700	0.59258100
O	-0.70655400	2.46007000	-0.58886200
C	-1.05064000	3.69384500	-1.08725500
O	-0.24170800	4.42180300	-1.63157500
C	-2.48193300	4.01330200	-0.89728300
C	-3.34575600	3.19995200	-0.13951300
C	-4.68346200	3.57053500	-0.01119500
C	-5.15124200	4.73828400	-0.63597100
C	-4.28675600	5.54531900	-1.38462200
C	-2.95018400	5.18352600	-1.51308000
O	-5.64106900	2.89940800	0.68432700
O	-6.45274700	5.12683100	-0.53222900
O	-4.75077900	6.67778500	-1.98553900
O	-1.92565900	-0.81225100	-1.87128000
C	3.07485000	-1.74817300	-0.69198800
O	2.65774200	-2.76458500	-0.18113400
C	2.97954100	1.13344100	1.80554200
O	2.27433200	1.26010400	2.78956500
C	4.51899100	-1.34822800	-0.80319200
C	5.42526200	-2.31235700	-1.06232600
C	6.81416600	-1.96664300	-1.38032300
C	7.22958000	-0.49068200	-1.18674700
C	6.08243100	0.45915300	-1.56724800
C	4.88611600	0.12750200	-0.64697700
C	5.26966100	0.39847500	0.80860600
C	6.60454300	0.11935500	1.12235700
C	7.13665100	0.30162400	2.40293400
C	6.31399000	0.77399500	3.42969100
C	4.98023100	1.03888100	3.15349800
C	4.44265600	0.86217800	1.86635400
O	6.80668000	0.96898400	4.68205900
O	8.43641600	0.04074900	2.69517800
O	7.64956100	-2.76165300	-1.78810600
O	8.37389500	-0.21257100	-1.88434300
O	7.52993300	-0.35920700	0.22616600
O	6.42224400	1.80014900	-1.36627300
O	5.81004000	0.18020100	-2.91657600
H	4.04527800	0.74992100	-0.94313400
C	-1.27270600	-1.46495100	1.14721400
O	-0.58558200	-2.43179300	1.38773200
C	-2.03221900	-2.09346400	-2.29700900
O	-1.34096300	-2.55612300	-3.18673700
C	-2.72853900	-1.34279600	1.49627600
C	-3.04723100	-1.56429500	2.78823600
C	-4.40091500	-1.32809100	3.29216500
C	-5.30399200	-0.48047600	2.37767800
C	-5.18351800	-0.98667600	0.93616500
C	-3.73630600	-1.05485100	0.38820000
C	-3.84085900	-2.29755800	-0.50139200
C	-4.92685000	-3.01953900	-0.01607600

C	-5.29952800	-4.26326200	-0.50618400
C	-4.52035100	-4.81090600	-1.54382400
C	-3.45240900	-4.09097800	-2.07334500
C	-3.10064500	-2.82475800	-1.57249700
O	-4.83562000	-6.02700200	-2.06135900
O	-6.35960500	-4.98925700	-0.06287900
O	-4.81797300	-1.67642500	4.38783400
O	-6.61770900	-0.52322100	2.80426100
O	-5.95853300	-0.25107100	0.05604900
O	-5.62569400	-2.37468300	0.97711400
H	-3.48200600	-0.14653900	-0.15644400
O	-4.79573700	0.85339200	2.35576500
H	0.97740000	-0.18842600	-3.12821900
H	0.49484500	-1.82592200	-1.23636100
H	1.12041700	-0.88436600	1.02299600
H	0.59258400	1.62766200	1.33382300
H	1.28748300	2.96365400	-0.78802500
H	-1.18770100	-0.18011700	-3.68868000
H	-1.52739000	1.06670000	-2.46560000
H	-2.97574000	2.30928200	0.35337100
H	-2.27553400	5.80240500	-2.09142900
H	-5.29730400	2.09575800	1.12773100
H	-6.91538400	4.47102100	0.01507500
H	-5.69612500	6.76042000	-1.78452200
H	5.13346700	-3.35371000	-1.14783200
H	4.33509200	1.38575000	3.95034800
H	7.74805600	0.73447400	4.68120200
H	8.88170400	-0.26190000	1.88688800
H	8.79682200	-1.07374000	-2.06981300
H	7.21259300	1.97311800	-1.90277700
H	5.06449700	0.73641800	-3.18981600
H	-2.29709700	-1.88621100	3.50327200
H	-2.89724600	-4.50495600	-2.90622600
H	-5.61261900	-6.37059400	-1.59254900
H	-6.85147100	-4.48675600	0.60394400
H	-6.60435800	-0.94035900	3.68798600
H	-6.85706500	-0.20209500	0.42272000
H	-4.93740200	1.24972600	3.23018900

Cartesian coordinates of the lowest-energy conformer of amariin [**1–D (^{O,3}B)**] at the B3LYP/6-31G(d,p) level in acetone (PCM).

C	-2.12355200	-1.31159800	1.32318500
O	-1.60307200	-2.00221200	2.17052400
C	-2.67870600	-2.67232800	-2.08756800
O	-1.93193400	-3.54812100	-2.49123400
C	-3.54935900	-0.83728500	1.35451600
C	-4.04516500	-0.60185500	2.58724400
C	-5.33999400	0.05252000	2.79234600
C	-5.92474700	0.76093200	1.56163800
C	-5.76882900	-0.14025800	0.33180300
C	-4.32595900	-0.63894500	0.05370900
C	-4.63604400	-2.01043500	-0.55772100
C	-5.90294100	-2.35305400	-0.09218400
C	-6.50461200	-3.58147700	-0.32313600
C	-5.77436800	-4.52892000	-1.06340900
C	-4.52312600	-4.19852900	-1.57730500
C	-3.94366000	-2.93516000	-1.35456200
O	-6.30654900	-5.75346200	-1.31850700
O	-7.74457400	-3.93431400	0.11094400
O	-5.92729800	0.11386000	3.86214500
O	-5.14726500	1.92403400	1.38856800
O	-6.24495700	0.49703800	-0.81180500
O	-6.53014900	-1.34844000	0.60328200
H	-3.79794400	0.03643100	-0.61931700
O	-7.27793600	1.03412800	1.73971400
C	-0.13948000	-0.50784500	-2.31532500
C	0.32001100	-1.46732400	-1.18970100
C	-0.04846000	-0.90726700	0.19279300
C	0.57699300	0.49075600	0.38181000
C	0.54017200	1.29156500	-0.93327100
O	-0.46431000	0.78419500	-1.78909100
O	1.77224800	-1.48913100	-1.26942100
O	1.96544400	0.33443200	0.73292100
C	-1.33162000	-0.99704600	-3.12546700
O	-1.47937100	-0.77364600	0.26095200
O	0.18822300	2.62213100	-0.62368100
C	0.59059100	3.58979200	-1.51269200
O	1.25230500	3.33328900	-2.49991800
C	0.13640400	4.93753900	-1.11355600
C	-0.63924200	5.15048400	0.03545800
C	-1.03830000	6.44361100	0.36309800
C	-0.66266400	7.51912600	-0.45337800
C	0.11270100	7.29893000	-1.60114400
C	0.51358700	6.01234500	-1.93645400
O	-1.79297600	6.66064600	1.47653300
O	-1.07878300	8.76315200	-0.08765900
O	0.40871900	8.43097100	-2.30966900
O	-2.47368900	-1.35382100	-2.31831700
C	2.44256600	-2.41888400	-0.55741400
O	1.92580100	-3.32632600	0.05630600
C	2.24650800	0.04352700	2.03188100
O	1.41099300	0.10407600	2.91379300
C	3.92242600	-2.19624900	-0.70542900
C	4.63404500	-3.23758300	-1.18081300
C	6.03006800	-3.08454200	-1.60131400
C	6.48696400	-1.63545600	-1.84060300
C	6.02633400	-0.74621300	-0.67789600
C	4.51300000	-0.83834100	-0.32969200
C	4.57282900	-0.67176500	1.19976400
C	5.88518900	-0.96101800	1.56205600

C	6.34780500	-0.97518300	2.87054100
C	5.42206100	-0.68674500	3.88875500
C	4.10792300	-0.37088200	3.55979600
C	3.66338500	-0.34605700	2.22457700
O	5.81309900	-0.68687500	5.18995400
O	7.62929100	-1.24439600	3.23420500
O	6.79500300	-4.00639600	-1.84068700
O	5.83502900	-1.22538800	-3.02141700
O	6.33221800	0.59187100	-0.93065800
O	6.72012400	-1.21280100	0.50214000
H	3.96676900	-0.03648600	-0.82682700
O	7.87324900	-1.56085500	-1.93368700
H	-5.38981200	2.29637400	0.52466500
H	5.96113600	-0.26425800	-3.08881100
H	-3.46774200	-0.85757400	3.46948000
H	-3.99967600	-4.92187900	-2.19024400
H	-7.18202500	-5.80151600	-0.90300000
H	-8.15848100	-3.18817300	0.57019500
H	-7.17218300	0.73551100	-0.64303900
H	-7.42715000	1.05187300	2.70289200
H	0.69794800	-0.41237500	-3.02196600
H	-0.05949200	-2.48030300	-1.32564900
H	0.28163400	-1.57957200	0.98356700
H	0.04879100	1.02255900	1.17411100
H	1.51583400	1.26890800	-1.43080000
H	-1.67041900	-0.18758000	-3.77367700
H	-1.03262300	-1.85640800	-3.72674600
H	-0.93566000	4.32786000	0.67299600
H	1.11182700	5.82670700	-2.82181100
H	-1.97374300	7.61158400	1.54047000
H	-0.75146100	9.40364000	-0.73905800
H	0.95217500	8.21669600	-3.08030000
H	4.17132900	-4.20800500	-1.33067800
H	3.41135900	-0.12504900	4.35179000
H	6.75569300	-0.91358700	5.22911100
H	8.17580700	-1.37711700	2.44479800
H	7.29549100	0.64923600	-1.04892500
H	8.17715000	-2.45592300	-2.17270000

Cartesian coordinates of the lowest-energy conformer of amariin [**1–D ($^{1}C_{4}$)**] at the B3LYP/6-31G(d,p) level in acetone (PCM).

C	-1.34362000	-1.41160200	1.23390500
O	-0.68035400	-2.38754600	1.50272600
C	-1.97340500	-2.18552300	-2.17844600
O	-1.25644900	-2.68619000	-3.02595900
C	-2.80823400	-1.26640500	1.53419100
C	-3.16934300	-1.43537800	2.82271300
C	-4.53862600	-1.17818900	3.27074800
C	-5.40732600	-0.36197500	2.29592600
C	-5.24247700	-0.92283600	0.87901000
C	-3.77804100	-1.02146300	0.38352100
C	-3.85898200	-2.29963300	-0.45633500
C	-4.96891500	-2.99323800	0.01564300
C	-5.33400600	-4.25273200	-0.43875200
C	-4.52107700	-4.84733700	-1.42336200
C	-3.42544500	-4.15864700	-1.93756900
C	-3.08062200	-2.87681500	-1.47321600
O	-4.82837600	-6.08018500	-1.90490500
O	-6.41637300	-4.95271300	-0.00744200
O	-4.99462900	-1.48716600	4.36277900
O	-4.89262300	0.96837600	2.24087100
O	-5.98051500	-0.21483700	-0.05424000
O	-5.69820500	-2.30458900	0.95622800
H	-3.50054900	-0.13769200	-0.18934500
O	-6.73427700	-0.38356700	2.68131800
C	0.48554300	0.02192100	-2.11155300
C	0.91621100	-0.84835800	-0.92412800
C	0.56826300	-0.24616900	0.45061900
C	1.00906500	1.21998500	0.53741900
C	0.69349800	2.03891300	-0.71899600
O	0.93525900	1.38063700	-1.93167300
O	2.36176800	-0.84050800	-1.04642400
O	2.44539400	1.23425900	0.68608800
C	-1.00220900	-0.02931100	-2.55389900
O	-0.85310200	-0.27701500	0.68627100
O	-0.68264300	2.44433500	-0.59846800
C	-1.00106800	3.65646400	-1.16206600
O	-0.16833900	4.35765700	-1.70570800
C	-2.43689200	3.98936100	-1.04087500
C	-2.87387900	5.13608300	-1.72060500
C	-4.21229500	5.50843300	-1.65930000
C	-5.11027200	4.73553600	-0.91404800
C	-4.67415900	3.59175000	-0.22538300
C	-3.33459700	3.21075100	-0.28599500
O	-4.64580400	6.61803500	-2.32249200
O	-6.41266800	5.13321900	-0.87779900
O	-5.66222700	2.95266600	0.45787700
O	-1.86247500	-0.89308100	-1.78799000
C	3.12265400	-1.68662300	-0.32827600
O	2.70831200	-2.58575300	0.36932200
C	2.90916900	1.05729700	1.95453500
O	2.17473000	1.06096100	2.92484900
C	4.56632200	-1.37091700	-0.60822700
C	5.34927000	-2.40656500	-0.96945700
C	6.67849200	-2.19126900	-1.55117400
C	6.93045700	-0.77251200	-2.09302200
C	6.47849700	0.26534000	-1.05594100
C	5.02922600	0.08169900	-0.52125900
C	5.21821600	0.50599400	0.94660100
C	6.58625000	0.42717800	1.19230900

C	7.17476200	0.67270800	2.42492600
C	6.32476400	1.00767200	3.49294600
C	4.95405100	1.10842600	3.27957500
C	4.37921900	0.87568600	2.01517900
O	6.83828200	1.25805400	4.72585100
O	8.51025500	0.61046800	2.66930500
O	7.52745000	-3.05605600	-1.70356500
O	6.12466700	-0.65962800	-3.24376600
O	6.59268300	1.55836700	-1.56774900
O	7.33860500	0.09542500	0.09448000
H	4.34152100	0.72705400	-1.06844600
O	8.28221200	-0.57518400	-2.35803900
H	-5.06667500	1.39902100	3.09304200
H	6.12528200	0.27943000	-3.49310500
H	-2.44485500	-1.73054100	3.57480300
H	-2.84164900	-4.61073100	-2.73011000
H	-5.62697600	-6.39784900	-1.45450700
H	-6.92866000	-4.42020500	0.61947900
H	-6.89095300	-0.14657200	0.27835000
H	-6.75117000	-0.77247000	3.57780400
H	1.08872100	-0.32060600	-2.95452700
H	0.54738500	-1.87144900	-1.00913600
H	1.05796200	-0.83054900	1.22976700
H	0.54709800	1.69148000	1.40470400
H	1.32012000	2.92974200	-0.74807400
H	-1.43859200	0.95995700	-2.44490200
H	-1.05120700	-0.33768900	-3.59842400
H	-2.17344300	5.72852500	-2.29597700
H	-2.98971200	2.33912800	0.25659300
H	-5.59807400	6.71191100	-2.16338300
H	-6.90069000	4.50041800	-0.32552500
H	-5.34102600	2.16434400	0.94358100
H	4.98259000	-3.42696600	-0.91745600
H	4.31319500	1.38738400	4.10669800
H	7.80176100	1.15036600	4.68678100
H	8.98742300	0.41291900	1.84910100
H	7.52523700	1.69137100	-1.80798100
H	8.67084100	-1.46344100	-2.46105700

Cartesian coordinates of the lowest-energy conformer of geraniin [**2–A (^{O,3}B)**] at the B3LYP/6-31G(d,p) level in acetone (PCM).

C	-0.19203500	-0.45766100	2.00230000
C	-0.65097300	-1.15436500	0.69368100
C	-0.22255700	-0.34620100	-0.55088600
C	-0.66070100	1.11337900	-0.40445300
C	-0.15272100	1.70233100	0.93390400
O	0.52176200	0.75115700	1.73445600
O	-2.10363600	-1.18484300	0.75427600
O	-2.09854000	1.20140900	-0.37748400
C	0.71781700	-1.33441300	2.87957800
O	1.20777600	-0.26089400	-0.65262800
O	0.82553100	2.66990700	0.61365500
C	1.01643900	3.71066200	1.48782300
O	0.26741300	3.93432400	2.41874500
C	2.21211300	4.50105800	1.12796900
C	2.41426800	5.72454800	1.78206400
C	3.53266800	6.49194800	1.47076100
C	4.44913400	6.03064300	0.51591100
C	4.25032000	4.80038300	-0.12932400
C	3.12889100	4.03404300	0.17026900
O	3.73762800	7.68727900	2.09441500
O	5.53260700	6.81315400	0.24834300
O	5.21800000	4.46817600	-1.02902100
C	1.92665600	-1.32173800	-1.10754700
O	1.42669100	-2.33545500	2.12332000
O	1.42518000	-2.38361600	-1.41481000
C	2.71872600	-2.11933800	1.75536500
C	3.24476100	-3.31441900	1.02437300
O	3.33655900	-1.10939200	2.01716700
C	4.07903100	-3.18666200	-0.11205900
C	4.60526000	-4.37287900	-0.64562400
C	4.32123900	-5.62550100	-0.09924300
C	3.46694100	-5.72857900	1.00070200
C	2.92751900	-4.57272400	1.55513500
C	4.38010500	-1.88074500	-0.76734700
C	3.37502600	-0.96175600	-1.15759100
C	3.70424300	0.31330400	-1.64062100
C	5.03319500	0.68703100	-1.78423200
C	6.04135100	-0.22012500	-1.45610400
C	5.71628200	-1.48628400	-0.95602700
O	3.24988500	-6.99966900	1.44692800
O	4.89607200	-6.70953400	-0.69254900
O	5.44554800	-4.31118300	-1.73289200
O	6.80226900	-2.26218800	-0.63693800
O	7.33289400	0.16023400	-1.63909600
O	5.36263600	1.92975700	-2.27298200
C	-2.75833700	-1.91783400	-0.15993300
O	-2.22421200	-2.64493500	-0.97274300
C	-2.74809000	1.24478200	-1.56976600
O	-2.17631000	1.24051600	-2.64142800
C	-4.25028700	-1.77324200	-0.01855500
C	-4.92886600	-2.91751100	-0.24250600
C	-6.35354800	-3.05389900	0.04937500
C	-7.15568000	-1.75761500	0.21064600
C	-6.33941400	-0.68654100	0.98955100
C	-4.86556100	-0.48060900	0.49850000
C	-5.01424300	0.73735300	-0.43418800
C	-6.29781100	1.23343500	-0.20839900
C	-6.85600800	2.29726200	-0.90300000
C	-6.07274000	2.91833700	-1.88622700

C	-4.76864700	2.48133400	-2.10620900
C	-4.21301500	1.42254700	-1.36348200
O	-6.57334000	3.95206200	-2.61527200
O	-8.11952700	2.76575300	-0.69502700
O	-6.93235000	-4.13070400	0.13171100
O	-8.32760800	-2.01971600	0.92553800
O	-6.33996800	-1.00149300	2.32873000
O	-7.00713500	0.57950700	0.76008400
H	-4.28528700	-0.18817300	1.38279100
O	-7.40058400	-1.26015000	-1.08286500
H	-1.09350200	-0.22398800	2.58518500
H	-0.28262500	-2.17517800	0.61272100
H	-0.63477600	-0.80204800	-1.45154600
H	-0.27589900	1.69779500	-1.24081400
H	-0.97908300	2.16298300	1.48673600
H	1.42004800	-0.69788100	3.41937700
H	0.10745300	-1.90108500	3.58400500
H	1.70541700	6.07532400	2.52231400
H	2.97354900	3.07902700	-0.31485700
H	4.56286300	8.06705900	1.75427100
H	6.07861800	6.35496900	-0.41100800
H	5.08694200	3.56970500	-1.39375000
H	2.27547300	-4.64173500	2.41844100
H	2.92434600	1.00441800	-1.93507000
H	2.66660700	-6.99692800	2.21854700
H	4.59763100	-7.51210200	-0.23654200
H	5.70305400	-5.21740200	-1.97197900
H	6.65899200	-3.14570400	-1.03434800
H	7.88959100	-0.60673100	-1.41697500
H	6.32313500	1.94639900	-2.42136100
H	-4.39868300	-3.81501600	-0.54248600
H	-4.16038600	2.99825300	-2.83875200
H	-7.48952100	4.10675600	-2.33574500
H	-8.58776300	2.16714700	-0.09323800
H	-8.45608000	-2.98766000	0.88204600
H	-7.21839400	-1.37681900	2.52387500
H	-7.95814600	-1.89951500	-1.55243500

Cartesian coordinates of the lowest-energy conformer of geraniin [**2–A ($^1C_4$)**] at the B3LYP/6-31G(d,p) level in acetone (PCM).

C	-0.27965300	-0.19441800	-2.03362100
C	-0.72259300	0.66195700	-0.84101700
C	-0.39911700	0.02165200	0.52127100
C	-0.91571600	-1.42544500	0.56979600
C	-0.57854600	-2.23274200	-0.69320800
O	-0.76458200	-1.54790700	-1.90723200
O	-2.16431400	0.69821000	-0.99868700
O	-2.35364900	-1.38639100	0.67952300
C	1.22994100	-0.18708500	-2.35350100
O	1.02470800	-0.04267800	0.73536000
O	0.78784200	-2.62495400	-0.53555400
C	1.18843500	-3.86543000	-0.96579900
O	0.40565200	-4.74231900	-1.27532500
C	2.66382000	-3.94972900	-1.01456100
C	3.26779100	-5.14448800	-1.43438100
C	4.65199500	-5.19882300	-1.57302500
C	5.42886100	-4.05426100	-1.32505500
C	4.81907600	-2.86354400	-0.91024300
C	3.44392200	-2.82089500	-0.72162000
O	5.25158000	-6.35285400	-1.98343500
O	6.77367800	-4.14765800	-1.52113900
O	5.64935100	-1.79398600	-0.71494400
C	1.64944300	1.09186600	1.17700800
O	1.75075800	1.13378400	-2.08179400
O	1.08243000	2.15882700	1.27403000
C	2.99283200	1.21718000	-1.55203900
C	3.29177900	2.59512800	-1.07547500
O	3.74586900	0.25938500	-1.47635900
C	3.91822100	2.79843100	0.17907700
C	4.27721200	4.11652700	0.50692200
C	3.98453000	5.18413600	-0.34851900
C	3.31193800	4.96785600	-1.55631300
C	2.96820800	3.66794100	-1.91507600
C	4.11390000	1.69046700	1.15624800
C	3.07099800	0.81191100	1.53748800
C	3.31488400	-0.30508600	2.34910100
C	4.59402000	-0.54529000	2.84199000
C	5.63011900	0.33652800	2.52157900
C	5.38600400	1.42729400	1.68421100
O	3.02460600	6.00631000	-2.38730000
O	4.33442000	6.46057200	-0.03208200
O	4.89282100	4.47656000	1.67959400
O	6.42969600	2.26558600	1.37491000
O	6.90286300	0.17450100	2.97774800
O	4.95113500	-1.58129200	3.65369200
C	-2.91593600	1.54627500	-0.27631800
O	-2.49477100	2.40802700	0.46377200
C	-2.84781600	-1.24190300	1.94014600
O	-2.14303100	-1.30315300	2.93033900
C	-4.36084300	1.28877500	-0.60586600
C	-5.10057000	2.35764000	-0.96148100
C	-6.41986400	2.19901100	-1.58216800
C	-6.70268200	0.80237900	-2.16486600
C	-6.31135100	-0.27428300	-1.14286700
C	-4.87217800	-0.14911700	-0.56638000
C	-5.11292000	-0.60027900	0.88569200
C	-6.48423600	-0.48703000	1.09707800
C	-7.11293500	-0.74567100	2.30695000
C	-6.30212000	-1.13197000	3.38801100

C	-4.92942400	-1.26674900	3.20859700
C	-4.31373000	-1.01934800	1.96647500
O	-6.85546300	-1.39841300	4.60027000
O	-8.45239400	-0.65012100	2.51698300
O	-7.23700300	3.09391600	-1.73578800
O	-5.87070200	0.69076800	-3.29718800
O	-6.45156100	-1.55028800	-1.69029500
O	-7.19659800	-0.10597300	-0.01153000
H	-4.19002700	-0.80348400	-1.10980100
O	-8.05243900	0.65567500	-2.46961300
H	-0.82450400	0.19340400	-2.89740400
H	-0.31796500	1.67174000	-0.89656400
H	-0.84984300	0.61317800	1.31937900
H	-0.49950900	-1.93456900	1.43892400
H	-1.20746200	-3.12127700	-0.75923100
H	1.76487900	-0.91345500	-1.75333700
H	1.38294600	-0.41993000	-3.40993300
H	2.66688300	-6.01548000	-1.66727300
H	2.98860500	-1.90892700	-0.36660900
H	6.20501700	-6.18927700	-2.05537700
H	7.15760100	-3.26465300	-1.39375200
H	5.13573100	-0.97101300	-0.82155500
H	2.47598300	3.49602600	-2.86475400
H	2.49814700	-0.96642400	2.61696000
H	3.34964800	6.82232500	-1.97518500
H	4.81748700	6.42249300	0.81155800
H	5.64318200	3.86443700	1.82111100
H	7.22958500	1.93906300	1.82003300
H	6.94350200	-0.63014600	3.51837900
H	4.19569000	-2.16007700	3.82648900
H	-4.70381800	3.36428400	-0.87372700
H	-4.31954900	-1.58446700	4.04509400
H	-7.81431100	-1.26352100	4.53882000
H	-8.90071600	-0.41916500	1.68924900
H	-5.89823500	-0.24079800	-3.57184500
H	-7.38117700	-1.64803900	-1.95724100
H	-8.40917700	1.55847700	-2.56151700

Cartesian coordinates of the lowest-energy conformer of geraniin [**2–B (^{O,3}B)**] at the B3LYP/6-31G(d,p) level in acetone (PCM).

C	0.00957800	-0.71855300	-2.44162400
C	0.64647100	-1.53071400	-1.28010500
C	0.18329500	-1.00224900	0.09166900
C	0.46554900	0.51095900	0.19968000
C	0.03656300	1.25078400	-1.08679900
O	-0.70991100	0.41410300	-1.94813000
O	2.07152100	-1.28565100	-1.38367700
O	1.88559300	0.66446200	0.39193100
C	-0.95347700	-1.54189100	-3.30463600
O	-1.23564800	-1.24061700	0.18024800
O	-0.85253700	2.27786500	-0.69627800
C	-0.94228300	3.38985200	-1.49657800
O	-0.19022000	3.58709800	-2.43120600
C	-2.03173400	4.28817900	-1.05973400
C	-2.11261600	5.55247400	-1.66083100
C	-3.12317500	6.43035400	-1.28159300
C	-4.05409900	6.03900500	-0.31020800
C	-3.97784900	4.76964300	0.28422300
C	-2.96321500	3.89109400	-0.08378800
O	-3.21004700	7.66403300	-1.85651100
O	-5.03207800	6.92676500	0.02494300
O	-4.94585300	4.52541500	1.21188500
C	-1.82378600	-1.00300800	1.38674900
O	-1.59511500	-2.57564700	-2.54057400
O	-1.18451300	-0.81980000	2.40284900
C	-2.80410500	-2.32493500	-1.97432300
C	-3.15194600	-3.41768800	-1.02109400
O	-3.48193300	-1.35074300	-2.22305600
C	-3.80221000	-3.10928400	0.19307300
C	-4.09321900	-4.17203900	1.06979200
C	-3.76006100	-5.49067100	0.73855700
C	-3.11024300	-5.77089200	-0.46604100
C	-2.79598000	-4.73424600	-1.34024200
C	-4.17937100	-1.72858800	0.63149700
C	-3.30351800	-0.82728600	1.26991700
C	-3.77393400	0.38077100	1.80624000
C	-5.11057400	0.73090600	1.66240100
C	-5.99425700	-0.13628500	1.01979000
C	-5.52913400	-1.36146800	0.53196500
O	-2.83348400	-7.09077100	-0.67642600
O	-4.08429200	-6.46100200	1.64072600
O	-4.70129600	-3.91710900	2.25907900
O	-6.49103000	-2.14508500	-0.03681000
O	-7.29730800	0.24874600	0.90989100
O	-5.56363600	1.93856800	2.13925300
C	2.91665900	-2.01624000	-0.63335500
O	2.61272100	-3.01891500	-0.02200800
C	2.33188400	1.15765300	1.57875300
O	1.58437700	1.64514000	2.40484100
C	4.29954700	-1.43224400	-0.69245800
C	5.33006700	-2.29471400	-0.80805300
C	6.68560700	-1.80610400	-1.06406500
C	6.90849600	-0.28109600	-0.95951600
C	5.69368800	0.49646800	-1.48555000
C	4.48137900	0.08455200	-0.63107100
C	4.73857900	0.50935500	0.81938200
C	6.07236000	0.41547300	1.23775600
C	6.49183300	0.77006000	2.52384400
C	5.55816500	1.25433400	3.44412600

C	4.23076500	1.36764800	3.05648000
C	3.80276600	1.00132800	1.76898700
O	5.94035900	1.61488000	4.69833100
O	7.78764700	0.67762000	2.91980200
O	7.63811200	-2.51598600	-1.35787700
O	8.04291600	0.08508300	-1.64486400
O	7.11196200	-0.01907700	0.44527000
O	5.87471700	1.87818900	-1.35138500
O	5.44842800	0.10825400	-2.81411200
H	3.60191100	0.57929200	-1.03286700
H	0.82790600	-0.37218300	-3.08822600
H	0.45081900	-2.59908700	-1.36449600
H	0.69729100	-1.52626700	0.89834200
H	-0.05016100	0.93310200	1.06004600
H	0.90336400	1.67440100	-1.60719200
H	-1.69384300	-0.88260100	-3.75995500
H	-0.39225600	-2.06605900	-4.07949700
H	-1.39242100	5.84792600	-2.41406400
H	-2.90042400	2.90792400	0.36555800
H	-3.97428200	8.12222500	-1.47318500
H	-5.60965600	6.50122800	0.67967200
H	-4.96674600	3.58869200	1.49471000
H	-2.28239000	-4.94574400	-2.27110700
H	-3.09648000	1.05421300	2.31770500
H	-2.33722100	-7.20940900	-1.49818500
H	-3.75886300	-7.31365700	1.31184500
H	-4.81264400	-4.76071200	2.72645200
H	-6.07588200	-2.92211600	-0.44263100
H	-7.78740500	-0.44582700	0.44218600
H	-6.52663700	1.97089300	2.01207600
H	5.17016100	-3.36746900	-0.82514300
H	3.50252000	1.74228100	3.76420500
H	6.89711000	1.47551900	4.77864800
H	8.31871300	0.35288000	2.17460000
H	8.58877700	-0.72233200	-1.72555000
H	6.74833500	2.10523900	-1.70688400
H	6.27999100	0.20691200	-3.30546400

Cartesian coordinates of the lowest-energy conformer of geraniin [**2–B ($^1C_4$)**] at the B3LYP/6-31G(d,p) level in acetone (PCM).

C	-0.14593400	0.04391900	-2.19462700
C	-0.66662500	0.84600100	-0.99802900
C	-0.34309100	0.17475600	0.34983600
C	-0.80200600	-1.29297500	0.34784300
C	-0.45667200	-2.05097400	-0.94535500
O	-0.59466500	-1.32253900	-2.13663400
O	-2.10347300	0.81630500	-1.16833300
O	-2.24018400	-1.31415300	0.47310800
C	1.37522400	0.10798300	-2.41672500
O	1.07735400	0.14877800	0.57276400
O	0.88476300	-2.54022600	-0.76450500
C	1.19213000	-3.71429800	-1.40427000
O	0.40467900	-4.27829300	-2.14096000
C	2.55174100	-4.20137800	-1.08327900
C	2.99213400	-5.34527700	-1.76507700
C	4.25011900	-5.87110600	-1.49150600
C	5.05997200	-5.25931900	-0.52675400
C	4.61863800	-4.11669100	0.15975600
C	3.36526900	-3.57742400	-0.11945200
O	4.68692500	-6.98142500	-2.15180300
O	6.28003800	-5.81226500	-0.27891200
O	5.51856800	-3.63791900	1.06481500
C	1.67383200	1.30263500	1.00638900
O	1.78052600	1.47303300	-2.17167000
O	1.06568900	2.34375000	1.14041600
C	3.04835600	1.65382700	-1.72379400
C	3.24082200	3.03456000	-1.18175400
O	3.89316100	0.78345600	-1.74832000
C	3.81348900	3.21305500	0.09880400
C	4.08864000	4.52870300	0.49405900
C	3.78251900	5.62187000	-0.31814800
C	3.16673700	5.42088000	-1.55729200
C	2.89468400	4.12449900	-1.98759400
C	4.09303800	2.06052200	1.00203900
C	3.11632100	1.07541700	1.29641600
C	3.46516000	-0.12229200	1.93305100
C	4.77752100	-0.34769500	2.32350700
C	5.73822700	0.64584600	2.12949500
C	5.39479700	1.83858000	1.47880400
O	2.89926000	6.55978000	-2.25928600
O	4.09490700	6.86062300	0.15873100
O	4.69218100	4.75081100	1.70898000
O	6.43861600	2.70638000	1.29738800
O	7.00264300	0.41291200	2.56669100
O	5.12027900	-1.54454600	2.90684000
C	-2.88215600	1.69624000	-0.51398900
O	-2.48627600	2.67140900	0.08743500
C	-2.73737200	-1.32493600	1.74199200
O	-2.02111700	-1.50398400	2.70998300
C	-4.32162700	1.29298500	-0.66953100
C	-5.23880100	2.26442300	-0.85213900
C	-6.62481500	1.92927200	-1.19086200
C	-7.02524000	0.43849400	-1.10765300
C	-5.87046900	-0.46863600	-1.56121600
C	-4.67354800	-0.19286300	-0.62418300
C	-5.04635200	-0.56798800	0.81089900
C	-6.38309900	-0.32953900	1.14893400
C	-6.90415600	-0.60632800	2.41703900
C	-6.06726300	-1.13163100	3.40602400

C	-4.73046400	-1.35394300	3.10712200
C	-4.20464800	-1.08395300	1.83167000
O	-6.54940100	-1.41730000	4.64509900
O	-8.20612400	-0.38667800	2.73355800
O	-7.47118300	2.74383100	-1.53302200
O	-8.16931400	0.20311000	-1.82121500
O	-7.31980300	0.19633400	0.29177700
O	-6.19573400	-1.82430800	-1.45840600
O	-5.60735200	-0.08806700	-2.88756700
H	-3.82733700	-0.78334300	-0.96661900
H	-0.65008300	0.44632700	-3.07693500
H	-0.30783600	1.87342100	-1.01390600
H	-0.82597000	0.72278000	1.16187900
H	-0.35905800	-1.81614200	1.19448300
H	-1.12376600	-2.90542800	-1.05884200
H	1.91073600	-0.55267700	-1.73861600
H	1.61302700	-0.17275500	-3.44579100
H	2.35531000	-5.82022700	-2.50109700
H	3.02296200	-2.68962000	0.39733100
H	5.57403300	-7.19787300	-1.82453300
H	6.70256900	-5.29564000	0.42686400
H	5.15816100	-2.93290500	1.63844500
H	2.44177700	3.95991500	-2.95951700
H	2.71194900	-0.86898100	2.15152700
H	2.48804800	6.34964600	-3.10932700
H	3.81554800	7.52587200	-0.48958900
H	4.81176100	5.70844700	1.82353900
H	6.11721500	3.61012800	1.49415600
H	7.52175800	1.21703300	2.39194700
H	6.03866100	-1.48076500	3.21851800
H	-4.95889900	3.31249700	-0.85460300
H	-4.07425600	-1.74030200	3.87634200
H	-7.49472200	-1.20001200	4.66353900
H	-8.66208400	-0.03987200	1.94941100
H	-8.59949900	1.07242600	-1.94076300
H	-6.98870800	-1.96494500	-2.00044000
H	-4.86120200	-0.61879200	-3.20570500

Cartesian coordinates of the lowest-energy conformer of 3,6-dimethoxyfurosin [**fro–A (^{O,3}B)**] at the B3LYP/6-31G(d,p) level in acetone (PCM).

C	1.20595100	3.37621300	-0.47903300
C	-0.04777800	3.19102200	0.39869400
C	0.30372200	2.43768500	1.69054000
C	0.86122300	1.03834600	1.32405800
C	1.65658800	1.08477400	0.00888300
O	2.19370800	2.37594400	-0.19420700
O	-0.93611400	2.36665300	-0.42368100
O	-0.23821200	0.11544900	1.11396200
C	1.89305800	4.72419000	-0.27734100
O	1.26495600	3.20803400	2.38597800
O	2.75646000	0.20246500	0.13251600
C	3.26815800	-0.31378300	-1.03006700
O	2.78581200	-0.07691400	-2.12137400
C	4.44175400	-1.18030100	-0.78896300
C	4.95895300	-1.39156800	0.49737400
C	6.06793700	-2.21729300	0.66318300
C	6.65764200	-2.82864500	-0.45159600
C	6.13526800	-2.61306100	-1.73496600
C	5.02974200	-1.79092800	-1.90928500
O	6.57810700	-2.42920100	1.90905300
O	7.74053800	-3.62596500	-0.23372000
O	6.80061700	-3.26678500	-2.73583700
O	0.98973100	5.72297200	-0.71496600
C	-2.08095800	1.86285500	0.06253900
O	-2.59757100	2.13873100	1.12378300
C	-0.86787300	-0.33138500	2.23239800
O	-0.42854500	-0.13179600	3.35153500
C	-2.67834400	0.89621300	-0.92276000
C	-3.20357200	1.42254200	-2.04564400
C	-3.86845900	0.58786800	-3.04255700
C	-4.46929600	-0.72284000	-2.51060900
C	-3.45693000	-1.48222500	-1.59459100
C	-2.52402000	-0.60683500	-0.68471800
C	-2.75869800	-1.19856200	0.70199200
C	-3.80970500	-2.10615900	0.58426100
C	-4.32089500	-2.84272000	1.64898700
C	-3.70615700	-2.66872900	2.90124800
C	-2.60940200	-1.82939900	3.03794500
C	-2.10249900	-1.10037300	1.94477300
O	-4.25845700	-3.40835000	3.90727700
O	-5.35868000	-3.69279900	1.46557000
O	-4.03582400	0.89012400	-4.21552600
O	-4.78385000	-1.56775600	-3.58462300
O	-2.65794000	-2.31703500	-2.34623500
O	-4.29937900	-2.25095600	-0.68508000
H	-1.49960500	-0.83212000	-1.00467900
O	-5.59583200	-0.31326100	-1.79003100
C	1.18505900	3.07759500	3.80555900
C	1.50280300	7.03472600	-0.54292000
H	0.88795700	3.29776500	-1.52763800
H	-0.53187100	4.14747400	0.60135700
H	-0.59455500	2.31252300	2.29781300
H	1.49208100	0.67475500	2.13562500
H	1.03767400	0.78315500	-0.84099700
H	2.15735400	4.85720000	0.77920800
H	2.81976700	4.73674400	-0.87065100
H	4.51309900	-0.92311400	1.36505800
H	4.61497700	-1.61573300	-2.89582200

H	7.33934600	-3.02498400	1.82761000
H	8.04168500	-3.97930900	-1.08577500
H	6.39125700	-3.08036400	-3.59202900
H	-3.12780000	2.48448000	-2.25823900
H	-2.10962100	-1.73729500	3.99573700
H	-3.78629100	-3.26058200	4.73834700
H	-5.55476900	-4.10894400	2.32099900
H	-4.81089100	-0.99175700	-4.37363700
H	-3.20026500	-2.59094100	-3.11041600
H	-5.82422100	-1.05269800	-1.20037700
H	0.23228800	3.46959900	4.18478600
H	1.28749000	2.03385500	4.12423400
H	2.00704600	3.66411700	4.21910300
H	1.72037700	7.24806200	0.51343500
H	2.42302300	7.18677600	-1.12517800
H	0.73863100	7.72954400	-0.89734500

Cartesian coordinates of the lowest-energy conformer of 3,6-dimethoxyfurosin [**fro–A ($^1C_4$)**] at the B3LYP/6-31G(d,p) level in acetone (PCM).

C	-1.10752000	-2.19140700	-0.91702200
C	-0.36490200	-2.11283900	0.41631200
C	-0.88369000	-0.98170700	1.33152800
C	-0.93524000	0.33459000	0.54660700
C	-1.60078600	0.20212100	-0.83101800
O	-1.19565000	-0.90654600	-1.56407900
O	1.01721300	-1.82877400	0.04950100
O	0.41724300	0.76449200	0.26181800
C	-2.47441900	-2.88093300	-0.82996300
O	-2.18853700	-1.25391200	1.80704000
O	-3.03971900	0.14754800	-0.67132400
C	-3.68348100	1.33977600	-0.55886700
O	-3.08591100	2.40347900	-0.51625900
C	-5.15562700	1.19603100	-0.49102100
C	-5.90954800	2.36529500	-0.31844500
C	-7.29669700	2.28914400	-0.24498500
C	-7.93044500	1.04300300	-0.34510000
C	-7.17212400	-0.12282900	-0.51957900
C	-5.78512700	-0.05568600	-0.59345400
O	-8.03600900	3.42114200	-0.07455500
O	-9.29065100	1.01919300	-0.26622700
O	-7.91175300	-1.27114600	-0.60532600
O	-2.23040600	-4.21304400	-0.40743300
C	1.95420100	-1.88144000	1.00324900
O	1.77366800	-2.27757500	2.13771300
C	1.07468600	1.43466600	1.23960200
O	0.57062800	1.72417800	2.30780800
C	3.29776100	-1.46053300	0.46587300
C	4.29858000	-2.29209900	0.82249100
C	5.61988100	-2.23510400	0.20837000
C	5.99784700	-0.93779800	-0.51392900
C	4.78966000	-0.31374700	-1.28636800
C	3.41340600	-0.31441800	-0.53285900
C	3.31179400	1.13760400	-0.03054000
C	4.38586800	1.82626500	-0.59607500
C	4.68596000	3.15767600	-0.32393200
C	3.84018300	3.83906700	0.56539300
C	2.72554600	3.20422700	1.10023600
C	2.42295500	1.86683000	0.77917200
O	4.20173900	5.13206100	0.81349700
O	5.77033700	3.74589500	-0.88952600
O	6.42557600	-3.15511800	0.23830600
O	6.99446500	-1.20014000	-1.46376700
O	4.63476000	-0.92483600	-2.50814300
O	5.15935400	1.08659600	-1.44767200
H	2.64378300	-0.48776200	-1.29506000
O	6.43931600	-0.09811300	0.51811600
C	-2.23120500	-2.09662200	2.95882400
C	-3.41978200	-4.98049500	-0.30869800
H	-0.48782400	-2.78380400	-1.59584700
H	-0.40841200	-3.07918200	0.92100900
H	-0.19601700	-0.85268100	2.17314100
H	-1.44275700	1.09886500	1.13501200
H	-1.35535700	1.07951800	-1.43087700
H	-3.13860300	-2.36303300	-0.13029100
H	-2.93192400	-2.85855600	-1.83048700
H	-5.41536400	3.32580000	-0.23991600
H	-5.20139100	-0.95795900	-0.73246400
H	-8.97231800	3.16925100	-0.04344400

H	-9.58910000	0.09908800	-0.34257900
H	-7.33366900	-2.03914000	-0.70934400
H	4.10183900	-3.12811700	1.48526400
H	2.04528700	3.75338600	1.74222000
H	3.58420200	5.54827300	1.43069600
H	5.81556600	4.65834000	-0.56046800
H	7.44220900	-2.01325100	-1.16109200
H	5.52831600	-1.19105000	-2.79496100
H	6.67089600	0.74792100	0.10065800
H	-1.88054800	-3.11232900	2.74025300
H	-1.63015400	-1.67813700	3.77592600
H	-3.27649700	-2.14390200	3.26821300
H	-3.92749500	-5.06270100	-1.28053300
H	-3.13356000	-5.97884500	0.02857600
H	-4.12281300	-4.54485800	0.41565600

Cartesian coordinates of the lowest-energy conformer of 3,6-dimethoxyfurosin [**fro–B (^{O,3}B)**] at the B3LYP/6-31G(d,p) level in acetone (PCM).

C	1.05934100	3.24637700	-0.49028200
C	-0.16624100	3.00003600	0.40988900
C	0.21461100	2.21537000	1.67421900
C	0.87379300	0.86779200	1.27314700
C	1.70840900	1.01452600	-0.01046400
O	2.13201800	2.34866900	-0.18098800
O	-1.07926800	2.14875100	-0.34182500
O	-0.15391200	-0.10106100	0.97024000
C	1.63146400	4.65164400	-0.32194900
O	1.09543500	3.01517600	2.43413200
O	2.87534500	0.22868300	0.13660600
C	3.47984800	-0.20500300	-1.01531500
O	3.02492200	0.02292400	-2.12025300
C	4.71354000	-0.97290900	-0.74355500
C	5.18355500	-1.18697800	0.56038700
C	6.35159500	-1.91911500	0.75661400
C	7.04703900	-2.43443300	-0.34561000
C	6.57202800	-2.21570200	-1.64684200
C	5.40750500	-1.48730200	-1.85166200
O	6.81646300	-2.13276100	2.01966500
O	8.18380300	-3.14295900	-0.09812300
O	7.34271500	-2.77032900	-2.63176100
O	0.64858500	5.56329500	-0.77425000
C	-2.37187100	2.11257600	0.03487800
O	-2.89054600	2.88959300	0.80893600
C	-0.76910000	-0.71300000	2.01111900
O	-0.31861600	-0.67538400	3.14298800
C	-3.09587500	0.98665900	-0.65029600
C	-4.34250100	1.22990200	-1.10424700
C	-5.05832500	0.23261500	-1.90346500
C	-4.42381200	-1.17276400	-1.99362300
C	-2.89200700	-1.07493500	-2.07220700
C	-2.40850400	-0.37124900	-0.78400000
C	-2.76507200	-1.23292400	0.43034700
C	-3.98220900	-1.91453900	0.32086400
C	-4.47859200	-2.74415200	1.33155800
C	-3.74685200	-2.90633600	2.51173100
C	-2.54303100	-2.23023400	2.65516700
C	-2.03862700	-1.40062100	1.63974000
O	-4.20387400	-3.71219800	3.50756100
O	-5.65530900	-3.41229200	1.21374800
O	-6.11281000	0.43844000	-2.48887700
O	-4.94628500	-1.87580700	-3.04550200
O	-4.81565200	-1.84640700	-0.77019100
O	-2.28565900	-2.33334500	-2.12910500
O	-2.63601100	-0.31412500	-3.22520300
H	-1.33274700	-0.23679700	-0.85265900
H	0.74493800	3.11007300	-1.53456800
H	-0.66649600	3.93673200	0.65463100
H	-0.69851800	2.00088400	2.24347400
H	1.49584500	0.50562600	2.09206500
H	1.14398700	0.67536400	-0.88570700
H	1.88390900	4.82445300	0.73326000
H	2.55490000	4.73102000	-0.91459400
H	4.65575000	-0.79255500	1.41880200
H	5.02752600	-1.31084500	-2.85196300
H	7.63006200	-2.65757900	1.95878700
H	8.55748500	-3.43506700	-0.94465200
H	6.96457800	-2.58494400	-3.50226200

H	-4.81282800	2.19814000	-0.97008100
H	-1.97982300	-2.33913700	3.57329200
H	-5.05063700	-4.09443100	3.22808800
H	-6.03820900	-3.21570400	0.34323800
H	-5.78255600	-1.42876800	-3.28215300
H	-2.65223500	-2.78735200	-2.90493600
H	-1.67579600	-0.20531000	-3.30106600
C	1.04562300	6.91684000	-0.61650900
H	1.23935000	7.16016100	0.43791300
H	0.22531100	7.53813900	-0.98153700
H	1.95139400	7.14095300	-1.19789700
C	1.02595100	2.77265600	3.83952100
H	0.04051500	3.04760700	4.23762000
H	1.79062400	3.39798400	4.30268900
H	1.22283000	1.72167400	4.08289800

Cartesian coordinates of the lowest-energy conformer of 3,6-dimethoxyfurosin [**fro–B ($^{1}C_{4}$)**] at the B3LYP/6-31G(d,p) level in acetone (PCM).

C	-1.19406300	-2.44228300	-0.32919800
C	-0.34969300	-2.07687500	0.89091900
C	-0.74931500	-0.73160000	1.52582900
C	-0.83693400	0.37056000	0.44717300
C	-1.54449800	-0.08105800	-0.83966500
O	-1.22336200	-1.35916200	-1.27959000
O	0.98854300	-1.93424000	0.35163700
O	0.51055400	0.74651400	0.06844100
C	-2.59453200	-2.97204700	-0.00227000
O	-1.98953700	-0.91718400	2.17899600
O	-2.98004100	-0.02430600	-0.63594500
C	-3.57789400	1.18073700	-0.83427600
O	-2.94036200	2.19552400	-1.06618900
C	-5.05373200	1.11367700	-0.73359600
C	-5.76392900	2.31804000	-0.83491200
C	-7.15282800	2.30995100	-0.75322100
C	-7.83206400	1.09682400	-0.57472500
C	-7.11732700	-0.10503700	-0.47735600
C	-5.72883800	-0.10498800	-0.55332100
O	-7.84998400	3.47693700	-0.84776900
O	-9.19217000	1.14066600	-0.50373600
O	-7.89832000	-1.21632700	-0.30926400
O	-2.40750600	-4.20057700	0.68143800
C	2.05201100	-1.89433600	1.16757300
O	2.04716900	-2.18127700	2.34663400
C	1.08984700	1.72715100	0.80989800
O	0.45242700	2.39499800	1.60521800
C	3.25883600	-1.46019700	0.38273800
C	4.43977100	-2.05824800	0.63919700
C	5.59688400	-1.81398500	-0.22373500
C	5.46176200	-0.69720100	-1.28631500
C	4.03770800	-0.63405100	-1.85473800
C	3.08746700	-0.37072600	-0.67456500
C	3.43465300	0.98294300	-0.04837600
C	4.79467400	1.31979300	-0.06688700
C	5.28800500	2.51081000	0.47413000
C	4.40519000	3.41185800	1.07616300
C	3.05572700	3.09424900	1.13155000
C	2.55395600	1.90200600	0.58134200
O	4.85771700	4.58028800	1.60488700
O	6.60588600	2.83797100	0.43829400
O	6.64918600	-2.43681000	-0.16867700
O	6.37197900	-0.89244900	-2.29868000
O	5.78784100	0.53542000	-0.61074200
O	3.90049200	0.41702500	-2.77014500
O	3.71501400	-1.88344000	-2.41116100
H	2.06857700	-0.37775200	-1.05066700
H	-0.66495400	-3.23892500	-0.85920100
H	-0.38064900	-2.87305000	1.63606800
H	0.02302100	-0.44444100	2.25050800
H	-1.34546400	1.24935400	0.84025800
H	-1.28426300	0.60324300	-1.64829100
H	-3.15982700	-2.26290900	0.61087900
H	-3.13249700	-3.12282000	-0.95059000
H	-5.23575000	3.25307400	-0.97561300
H	-5.17748500	-1.03474700	-0.48045100
H	-8.79512900	3.27216500	-0.77124200
H	-9.52578700	0.23705600	-0.38674300
H	-7.35158600	-2.01297000	-0.26965600

H	4.53759000	-2.81137400	1.41381000
H	2.37135100	3.77926300	1.61516700
H	5.81797800	4.62376300	1.47355200
H	7.09074300	2.12846800	-0.01370900
H	7.07990400	-1.45591600	-1.92746400
H	4.63640700	0.36763900	-3.40033300
H	4.43286300	-2.12554300	-3.01830000
C	-3.63187200	-4.78732800	1.09220300
H	-4.27971400	-5.01222400	0.23249100
H	-4.18299400	-4.13152900	1.78143500
H	-3.38768500	-5.71945100	1.60607000
C	-2.19635100	-0.04776900	3.29228800
H	-2.15175300	1.00905100	3.00307300
H	-1.45112600	-0.23194700	4.07668300
H	-3.19170100	-0.26926700	3.68000400
